# Quasi-graphitic carbon shell-induced Cu confinement promotes electrocatalytic CO_2_ reduction toward C_2+_ products

**DOI:** 10.1038/s41467-021-24105-9

**Published:** 2021-06-21

**Authors:** Ji-Yong Kim, Deokgi Hong, Jae-Chan Lee, Hyoung Gyun Kim, Sungwoo Lee, Sangyong Shin, Beomil Kim, Hyunjoo Lee, Miyoung Kim, Jihun Oh, Gun-Do Lee, Dae-Hyun Nam, Young-Chang Joo

**Affiliations:** 1 Department of Materials Science and Engineering, Seoul National University, Seoul, Republic of Korea; 2 Department of Chemical and Biomolecular Engineering, Korea Advanced Institute of Science and Technology, Daejeon, Republic of Korea; 3 Department of Materials Science and Engineering, Korea Advanced Institute of Science and Technology, Daejeon, Republic of Korea; 4 Research Institute of Advanced Materials (RIAM), Seoul National University, Seoul, Republic of Korea; 5 Department of Energy Science and Engineering, Daegu Gyeongbuk Institute of Science and Technology (DGIST), Daegu, Republic of Korea; 6 Advanced Institute of Convergence Technology, Suwon, Republic of Korea

**Keywords:** Heterogeneous catalysis, Electrocatalysis, Nanoscale materials

## Abstract

For steady electroconversion to value-added chemical products with high efficiency, electrocatalyst reconstruction during electrochemical reactions is a critical issue in catalyst design strategies. Here, we report a reconstruction-immunized catalyst system in which Cu nanoparticles are protected by a quasi-graphitic C shell. This C shell epitaxially grew on Cu with quasi-graphitic bonding via a gas–solid reaction governed by the CO (g) - CO_2_ (g) - C (s) equilibrium. The quasi-graphitic C shell-coated Cu was stable during the CO_2_ reduction reaction and provided a platform for rational material design. C_2+_ product selectivity could be additionally improved by doping *p*-block elements. These elements modulated the electronic structure of the Cu surface and its binding properties, which can affect the intermediate binding and CO dimerization barrier. B-modified Cu attained a 68.1% Faradaic efficiency for C_2_H_4_ at −0.55 V (vs RHE) and a C_2_H_4_ cathodic power conversion efficiency of 44.0%. In the case of N-modified Cu, an improved C_2+_ selectivity of 82.3% at a partial current density of 329.2 mA/cm^2^ was acquired. Quasi-graphitic C shells, which enable surface stabilization and inner element doping, can realize stable CO_2_-to-C_2_H_4_ conversion over 180 h and allow practical application of electrocatalysts for renewable energy conversion.

## Abstract (web summary)

Surface reconstruction of electrocatalysts is an important issue for electroconversion of carbon dioxide to value-added chemical products. Here the authors address this issue by using copper nanoparticles protected by self-formed quasi graphitic carbon shell for stable CO2 to C2H4 conversion.

## Introduction

The electrochemical CO_2_ reduction reaction (CO_2_RR), which converts CO_2_ to value-added chemical products with high energy density, is a promising solution for fossil fuel-induced energy and environmental issues because it can help realize a sustainable energy cycle^1–3^. A commercially feasible CO_2_RR requires the development of an electrocatalyst that can steer the reaction pathway toward specific products on a multidimensional scale by controlling the binding energy of the reaction intermediates to design a reaction configuration. This enables economic earnings to be achieved by renewable energy conversion-induced electrochemical reactions in terms of energy efficiency, current density, and stability^4,5^.

Copper (Cu) has been extensively explored because of its unique ability to induce CO* dimerization (where * represents the adsorption state) for the production of ethylene (C_2_H_4_)^6–8^, ethanol (C_2_H_5_OH)^9,10^, and n-propanol (C_3_H_7_OH)^11,12^. To enhance C_2+_ product selectivity and catalytic activity, various strategies that alter the microstructure^10,13^, hydrophobicity^14^, defects^15^, facet control^13,16–18^, and doping^19,20^ of electrocatalysts have been applied to modify binding affinity and enrich the reaction intermediates. However, the major bottleneck that slows further progress toward achieving maximum C_2+_ product selectivity is the structural instability of the catalyst: electrocatalyst reconstruction occurs under an electrochemical reduction potential with a high current density^21,22^. The vigorous electrocatalytic reaction in the three-phase region (gas–liquid–solid) decreases the stability of the electrocatalyst^23–25^. This results in a gap between precatalysts (before reactions) and real catalysts (during reactions) in electrochemical CO_2_RRs. Although several in situ/operando spectroscopic analyses have been conducted, it is difficult to precisely track and define the changes in structure and phase during the reaction. This makes the rational material design of electrocatalysts challenging while also making it difficult to understand the fundamental reaction mechanism.

Herein, we report the development of a surface-stabilized catalyst system by exploiting a spontaneous graphitic carbon (C) deposition reaction that prevents electrocatalyst reconstruction during the CO_2_RR. The ability of the C coating to achieve electrochemical functionalities has been reported^26–28^, but we discovered that the surface reconstruction issue of Cu during CO_2_RRs could be solved by C confinement. Furthermore, this quasi-graphitic C shell enables not only protection but also inner Cu doping by penetration of the *p*-block element for activity improvement. We judiciously controlled C shell formation around Cu catalysts through a self-limiting reaction. Thus, a Cu catalyst is formed within porous, conducting C nanofibers to facilitate ionic and gaseous transport. This can be achieved using a thermodynamically designed gas–solid reaction procedure; during oxygen partial pressure (pO_2_)-controlled calcination, graphitic C forms on Cu by a self-regulated layer-by-layer process, which is promoted by the epitaxial growth of graphene on Cu^29,30^. The thermodynamic equilibrium among CO (g), CO_2_ (g), and C (s) controlled the so-called “Boudouard reaction” for self-passivation of the catalyst. We succeeded in fabricating an ~5-nm-thick quasi-graphitic C shell by a CO-mediated reversible reaction.

The above process produced Cu nanostructures that seamlessly integrated with the support framework, and this structure stabilized the morphology and chemical states of the active materials under vigorous CO_2_-to-hydrocarbon electroconversion. By extensively incorporating N and B into the reconstruction-protected Cu/C platform, the catalytic activity of Cu was tuned to accelerate C_2+_ product formation. We found that N or B atoms could easily penetrate the self-formed quasi-graphitic C layer via the gas phase. Additionally, we achieved a C_2_H_4_ power conversion efficiency of 44% in 1 M KOH as the electrolyte. N modification enhanced the catalytic performance, which showed selectivity and an up to 82% faradaic efficiency (FE) for C_2+_ products with a partial current density over 320 mA/cm^2^ and increased stability. Density functional theory (DFT) calculations elucidated the role of the porous C layer and dopants in the reconstruction-protected Cu/C platform in terms of the adsorption energy of the CO_2_RR intermediates and dimerization barrier. These developments help to guarantee the original strategies of electrocatalysts under actual operating conditions, thereby facilitating elucidation of the fundamental reaction mechanisms and realizing the steady production of hydrocarbons from renewables.

## Results and discussion

### Boudouard reaction for quasi-graphitic C shell formation

To produce Cu hybridized with a C framework system, we utilized electrospinning with a solution containing a polymer (polyacrylonitrile: (C_3_H_3_N)_n_, PAN) and a metal precursor (Cu acetate monohydrate: Cu(CH_3_COO)_2_). Electrospun nanofibers were calcined within a pO_2_-controllable closed chamber system. As the calcination temperature increased, carbonization of the polymer and metal ion reduction occurred, leaving Cu in the carbon fiber matrix.

**Fig. 1:**
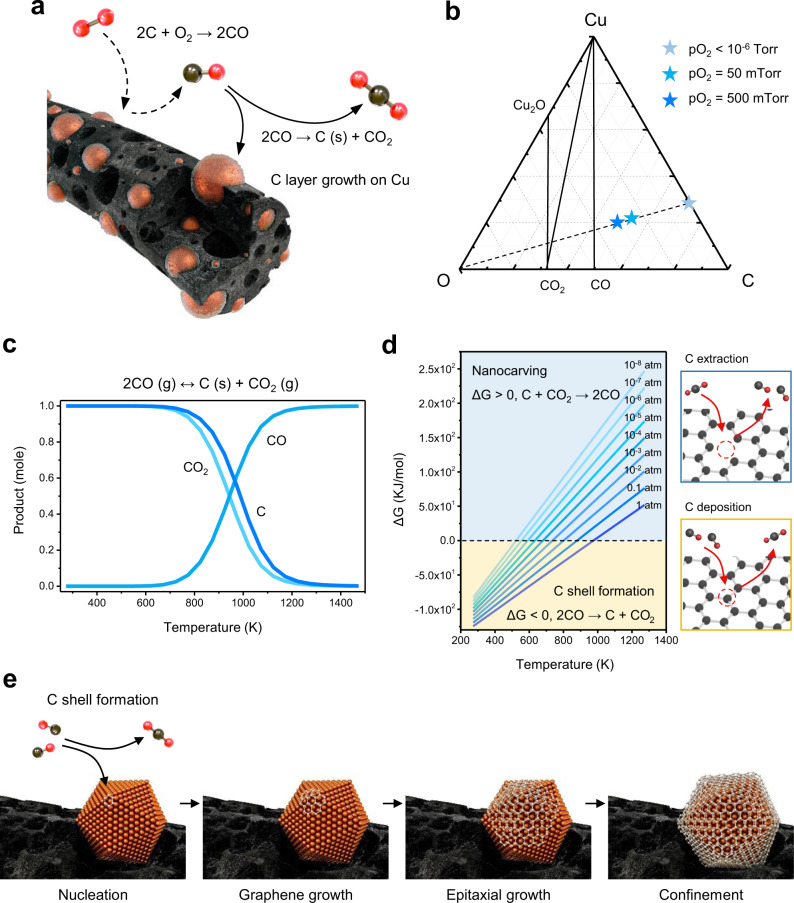
Confinement of Cu nanoparticles by the quasi-graphitic C shell and the thermodynamics based on the Boudouard reaction. **a** Schematic illustration of the CO-mediated reaction in the Cu–C–O system. The C matrix was nanocarved, forming inner pores by C combustion and generating CO-confined Cu nanoparticles in a quasi-graphitic C shell. The dotted line shows nanocarving, and the solid line shows the Boudouard reaction. **b** Ternary phase diagram (Cu–O–C) at 800 °C. Each point indicates the composition conditions representing the pO_2_. It is confirmed that the experimentally conducted conditions are in Cu–CO–C stable regions. **c** Thermodynamically stable product of the reversible Boudouard reaction corresponding to the temperature under 1 atm. **d** Plot showing the Gibbs free energy of the Boudouard reaction vs. temperature under different total pressures from 10^−8^ to 1 atm. The upper (lower) area indicates favorable forward (backward) reaction conditions. The schematic figures on the right describe C extraction and C deposition that reversibly progress depending on the sign of Δ*G*. **e** Formation mechanism of the quasi-graphitic C shell on Cu nanoparticles through the disproportionation of CO.

Controlled C oxidation and reduction was a key design parameter for quasi-graphitic C layer formation on the Cu surface (Fig. 1a), so calcination was performed in a specific thermodynamically predicted region. In the Cu–C–O ternary elemental system, the pO_2_ was controlled between the chemical potentials for the partial oxidation of C and the reduction of Cu because of the intention to form a hybrid structure where metallic Cu particles would combine with the porous C support matrix (Fig. 1b). Because C oxidation (2C (s) + O_2_ (g) → 2CO (s)) is promoted on the metal surface due to catalytic decomposition, the oxidation of C is encouraged more near Cu surfaces than inside the C matrix; thus, the porosity was retained near Cu nanoparticles (the so-called “nanocarving effect”)^31^. The processing parameters for selective C oxidation and reverse C shell formation were predicted using a thermochemical calculation program (Factsage^TM^).

The Boudouard reaction (2CO (g) ↔ C (s) + CO_2_ (g)) provides a theoretical guideline to control the equilibrium among CO, CO_2_, and C (Figs. 1c and S2). At 800 K, the forward reaction was dominant, and the resulting product could be C and CO_2_, but the reverse reaction gradually dominated as the temperature increased. Considering the Gibbs free energy as a function of the pressure and temperature (Fig. 1d), this reaction was induced in one direction whether C was extracted to generate CO or C was deposited to form the shell. The CO-dominant temperature window widened as the pressure decreased. Based on this finding, we speculated that CO was the main gas product during this isothermal time. Due to the disproportionation of CO in the cooling region (2CO (g) → C (s) + CO_2_ (g)), it was solidified back to C (s) on Cu nanoparticles and served as a precursor for the formation of a quasi-graphitic C shell (Fig. S3).

CO, generated via the nanocarving effect, was a key mediator of the formation of the quasi-graphitic C shell on Cu (Fig. 1e). The mechanism of C shell formation was analogous to the mechanism of graphene growth on Cu films. In Cu, the atomically thin C layer grew through surface adsorption, not C precipitation, because of its low C solubility^32^. Gaseous CO dissociated and was then adsorbed on the Cu surface to form graphene nuclei. As CO was continuously supplied, an atomically thin graphene layer grew, and a second layer formed through self-regulated epitaxial growth. It was even possible to control the thickness of the C shell. When CO was continuously removed while maintaining high vacuum, the C shell became much thinner (Fig. S4a). This enabled us to produce a tightly bound composite that actively controls the inherent interaction between Cu and C (Fig. S4).

### Quasi-graphitic C shell study

**Fig. 2:**
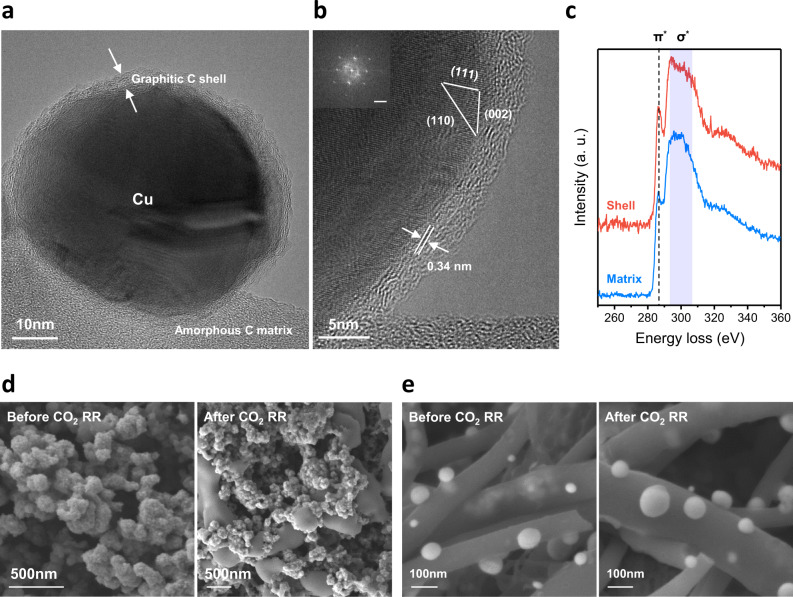
Characterization of the confined Cu nanoparticles and the effect on surface stabilization. **a** High-resolution TEM image of the confined Cu nanoparticles and quasi-graphitic C shell. **b** Atomic lattice of the Cu nanoparticle and C shell. **c** EELS spectra of the amorphous carbon matrix and quasi-graphitic C shell. The peaks under the dotted line and blue area represent π* and σ* bonding, respectively. Surface structure of the (**d**) unconfined and (**e**) confined Cu nanoparticles before and after the CO_2_RR, respectively.

We investigated the microstructure and morphology of the materials by transmission electron microscopy (TEM) and scanning electron microscopy (SEM). Cu nanoparticles coated with a C shell of ~5 nm thickness were seamlessly integrated with the C framework (Fig. 2a and Supplementary Movies 1 and 2). We found that the atomic distance between C layers in the shell was 0.34 nm, consistent with the graphene formed on the surface of the Cu film (Fig. 2b)^29^. TEM electron energy loss spectroscopy (EELS) was conducted for the C matrix and the C shell to analyze the bonding characteristics of C (Fig. 2c). Considering the relative intensity of π^*^ bonding and the peak shape of σ^*^ bonding, the C shell was determined to be graphitic, whereas the C matrix was amorphous^33^. The EELS results verified that the C shell and C matrix were made by distinct formation mechanisms. The amorphous C matrix was formed by the carbonization of PAN, while the quasi-graphitic C shell was formed by graphitic growth on Cu NPs from the gaseous C source.

The effect of the C shell on electrocatalysis was examined by performing control experiments to compare the C-coated Cu NPs to similarly sized Cu nanoparticles without a C shell and with sputtered C layer (Fig. S5). The Cu nanoparticles confined in the C shell initially showed a product distribution similar to that of unconfined Cu, meaning that the C shell with a controlled thickness and bonding characteristics did not deteriorate the CO_2_RR performance. By contrast, the sputtered C layer that had amorphous characteristics, severely degraded the CO_2_RR performance, increasing H_2_ and methane selectivity. (Fig. S5a, b). Interestingly, we found a dramatic increase in stability during the electrochemical CO_2_RR with the quasi-graphitic C shell-protected Cu (Fig. S5c). When the CO_2_RR was operated at −0.67 V (vs. RHE, IR-compensated) in the flow cell, there was a significant advantage in terms of surface structure stabilization achieved with the C shell. In the case of unprotected Cu nanoparticles, severe surface reconstruction occurred after several hours of the reaction at a high reaction rate (Figs. 2d and S6), and the C_2_H_4_ selectivity decreased rapidly. The Cu nanoparticles not only agglomerated to form several micrometer-sized aggregates but also exhibited smoother surfaces. However, in the case of the C shell-coated Cu nanoparticles, they had an intact morphology and chemical state, even after long reaction times (Figs. 2e, S7, and S8). Cu confined by a protective shell exhibited substantially suppressed particle agglomeration and transitions in the oxidized state. The electrochemically active surface area (ECSA) and crystalline size were also preserved (Figs. S9 and S10). To clarify the effect of the C shell on Cu catalysts during the CO_2_RR and reveal the real status, operando X-ray absorption spectroscopy (XAS) analysis was conducted at a current density of 200 mA/cm^2^ (Figs. S11 and S13). The extended X-ray absorption fine structure (EXAFS) peak corresponding to Cu–Cu bonding was prevented from increasing during the reaction, which indicated that surface reconstruction was successfully prevented. The atomic distance and coordination were also calculated (Table S6). We further investigated the effect of the C shell thickness on the chemical and structural states of the Cu catalysts (Figs. S12 and S13 and Tables S5 and S6). Thus, the confined Cu nanoparticles would participate in the CO_2_RR without experiencing significant changes from the initial designed state. Consequently, the confinement of Cu nanoparticles in the quasi-graphitic C shell resulted in high resistance to surface reconstruction under a robust CO_2_RR without performance degradation.

### Incorporation of *p*-block elements in the Cu surface

**Fig. 3:**
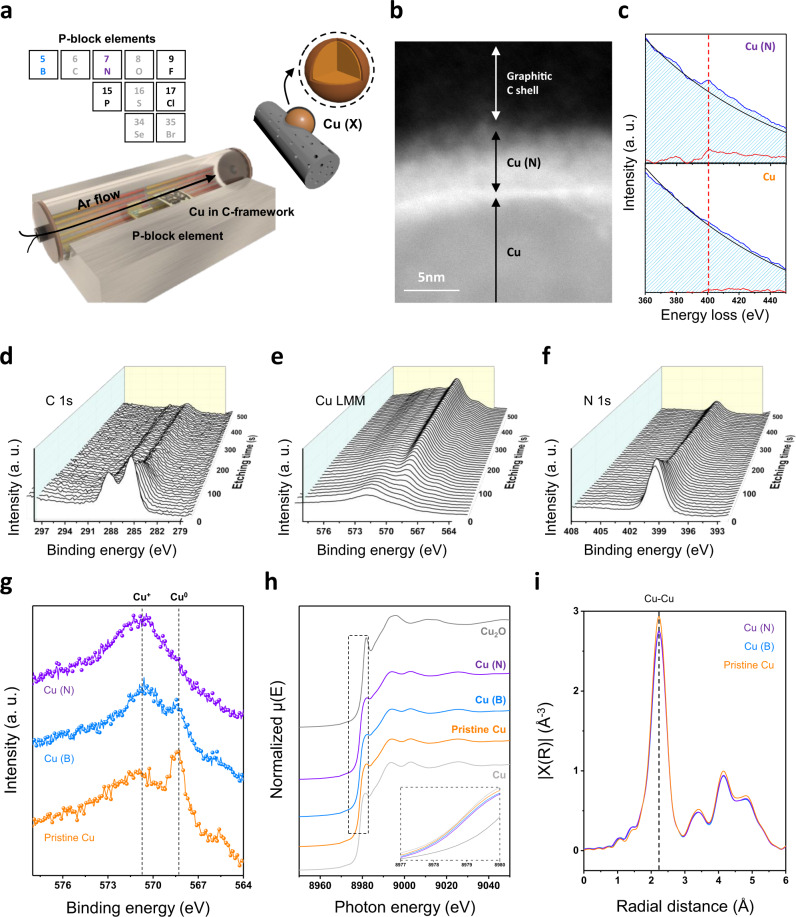
Preparation and characterization of Cu (X) catalysts. **a** Schematic illustration of the fabrication of Cu (X) catalysts. **b** High-resolution TEM image of the Cu (N) film surface. **c** EELS spectra of Cu (N) and the Cu film. **d**–**f** XPS depth profile of the Cu (N) film. Spectra of **d** C *1s*, **e** Cu *LMM*, and **f** N *1s*. Etching was conducted at 10-s intervals with a sputter size of 4 mm^2^. **g**–**i** Chemical state investigation of pristine and *p*-block element-doped Cu. XPS spectra of **g** Cu *LMM*, **h** Cu *K*-edge XANES spectra, and **i** EXAFS spectra obtained for the pristine Cu, Cu (B), and Cu (N) samples.

The binding affinity of the pristine Cu surface in the reconstructed protected Cu/C platform was optimized for hydrocarbon production by incorporating various *p*-block elements (P, F, B, Cl, and N) (Fig. 3a). Doping a *p*-block element, which has a higher electronegativity than Cu, would induce a mixed state of Cu^+^/Cu^0^ analogous to that of Cu_2_O/Cu catalysts^34^. To incorporate *p*-block elements, a secondary gas–solid reaction was applied to the quasi-graphitic C shell-protected Cu. The gas–solid reaction was implemented under inert conditions with a doping source (Fig. S14). The structure of the C shell did not change after doping with *p*-block elements (Fig. S15).

The *p*-block elements reacted with the surface of Cu nanoparticles, which was confirmed by TEM energy dispersive spectroscopy (EDS) mapping (Fig. S16), Raman spectra (Fig. S17), and X-ray photoelectron spectroscopy (XPS) C *1s* spectra (Fig. S18). In the TEM EDS mapping image, each *p*-block element was found to be concentrated on Cu nanoparticles, not significantly impacting the C support matrix. Peaks indicating interactions between C and the foreign elements were not observed. Upon doping N on the Cu surface, the region affected by the incorporated elements was at a depth of ~5 nm, while the core remained metallic (Fig. 3b).

To perform an in-depth study of the chemical status of the electrocatalyst surface and doped element penetration, we prepared a quasi-graphitic C shell on an evaporated Cu film and used it as a model system (Figs. S19–S21). The same C coating and doping strategies were applied to the evaporated film to simplify the model system and analyze the Cu (X) surface (X: doped *p*-block elements). This could be justified because the *p*-block elements are more reactive with Cu than with C. The EELS analysis performed at ~400 eV further confirmed the existence of N on the surface (Fig. 3c). The XPS depth profile of the Cu (N) film further revealed the surface structure of the confined Cu (N) (Figs. 3d–f and S22). The existence of the C shell and penetrating *p*-block elements was verified by the C *1s* and N *1s* orbitals, respectively. Even after etching the C shell of the uppermost layer, N was observed in the top surface of Cu, and it was confirmed that the oxidation state of the Cu surface shifted positively (Cu (B) analysis in Fig. S23). A similar depth profile result was also identified by time-of-flight secondary ion mass spectrometry analysis (Fig. S24).

The phase and electronic structures of quasi-C shell-coated Cu nanoparticle samples were confirmed by performing X-ray diffraction (XRD) and XPS for pristine Cu and each Cu (X). The XRD peaks corresponding to metallic Cu were only observed for the composite, and the peaks did not change after incorporating *p*-block elements (Fig. S26a). In the Cu *LMM* spectra (Figs. 3g and S26b), the peak indicating the metallic Cu (Cu^0^) state was substantially large for pristine Cu. In contrast, the Cu^0^ peak decreased, and the intensity of the peak indicating the Cu^+^ state increased after B and N doping, consistent with metallic core and surface Cu^+^ structure^6^. The XPS spectra corresponding to each dopant verified the existence of *p*-block elements (Fig. S28). The doping concentration in Cu (B) was identified through inductively coupled plasma atomic emission spectroscopy (ICP-AES) (Fig. S29).

To closely investigate the effect of incorporating the *p*-block element into the Cu lattice, the chemical state and coordination were inspected by XANES and EXAFS (Figs. 3h, i and S30). Figure 3h shows the Cu *K*-edge XANES spectra of pristine Cu, Cu (B), and Cu (N). The absorption edges of the Cu samples were similar to those of the Cu reference. The main bonding nature of Cu nanoparticles confirmed by EXAFS was Cu–Cu. We found that there was no significant difference in the atomic distances and coordination numbers for Cu–Cu according to the doped elements (Table S7).

These results indicated that the dopants induced electronic modulation in the Cu lattice and a partial Cu^+^ state without a phase change. Particular characteristics of the quasi-graphitic C shell-coated Cu nanoparticles that could affect CO_2_RR performance, such as morphology (Fig. S15), C crystallinity (Fig. S17), ECSA (Fig. S31), and configuration of N defects (Fig. S32), did not change after doping. These results indicated that the *p*-block element selectively reacted with Cu nanoparticles, and the resulting improvement in catalytic activity could be attributed to the effect of the *p*-block elements on Cu.

### CO_2_RR activity investigation

The CO_2_RR catalytic performances of pristine and Cu (X) composites were evaluated in a flow cell reactor by circulating the catholyte with a peristaltic pump (Fig. S33). The electrolyte was 1 M KOH, and CO_2_ was supplied through a gas diffusion electrode (GDE) with a constant flow rate.

We studied the protective role of the quasi-graphitic C shell by investigating the CO_2_RR performance of conventional Cu nanoparticles after *p*-block doping (Fig. S34). In the Cu nanoparticles without a C shell, it was difficult to enhance the C_2+_ product selectivity with *p*-block doping because severe Cu reconstruction hindered the realization of the doping effect. These results directly reveal the importance of the development of the reconstruction-protected Cu system.

**Fig. 4:**
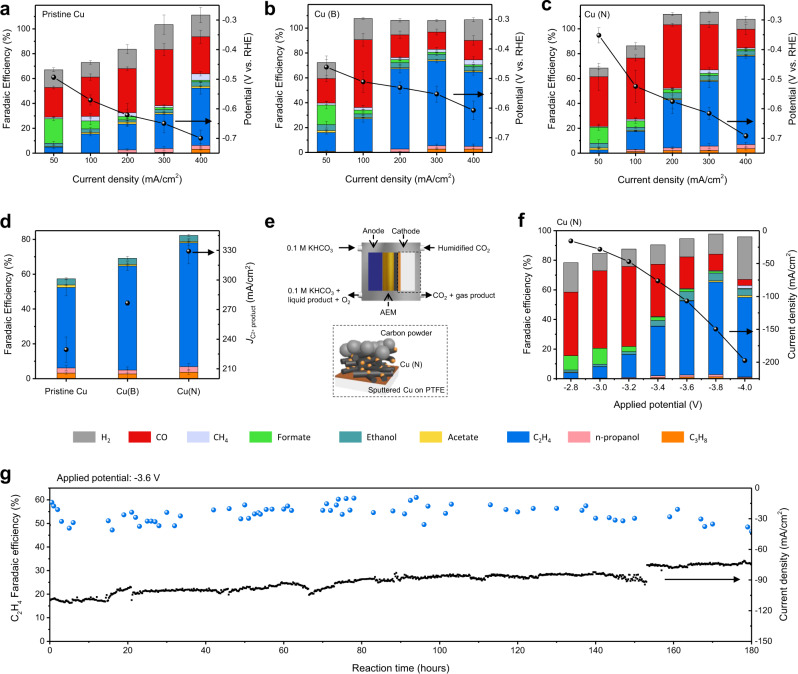
Evaluation of CO_2_RR activity and stability by electrochemical measurements. Selectivity was measured in an alkaline flow cell through the galvanostatic method at *j* values from 50 to 400 mA/cm^2^. CO_2_RR activity comparison among the **a** pristine Cu, **b** Cu (B), and **c** Cu (N) samples in a flow cell with 1 M KOH as the electrolyte. **d** FE and partial current density for C_2+_ products at 400 mA/cm^2^. Error bar indicates the standard deviation of three independent measurements. **e** Schematics of MEA cell operation and the cathode configuration. **f**, **g** CO_2_RR performance of Cu (N) using the MEA cell in 0.1 M KHCO_3_ as the electrolyte. **f** FE and current density at an applied potential from −2.8 to −4.0 V. **g** CO_2_ electroreduction stability at an applied potential of −3.6 V. C_2_H_4_ selectivity and current density is displayed for 180 h of operation.

Regarding pristine Cu (Fig. 4a) with a quasi-graphitic C shell, a substantial amount of formate was produced at a low current density. As the applied potential became more negative, the selectivity of formate decreased, and the CO FE increased. Although the C_2_H_4_ FE increased as the potential increased, a considerable amount of CO was produced over the entire current density range. As the current density increased, the CO FE peaked (45.1%), and then, CO* intermediates started to be converted to C_2_H_4_. The maximum C_2_H_4_ FE and the partial current density for pristine Cu were limited to 46.2% at 400 mA/cm^2^ (−0.70 V vs. RHE). Small amounts of C_3_H_8_, n-propanol, acetic acid, and ethanol were also generated with FE values of 3.2%, 3.0%, 1.4%, and 3.6%, respectively.

Among the explored *p*-block elements, B and N were effective in improving the C_2_H_4_ selectivity. These two elements influenced the selectivity as well as the activity of Cu. In the case of Cu (B) (Fig. 4b), a relatively high proportion of gaseous products was produced at a low current density due to the easy activation of CO_2_. CO was the main product (54.4%) at 100 mA/cm^2^ (−0.51 V vs. RHE) and was then dramatically converted to C_2_H_4_ as the current density increased. Compared with C–C coupling of pristine Cu, C–C coupling of Cu (B) was initiated at a lower overpotential. At −0.55 V vs. RHE, the C_2_H_4_ FE increased to 68.1%. Other hydrocarbons also formed (C_3_H_8_: 2.7%; n-propanol: 2.7%; acetic acid: 1.0%; and ethanol: 4.5%). However, at 400 mA/cm^2^, the C_2+_ selectivity slightly decreased because of the hydrogen evolution reaction (HER).

When incorporating N into the Cu lattice (Fig. 4c), a large amount of CO (39.9%) was generated at 50 mA/cm^2^ (−0.35 V vs. RHE), and the HER was significantly suppressed over the entire current density range. The amount of CO evolved gradually increased, reaching 51.0% at 200 mA/cm^2^ (−0.58 V vs. RHE), and then decreased as it was converted to the C_2+_ product. At the highest current density of 400 mA/cm^2^ (−0.69 V vs. RHE), the maximum FE of C_2_H_4_ reached 71.1%. The amount of other hydrocarbons produced was minute compared to that of C_2_H_4_ (C_3_H_8_: 3.7%; n-propanol: 3.2%; acetic acid: 0.7%; and ethanol: 3.6%). When modified with B and N (Fig. 4d), the Cu (X) composite exhibited enhanced CO_2_-to-hydrocarbon conversion with large reaction rates (C_2+_ partial current densities of 277 and 329 mA/cm^2^, respectively) and improved C_2+_ product selectivity (C_2+_ product FE for Cu (B): 69.2%; C_2+_ product FE for Cu (N): 82.3%).

For long-term CO_2_ electroreduction, Cu (N) was evaluated in 0.1 M KHCO_3_ as the electrolyte using a membrane electrode assembly (MEA) configuration (Fig. 4e–g). Although there was a slight decrease in C_2_H_4_ selectivity due to the change in the reaction environment, such as a change in the electrolyte or the type of electrolyzer (Figs. 4e and S33c), Cu (N) showed product distributions similar to those of the alkaline flow cell as the applied potential increased (Fig. 4f). The C_2_H_4_ FE and C_2+_ product FE reached up to 62.5% and 71.4% at −3.8 V, respectively. The current density increased to −197.4 mA/cm^2^. Cu (N) remained stable over 180 h at an applied potential of −3.6 V (Fig. 4g), one of the best performances ever reported for the neutral electrolyte reaction (Table S10). After the CO_2_RR, a C shell was still present on the Cu nanoparticles (Fig. S7c). The chemical states were still conserved, and dopant leaching was significantly suppressed (Figs. S36 and S37).

### Mechanistic study on enhanced C_2+_ selectivity

**Fig. 5:**
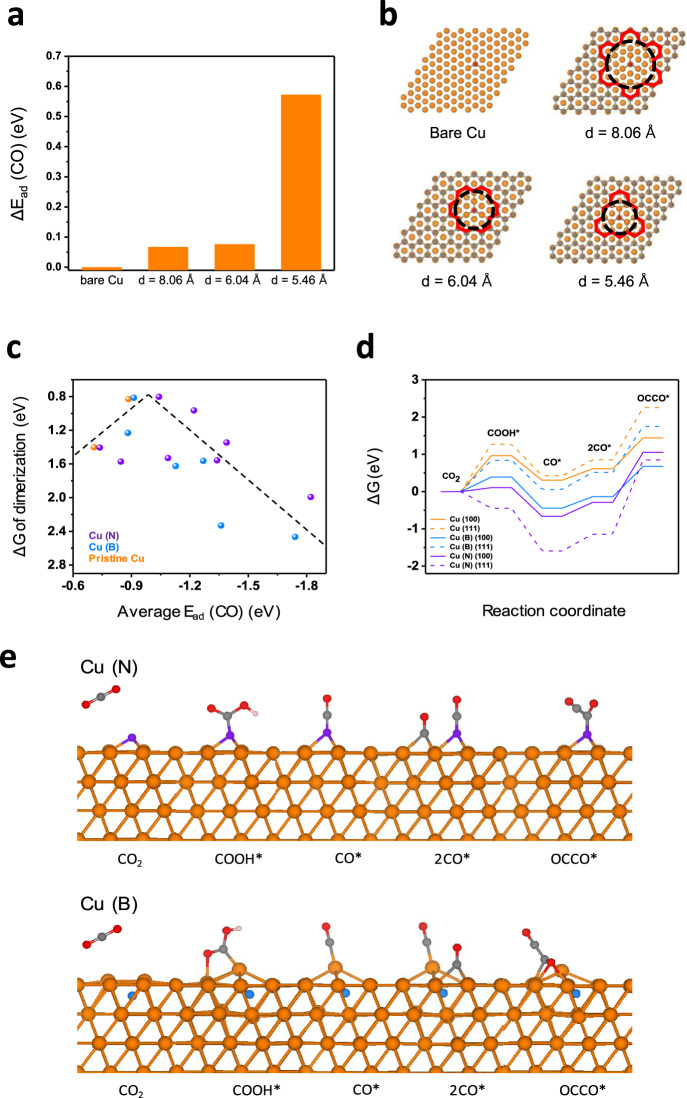
DFT calculations to determine the effect of the C shell and dopant on the catalytic activity of Cu. **a** Difference in the CO adsorption energies (Δ*E*_ad_) of the Cu (111) surface without a C layer and Cu (111) surface encapsulated by a C layer with pore sizes of 8.06, 6.04, and 5.46 Å. **b** Atomic structures for each case. The red line marks a pore in the C layer. The pore size corresponds to the diameter of the black dashed circle. **c** Δ*G* of CO dimerization as a function of the average CO adsorption energy. **d** Free energy diagram for CO dimerization on the pristine Cu, Cu (B), and Cu (N) surfaces. **e** Proposed dimerization mechanism for C_2_H_4_ formation on Cu (N) (111) and Cu (B) (111). Orange, blue, purple, gray, and red spheres represent Cu, B, N, C, and O atoms, respectively.

The experiment confirmed that the C shell encapsulating the Cu nanoparticles plays a significant role in protecting the surface reconstruction of Cu nanoparticles. This protective effect of the C shell was also verified with DFT calculations. The DFT calculation results show that the insertion of the C shell between two Cu slabs increases the binding energy by 1.4 eV. Figure S39 shows the atomic structures resulting from the binding of two Cu slabs with and without C shells. The influence of the C shell on the catalytic activity of the Cu surface was also investigated. We performed DFT calculations to determine the CO adsorption energy on the Cu surface with and without C layers and density of states (Figs. 5a, b and S41). Considering the EELS data obtained for the C layer and the XPS depth profile showing that the gas phase dopant was present on the Cu surface, it could be sufficiently seen that pores exist in the C shell. During the formation of the C shell on the Cu surface, once the pores form in the C shell, the C atoms at the pore edges form very strong bonds with Cu surfaces, so it is difficult to form C–C bonds in the C shell and fill the pores. From our DFT calculations, the formation of a bond between the Cu surfaces and the carbon atoms at the pore edge decreases the total energy by ~2.4 eV compared to the formation of a bond among C atoms at pore edges. Hence, the pore structure becomes stable due to bond formation between the Cu surfaces and the C atoms at the pore edge. The atomic structures of both cases are carefully explained in Fig. S40. The difference in the CO adsorption energies (Δ*E*_ad_) compared with those of the Cu surface without a C layer and the atomic structures of the CO-adsorbed Cu surface without a C layer and with a C layer having pore sizes of 8.06, 6.04, and 5.46 Å are presented in Fig. 5a, b. Δ*E*_ad_ is 0.6 eV for the Cu surface covered by a C layer with a pore of size 5.46 Å compared to the Cu surface without a C layer. However, for a slightly larger pore size of 6.04 Å, Δ*E*_ad_ decreases abruptly to 0.08 eV. For a pore size of 8.06 Å, Δ*E*_ad_ also decreases to 0.07 eV. As the pore size increases, the CO adsorption energy becomes close to that of the Cu surface without a C layer. The abrupt decrease in Δ*E*_ad_ for the pore size larger than 6 Å is attributed to the bonding character between Cu atoms and C atoms at the pore edge. In the bonding between a CO molecule and Cu surfaces, the C atom of the CO molecule typically forms bonds with three Cu atoms. When the pore size is 5.46 Å, those Cu atoms also form bonds with not only the C atom of the CO molecule but also the C atoms of the C layer at the pore edge. However, as the pore size increases to greater than 6 Å, there exist Cu surface atoms that do not form bonds with the C atoms of the C shell at the pore edge. As the pore size increases, the CO adsorption energy could increase because there exist Cu atoms that form bonds with only CO molecules. Therefore, Δ*E*_ad_ decreases abruptly as the pore size increases over 6 Å, and the CO adsorption energy becomes similar to that on the Cu surface without a C layer. The impact of the C layer on the electronic property of the Cu surface was also studied by calculating the projected density of states (PDOS) of the 3d orbitals of Cu (Cu_3d_) and 2p orbitals of C (C_2p_). Fig. S41 shows PDOS plots of Cu_3d_ and C_2p_ for the case shown in Fig. 5b. The PDOS plots indicated a trend similar to the CO adsorption energy trend. The larger the pore size of the C layer was, the more similar the PDOS plot was to that of bare Cu. In particular, the PDOS for *E* − *E*_f_ = −7 eV showed significant differences depending on the pore size of the C layer. In Fig. S41d, the PDOS for *E* − *E*_f_ = −7 eV was lower than that for any other case, which means that CO is weakly bound on the Cu surface encapsulated by C layers. From the DFT calculation results, we confirmed that the pore size of the C layer can influence the electronic structure of Cu and the interaction between Cu and CO molecules.

The effect of doping with *p*-block elements on the C_2_H_4_ selectivity of Cu was also studied by DFT calculations. Because the C layer with pores larger than 6 Å does not influence the catalytic activity of the Cu surface, DFT calculations were conducted using the Cu surface model without a C layer to study the doping effect. The effect of *p*-block element doping was studied using a Cu surface doped with several *p*-block elements: B, N, P, F, and Cl. As shown in the plots of the average CO adsorption energy in Fig. S42, B and N have significant impacts on the catalytic activity of Cu. To elucidate the increased C_2_H_4_ selectivity of B-doped and N-doped Cu, the average adsorption energy of two CO molecules and the free energy changes of the C–C coupling reaction for pristine Cu, Cu (B), and Cu (N) were calculated. Since the dimerization of two CO* on the Cu surface to form OCCO* is the rate-determining step in the C_2_H_4_ formation pathway^35–39^, the free energy change of CO* dimerization was used as the descriptor of C_2_H_4_ productivity. Figure 5c shows the plot of CO* dimerization energy as a function of the average CO adsorption energy of pristine Cu, Cu (B), and Cu (N). The data were obtained by calculating the CO adsorption and CO* dimerization energies using different facets (Cu (100) and Cu (111)), doping sites, and doping concentrations. The volcano plot shows the relationship between the average CO adsorption energy and CO* dimerization energy. The CO* dimerization energy decreased as the absolute value of the average CO adsorption energy increased to ~1.0 eV. Then, the CO* dimerization energy increased as the absolute value of the average CO adsorption energy increased further. The point closer to the top of the volcano has a low CO* dimerization energy. The catalyst corresponding to the point closer to the top of the volcano produced a large amount of C_2_H_4_. In Fig. 5c, Cu (B) and Cu (N) have data points closer to the top of the volcano than pristine Cu. This means that the average CO adsorption energy of Cu can be shifted to the optimal value by B and N doping, and the CO* dimerization energy can be lowered, resulting in a higher selectivity for C_2_H_4_.

To investigate the additional effect of B and N doping on the CO_2_RR forming C_2_H_4_ on the Cu surface, the free energy change in the reaction pathway of the CO_2_RR was calculated. Figure 5d shows the free energy diagram of the CO_2_RR for CO and CO* dimerization. Free energy calculations were performed using pristine Cu, Cu (B), and Cu (N) with different Cu facets (Cu (100) and Cu (111)). Schematic diagrams of the CO_2_RR pathways achieving CO and CO* dimerization on Cu (B) (111) and Cu (N) (111) are presented in Fig. 5e for six surfaces. The doping sites for dopants and the binding sites for adsorbed species are based on the most energetically stable structures. Schematic diagrams of the reaction pathway for other surfaces are shown in Fig. S43. The barrier of the CO_2_RR for CO production on Cu (B) or Cu (N) is lower than that on pristine Cu. This is because COOH* and CO* are more stable on Cu (B) and Cu (N) than on pristine Cu. Since Cu (B) and Cu (N) have higher CO adsorption energies than pristine Cu, the CO_2_RR for CO production is facilitated by B and N doping. Thus, doping leads to higher CO* coverage, and it was reported that increased CO* coverage improves CO* dimerization by lowering the energy barrier of CO* dimerization^40,41^. Therefore, doping Cu with B and N can promote the CO_2_RR to form CO, which leads to higher selectivity for C_2_H_4_.

The fact that the porous C layer did not interrupted the CO_2_ electrocatalysis of Cu was also confirmed in the Cu (B) and Cu (N) cases (Fig. S44). The CO adsorption energies of the Cu (B) and Cu (N) surfaces encapsulated with porous C layers were similar to the CO adsorption energy of the Cu surface without a C layer. In addition, from the depth profile results showing that the dopant existed in not only the Cu surface but also the C layer, we investigated the effect of doping B and N into a graphene layer (Fig. S45). Because it was reported that doping N in a graphene layer lowers the barrier energy for CO_2_ reduction to CO^42^, the same effect was expected in our system. The barrier of the CO_2_RR to form CO was reduced by 1.3 eV relative to that of pristine graphene doped with B and N atoms. CO binding on the B and N sites of the C layer was relatively unstable compared to CO binding on the Cu surface. This meant that when CO_2_ is reduced to CO on a B- and N-doped C layer, CO can diffuse to the Cu surface. Therefore, the B- and N-doped C layer can contribute to the CO_2_RR by reducing CO_2_ to CO and supplying CO to the Cu surface, resulting in high CO* coverage.

In conclusion, we have developed a surface stabilization method by forming a quasi-graphitic C shell on Cu nanoparticles for selective and steady CO_2_ electroreduction to C_2_H_4_. Controlling the reversible CO-mediated reactions based on thermodynamics, Cu nanoparticles were coated with a multidimensionally modified C support. The electrocatalyst system confined by the C shell was structurally and chemically stabilized, so the rationally designed precatalysts were directly applied to the vigorous CO_2_RR as they were. To enhance C_2+_ productivity, the binding affinities with the reaction intermediate were further adjusted by the incorporation of *p*-block elements. The initial Cu^+^/Cu^0^ mixed state induced by B and N doping improved C_2+_ productivity, and we revealed the amplifying effect of B and N doping through the combination of experiments and DFT calculations. This finding suggests ways to achieve surface-stabilized catalysts that retain their original states without modification while enabling further improvement by modifying the binding of intermediates for vigorous multicarbon synthesis via the CO_2_RR.

## Methods

### Materials preparation

A homogenous solution was prepared by dissolving 3.0 and 3.6 g of PAN (*M*_w_ = 150,000 g/mol, Sigma-Aldrich) and Cu acetate monohydrate (*M*_w_ = 199.65 g/mol, Sigma-Aldrich), respectively, in 40 g of dimethylformamide (C_3_H_7_NO, Sigma-Aldrich). The solution was injected into a syringe pump (KDS 100, KD Scientific) and ejected at a constant rate of 0.3 ml/h and an applied voltage of 15 kV. The distance between the needle tip and the collector was 15 cm. Through electrospinning, a nanostructure composed of C and copper precursors was produced. The as-spun nanofiber was gathered from the collector, and 200 mg was used at a time. A rotary pump and turbo-molecular pump were utilized to remove oxygen in the tube. The desired amount of oxygen was injected into the tube with a mass flow controller. The chamber was sealed so that there was no gas exchange with the outside, and then, the sample was calcined at 800 °C for 5 h with a ramp rate of 3 °C/m.

RTA equipment (KVR-2000, Korea Vacuum Tech.) was used to incorporate the *p*-block element on the Cu surface. The doping source and Cu composite were placed together and heated at 500 °C under flowing Ar (Fig. S14b). Red phosphorus (P), ammonium fluoride (NH_4_F), boric acid (H_3_BO_3_), magnesium chloride (MgCl_2_), and urea (CH_4_N_2_O) were used as the doping sources for P, F, B, Cl, and N, respectively. The Cu film was then deposited on a Si wafer at a rate of 8 Å/s using a thermal evaporator (Vacuum Science Laboratory) (Figs. S19–S24).

### Thermodynamic calculations

All thermodynamic calculations were conducted using a thermochemical database program (Factsage^TM^ software) based on the Fact Pure Substance database. To predict the reaction products, the quantity of solid reactant and the amount of oxygen in the experiment were considered. Detailed values of each parameter are available in the Supplementary Information (Tables S1–S4).

### Material characterization

The microstructure of the catalysts was investigated by field emission-SEM (Sigma, Carl Zeiss), HR-TEM (JEM-2100F (JEOL Ltd.), Tecnai F20 (FEI)), and Cs-corrected monochromated TEM (Themis Z (Thermo Fisher), JEM-ARM200F (JEOL Ltd.)). The phase and chemical state were analyzed using XRD (New D8 Advance, Bruker) and XPS (PHI 500 VersaProbe^TM^, ULVAC-PHI). The XPS spectra were calibrated using the C *1s* peak. The doping concentration was investigated by ICP-AES (Optima 8300, Perkin-Elmer). The bonding characteristics of C were confirmed by Raman spectroscopy (LabRAM HV Evolution, HORIBA). The oxidation state and atomic configuration of pristine Cu and Cu (X) were examined by XAS at the Cu *K*-edge. This analysis was performed using the 8C-Nano XAFS and 10C-Wide XAFS line at the Pohang light source (PLS-ΙΙ). Data fitting was performed with the ATHENA and ARTEMIS programs.

### Electrocatalytic reduction of CO_2_

A homogenous ink was prepared by dispersing 40 mg of the Cu catalyst through sonication in a solution containing 9 ml of isopropyl alcohol (IPA, Daejoong Chem.) and 200 µl of a Nafion solution (5 wt%, Sigma-Aldrich). GDEs were fabricated by air spraying the dispersed ink on a gas diffusion layer (GDL-39BC, SIGRACET). The loading amount was fixed at 0.3 mg/cm^2^. For stable electroreduction, the cathode was also prepared on a PTFE substrate (0.45 μm). A Cu film (150 nm) was then sputtered on the substrate to improve electronic conduction. Next, the Cu electrocatalysts and C nanopowders were sprayed as previously reported^7^. The anode was IrO_2_ deposited on a Ti frit (0.25 mm thickness, 2GDL6N-025) by the dip-coating method. IrCl_3_ ∙ H_2_O (30 mg, Sigma-Aldrich) was dissolved in 10 ml of IPA with 200 μl of HCl for dip-coating. Then, the thermal decomposition of the Ir precursor was conducted at 500 °C for 30 min. The dipping and thermal decomposition processes were repeated ten times. The CO_2_RR catalytic activity was examined in a laboratory-made flow cell reactor and MEA (Fig. S33). The flow cell configuration consisted of three electrodes, of which Hg/HgO was used as the reference electrode and Pt foil was used as the counter electrode. During the electrocatalytic reaction, the anolyte and catholyte were separated by an anion exchange membrane (Sustanion X37-50, Dioxide Materials), and the catholyte was introduced at a rate of 0.85 ml/min using a peristaltic pump. CO_2_ was supplied at a constant rate of 50 sccm. The potential of the Hg/HgO electrode was converted to RHE using the following equation: *E* (RHE) = *E* (Hg/HgO) + 0.14 + 0.0592 × pH. The solution resistance was measured by electrochemical impedance spectroscopy before and after the reaction. IR compensation was performed with this value, and the equation is as follows: *E*_comp._ = *E*_app._ + 0.85 × IR_u_. The gaseous reaction products were directly connected to a gas chromatography instrument (INFICON Inc.) in-line to continuously investigate the product selectivity and activity for 1 h. The flow rate of the product gas was measured by a mass flow meter. The liquid products were collected and investigated by a high-resolution nuclear magnetic resonance spectrometer (600 MHz, Bruker). Dimethyl sulfoxide was added to the product solution as an internal standard. The FE of each product was calculated by the equation below:

$${\rm{FE}}( \% )=\frac{z\cdot n\cdot F}{Q}$$

*z* the number of electrons exchanged, *n* the number of moles of each product, *F* 96,485 C/mol, and *Q* input charge.

### DFT calculations

DFT calculations were performed within the generalized gradient approximation of the Perdew–Burke–Ernzerhof functional^43^ using the Vienna ab initio simulation package^44,45^. The plane-wave basis had an energy cutoff of 400 eV. The Brillouin zone was sampled using a 3 × 3 × 1 Monkhorst-Pack mesh. Structural optimization was performed until the force on each atom was less than 0.01 eV/Å. A 3 × 3 four-layer slab of Cu (100) and a 4 × 4 four-layer slab of Cu (111) were modeled, and the vacuum space was set to 15 Å to avoid interactions with their periodic images. For electrochemical reactions involving the proton-coupled electron transfer step, the reaction free energies were calculated based on the computational hydrogen electrode model^46,47^.

## Supplementary information

Supplementary information

Supplementary Movie 1

Supplementary Movie 2

## Data Availability

The data that support the findings of this study are available from the corresponding author on reasonable request.
