# Supplementary material for: Quasi-graphitic carbon shell-induced Cu confinement promotes electrocatalytic CO2 reduction toward C2+ products
Source: Nat Commun. 2021 Jun 21;12:3765. doi: 10.1038/s41467-021-24105-9 (PMC8217160; doi:10.1038/s41467-021-24105-9)
Supplement: Supplementary file 1 — Supplementary information [file 41467_2021_24105_MOESM1_ESM.pdf]

Supplementary Information

Quasi-graphitic carbon shell-induced Cu confinement promotes electrocatalytic CO_2 reduction toward C_{2+} products

Ji-Yong Kim[1], Deokgi Hong[1], Jae-Chan Lee[1], Hyoung Gyun Kim[1], Sungwoo Lee[1], Sangyong Shin[2], Beomil Kim[3], Hyunjoo Lee[2], Miyoung Kim[1], Jihun Oh[3], Gun-Do Lee[1,4,*], Dae-Hyun Nam[5,*] and Young-Chang Joo[1,4,6,*]

[1] Department of Materials Science & Engineering, Seoul National University, Seoul 08826, Republic of Korea

[2] Department of Chemical and Biomolecular Engineering, Korea Advanced Institute of Science and Technology, Daejeon 34141, Republic of Korea

[3] Department of Materials Science and Engineering, Korea Advanced Institute of Science and Technology, Daejeon 34141, Republic of Korea

[4]Research Institute of Advanced Materials (RIAM), Seoul National University, Seoul 08826, Republic of Korea

[5]Department of Energy Science & Engineering, Daegu Gyeongbuk Institute of Science & Technology (DGIST), Daegu 42988, Republic of Korea

[6]Advanced Institute of Convergence Technology, 145 Gwanggyo-ro, Yeongtong-gu, Suwon 16229, Republic of Korea

*Corresponding authors:

Gun-Do Lee*

E-mail: gdlee@snu.ac.kr Tel: +82-880-5898

[1]1, Gwanak-ro, Gwanak-gu, Seoul 08826, Republic of Korea

Dae-Hyun Nam*

E-mail: dhnam@dgist.ac.kr Tel: +82-53-785-6426, Fax: +82-53-785-6409

[5]333, Techno jungang-daero, Hyeonpung-eup, Dalseong-gun, Daegu 42988, Republic of Korea

Young-Chang Joo*

E-mail: ycjoo@snu.ac.kr Tel: +82-2-880-8986, Fax: +82-2-883-8197

[1]1, Gwanak-ro, Gwanak-gu, Seoul 08826, Republic of Korea

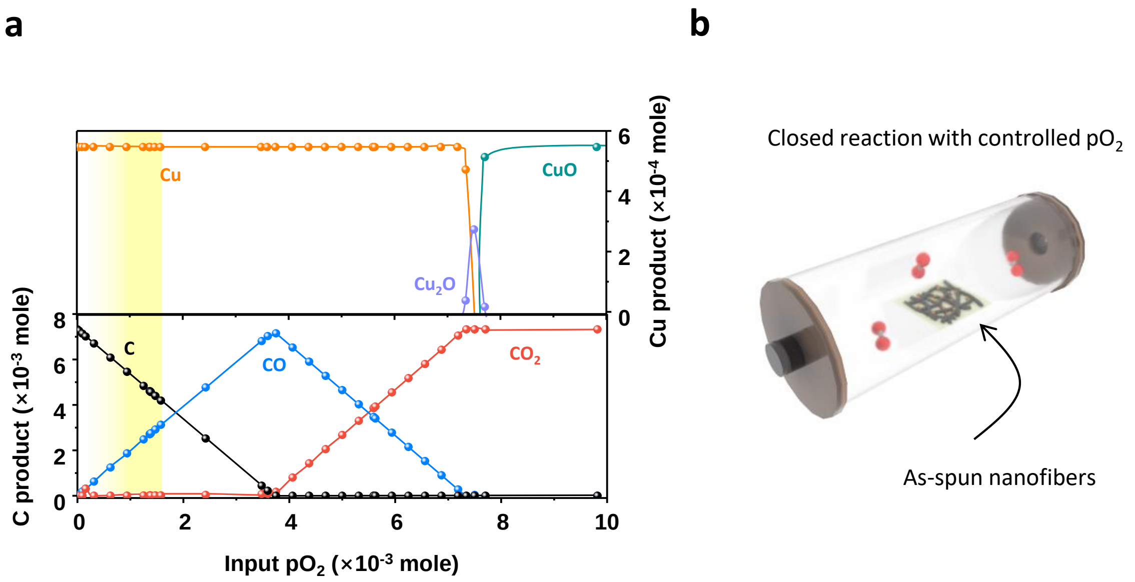

Fig. S1 | Thermodynamic calculations on Cu confinement. a, thermodynamically predicted reaction product after the calcination at 800°C. The products were described as a function of pO_2. The yellow region indicates experimentally controlled pO_2 region. **b**, schematic illustration of the fabrication process. Reaction was occurred in the closed system with O_2, resulting in CO and CO_2.

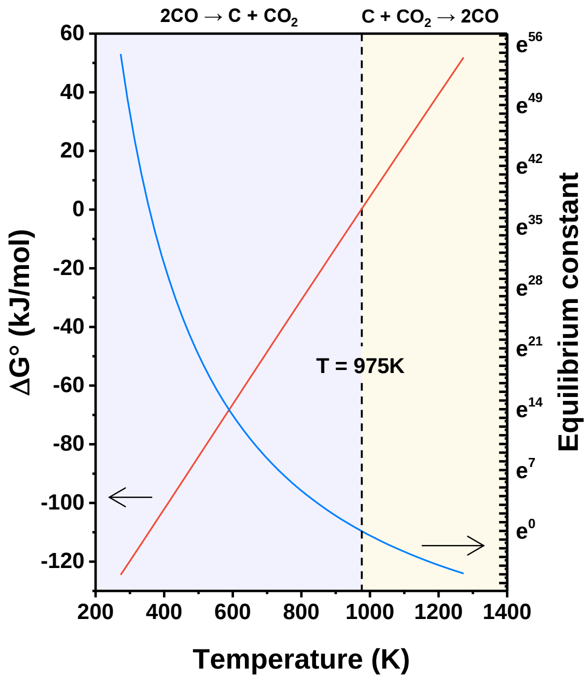

Fig. S2 | Gibbs free energy and equilibrium constant of Boudouard reaction under 1 atm versus temperature. The red and blue line shows the Gibbs free energy, and equilibrium constant calculated accordingly.

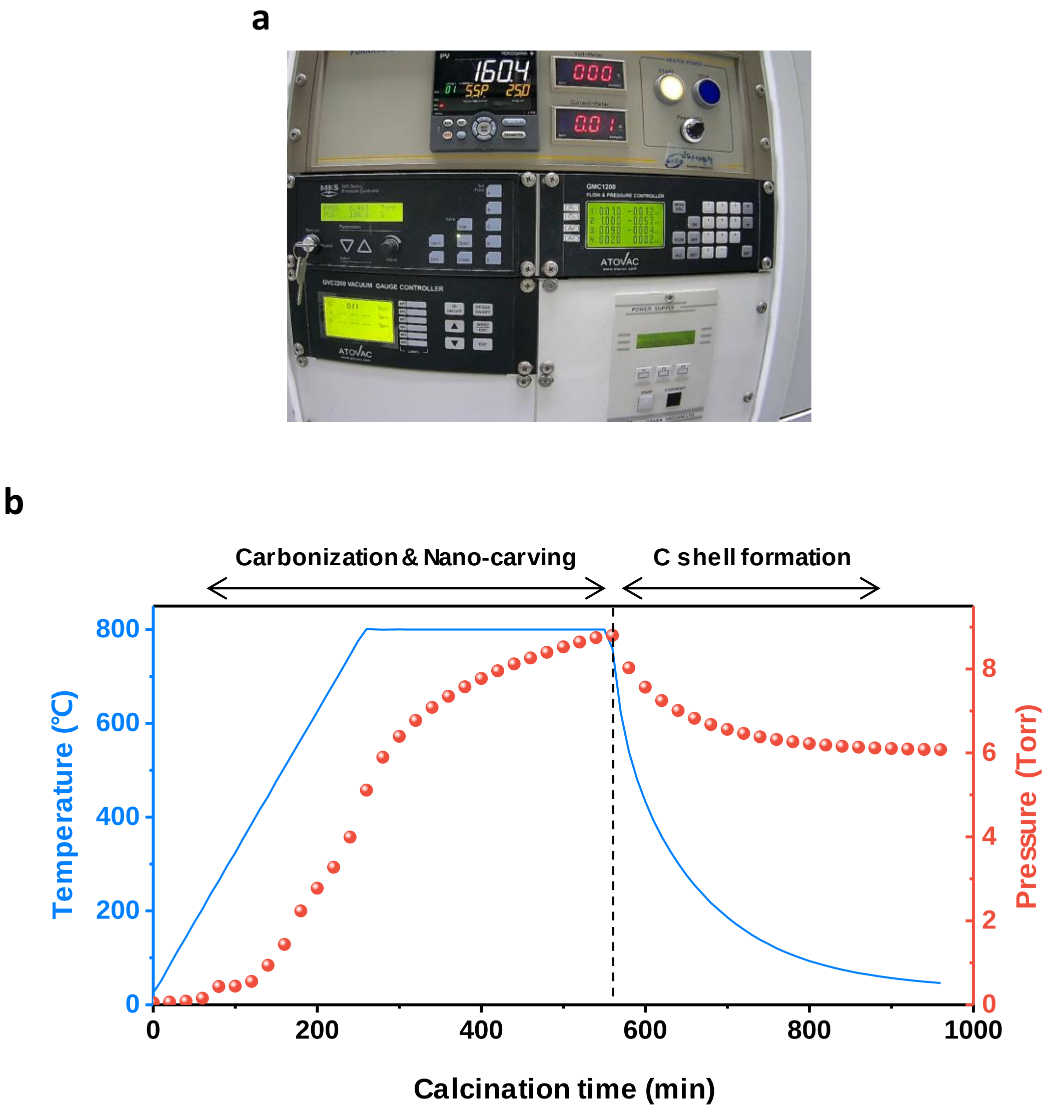

Fig. S3 | Pressure profile during the pO_2-controlled calcination. a, the image of equipment that check the reaction temperature and working pressure. **b,** temperature and pressure profile during the calcination. It is speculated that carbonization and nano-carving were progressed while the pressure was increased, and C shell was formed while the pressure was decreased.

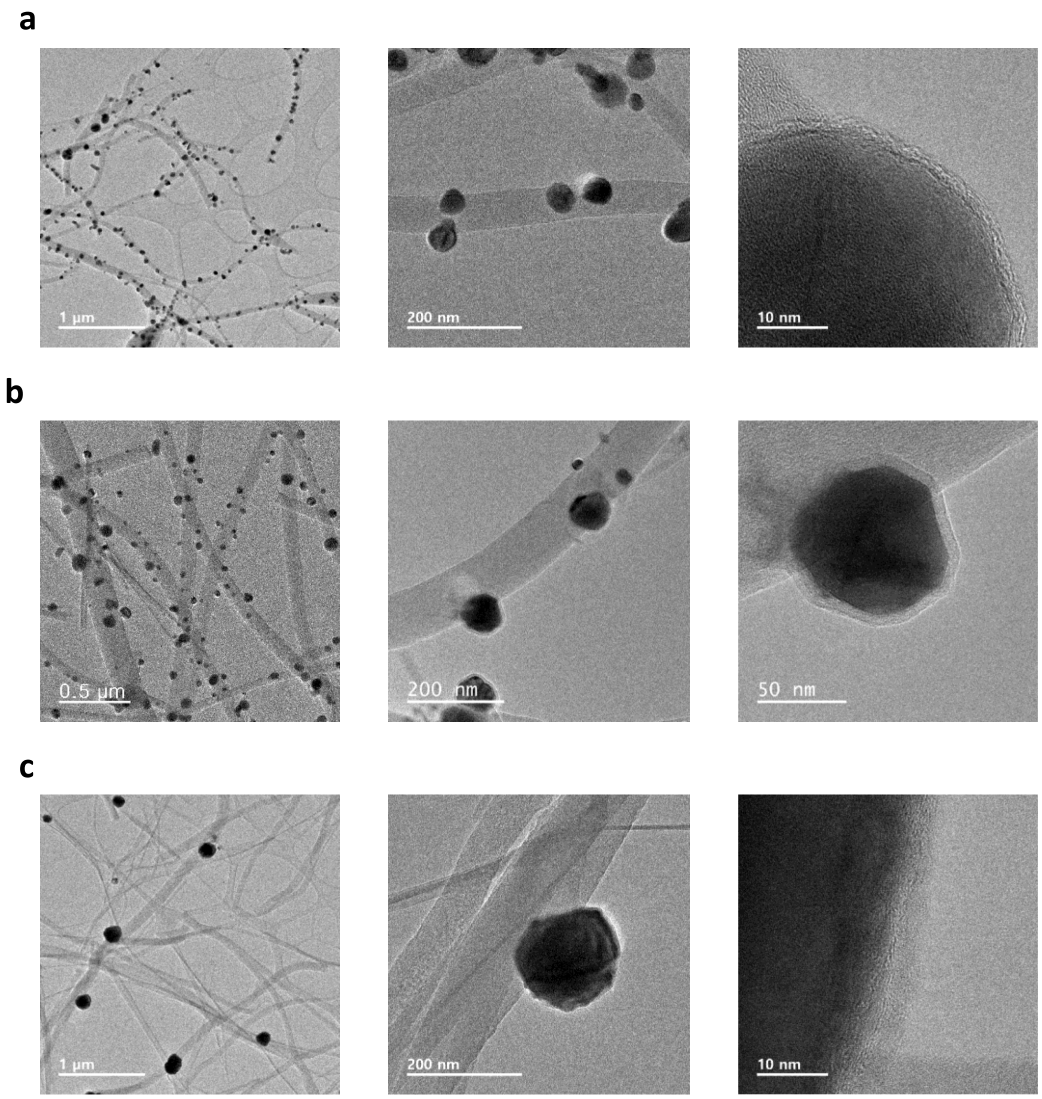

Fig. S4 | Microstructure of confined Cu nanoparticles after pO_2-controlled calcinations. HR-TEM image of confined Cu nanoparticles after the calcination under (**a**) high vacuum condition, (**b**) pO_2 = 50 mTorr, and (**c**) 500 mTorr.

By controlling the degree of C oxidation, active-support interaction was controlled in terms of size, porosity, spatial distribution, and shell thickness. When the porosity in the supporting

matrix increased, the mobility of Cu crystallite might increase. As a result, the size of nanoparticles increased and outward diffusion was promoted.

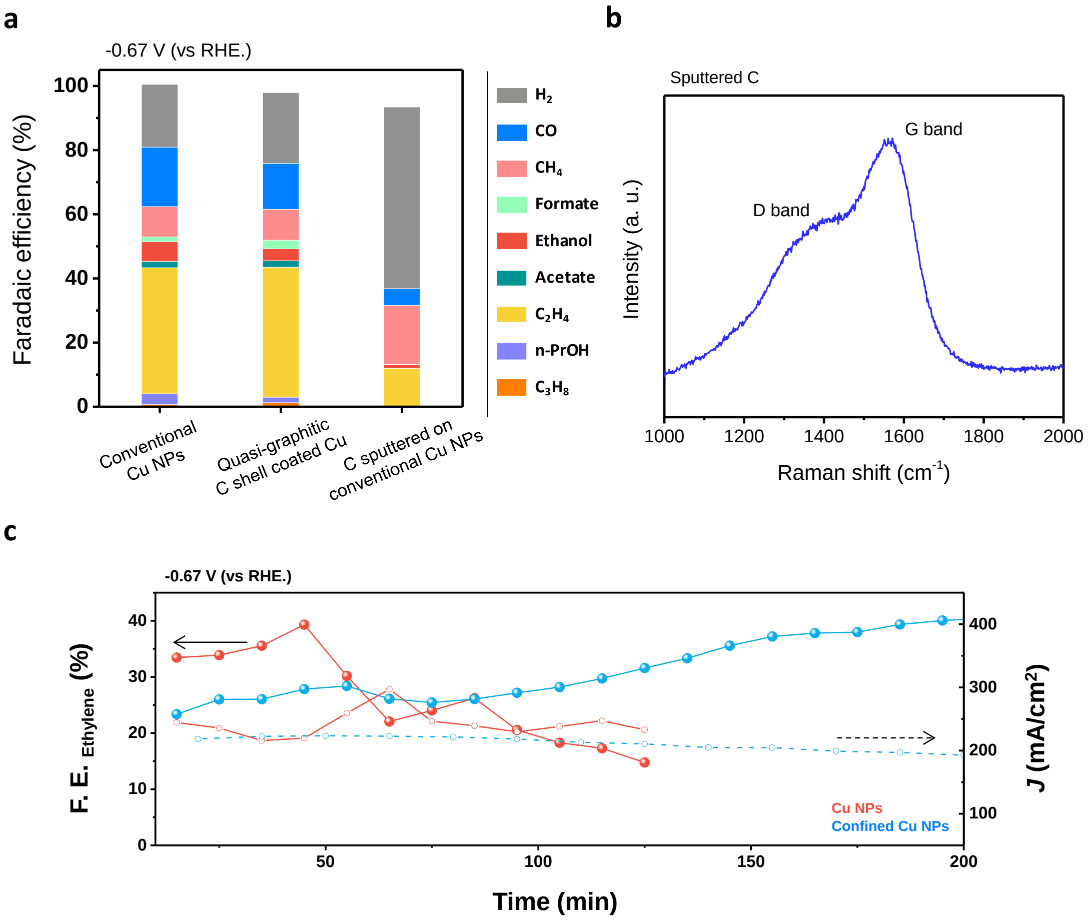

Fig. S5 | Effect of C shell confinement on CO_2 RR activity and stability. **a,** Product selectivity comparison between conventional Cu nanoparticles, Cu nanoparticles coated with a quasi-graphitic C shell, and C sputtered on the conventional Cu nanoparticles. C layer was fabricated by DC sputtering[2]. The working power and pressure were 300 W and 50 mTorr, respectively. **b,** Raman spectra of sputtered C layer. This indicated *sp*^{2}-bonding-rich amorphous characteristics[2]. **c,** Stability comparison between conventional Cu nanoparticles and Cu nanoparticles confined by a C shell. The reaction proceeded at -0.67 V (vs RHE.). The electrode was fabricated on commercial carbon paper. With respect to selectivity, the conventional Cu nanoparticles and Cu nanoparticles confined by a C shell initially exhibited little difference.

Over reaction time, the performance of Cu nanoparticles deteriorated. By contrast, confined Cu showed excellent stability.

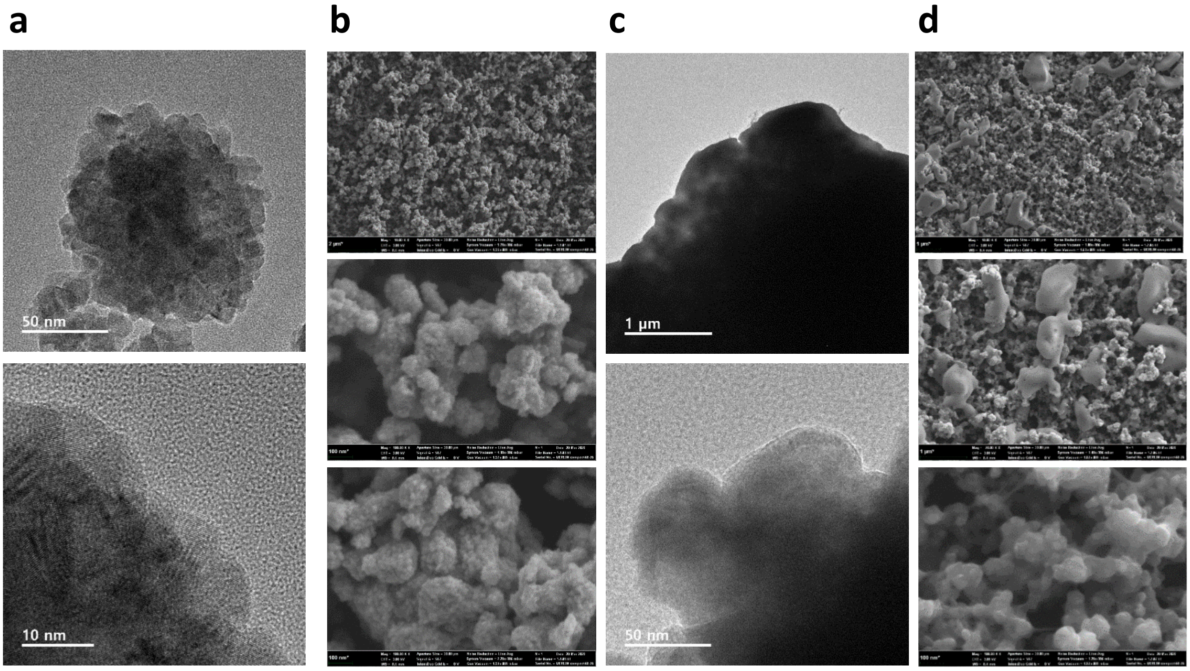

Fig. S6 | Morphology transition of unconfined Cu nanoparticles. Microstructure investigated by (**a, c**) TEM and (**b, d**) SEM of unconfined Cu nanoparticles (a, b) before and (c, d) after CO_2RR. The CO_2RR was conducted under constant j = 200 mA/cm^2 for 1 hour 30 minutes.

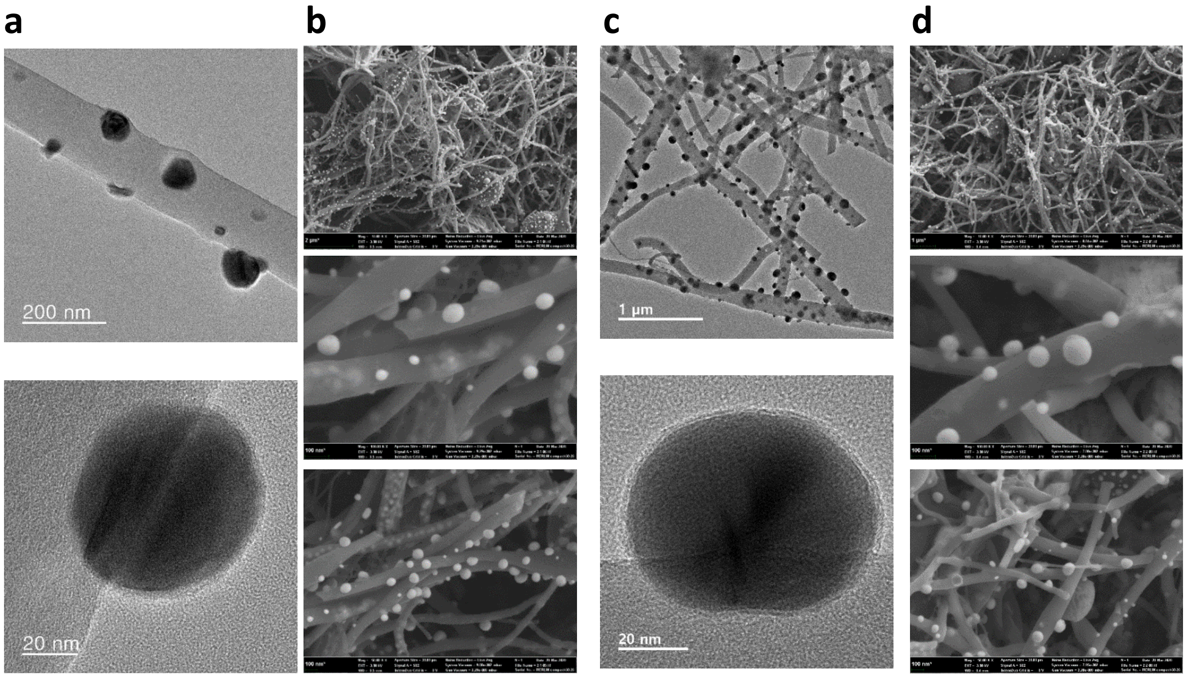

Fig. S7 | Morphology transition of confined Cu nanoparticles. Microstructure investigated by (**a, c**) TEM and (**b, d**) SEM of Cu nanoparticles confined by quasi-graphitic C shell (**a, b**) before and (**c, d**) after CO_2RR. The CO_2RR was conducted under constant j = 200 mA/cm^2 for 5 hours.

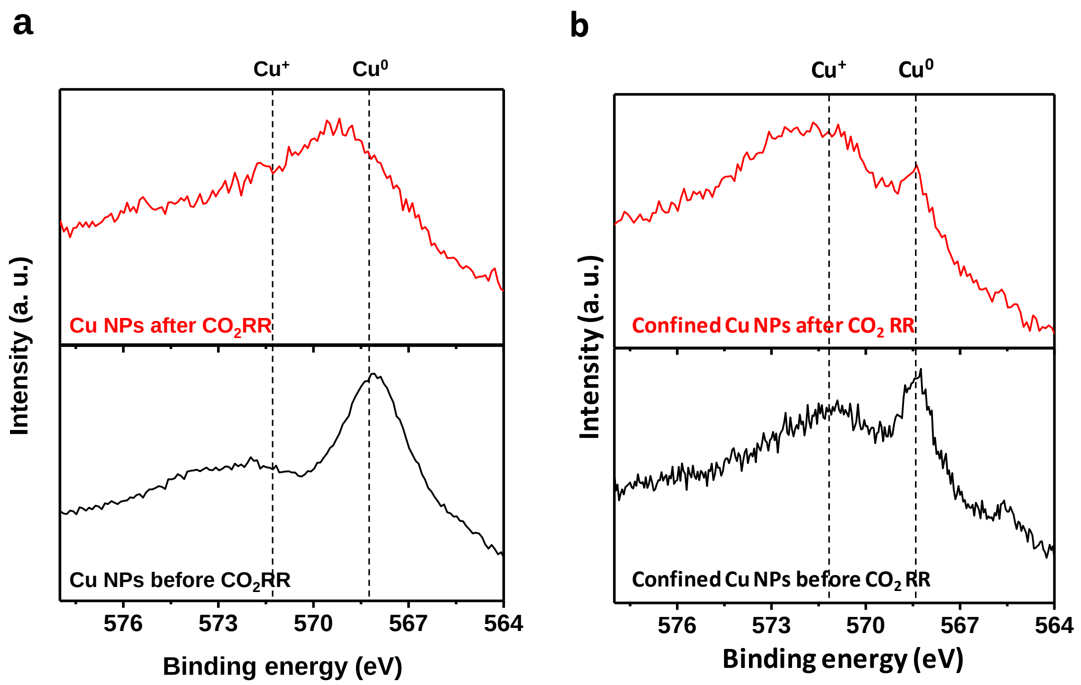

Fig. S8 | Investigation on oxidation state transition. XPS Cu *LMM* spectra of (**a**) unconfined Cu nanoparticles and (**b**) confined Cu nanoparticles before and after CO_2 RR. The CO_2 RR was conducted under constant j = 200 mA/cm^2 for 1.5 and 5 hours, respectively.

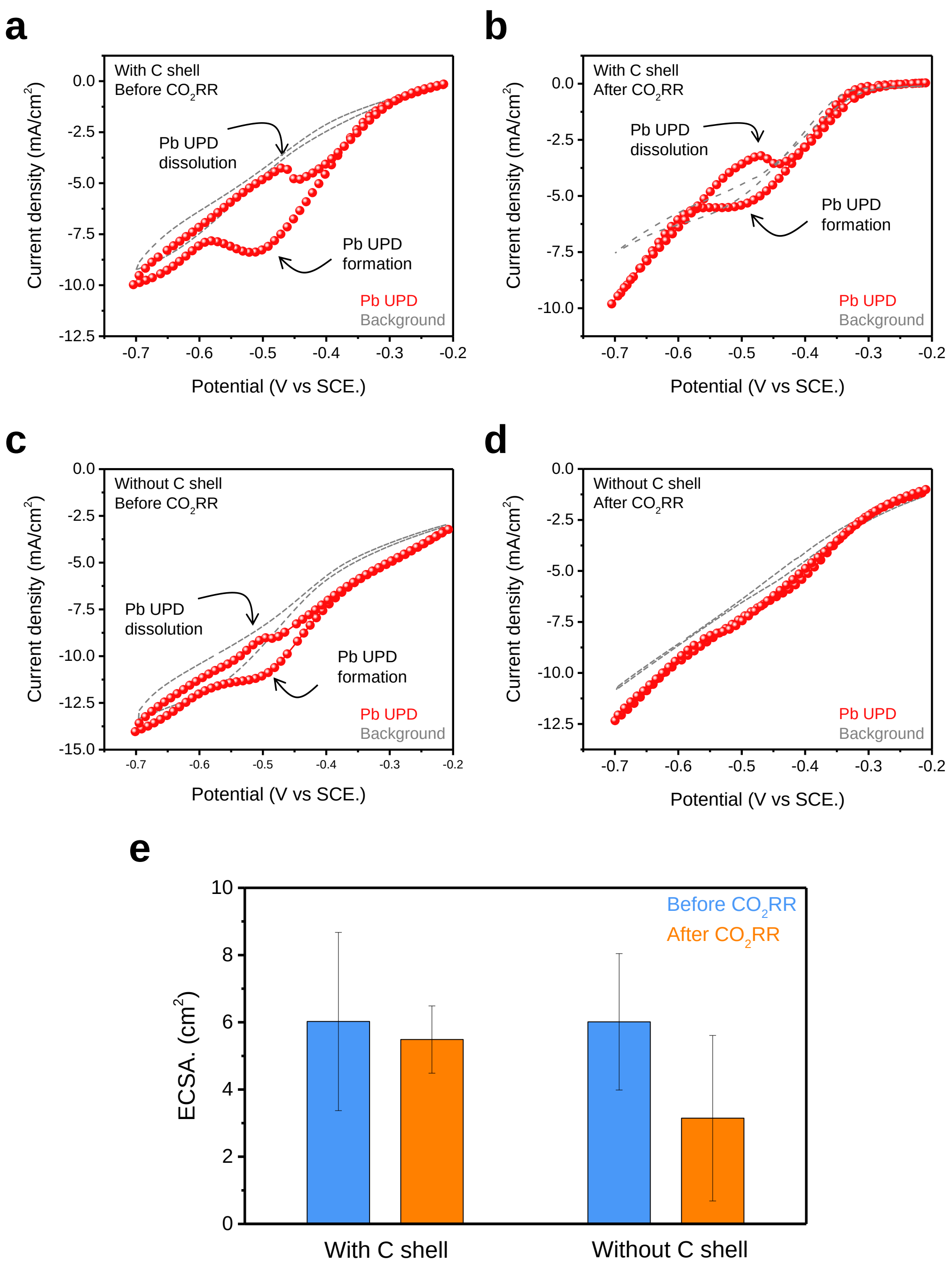

Fig. S9 | Investigation of the C coating effect on the ECSA of Cu nanoparticles during the CO_2RR. a-d, Pb UPD cyclic voltammetry profiles of (**a, b**) quasi-graphitic C shell-coated Cu nanoparticles (with a C shell) and (**c, d**) conventional Cu nanoparticles (without a C shell). The investigations were conducted (**a, c**) before and (**b, d**) after the CO_2RR. Red curves: Pb

UPD formation and dissolution on Cu nanoparticles. Gray curves: background. **e**, ECSA values of Cu nanoparticles calculated from the Pb UPD curves. The Pb adatom coverage of the Cu surface was 310 μC/cm^2 according to previous reports[1]. The error bar indicates the standard deviation of five measurements.

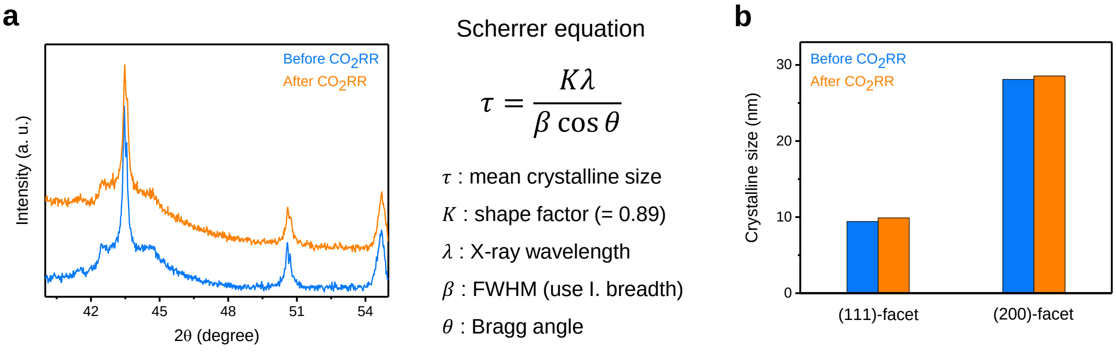

Fig. S10 | XRD spectra and derived crystalline size of carbon-encapsulated Cu nanoparticles before and after CO_2RR. a, XRD spectra for Cu nanoparticles with carbon shell. XRD analysis was conducted before and after CO_2RR. CO_2RR was operated at -2.4 V (vs RHE.) for 1.5 hours in 1M KOH electrolyte. **b**, Comparison of crystalline size calculated from XRD spectra using Scherrer equation. Related equation was described.

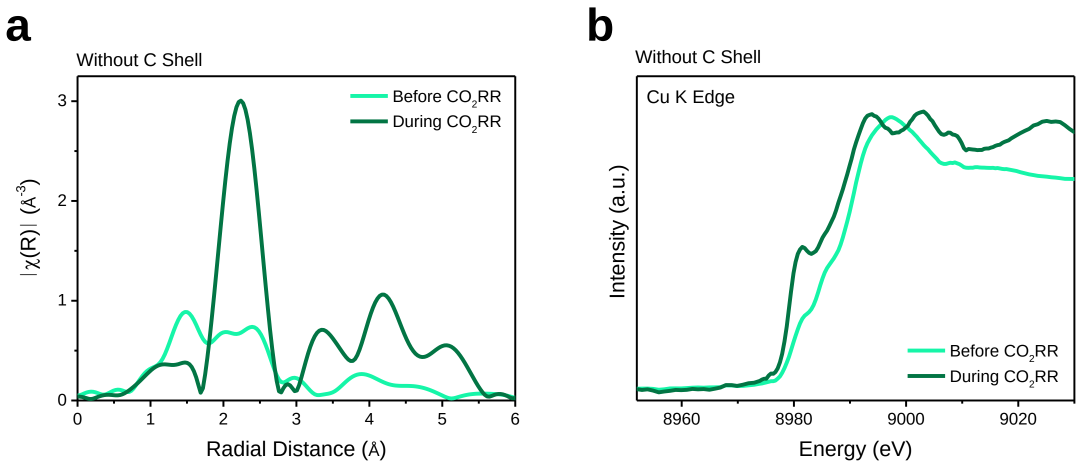

Fig. S11 | *Operando* XAS analysis to investigate the status of conventional Cu nanoparticles during the CO_2RR. a-b, Cu *K*-edge (**a**) EXAFS and (**b**) XANES spectra of conventional Cu nanoparticles (without a C shell) before and during the CO_2RR. CO_2RR was conducted at j = 200 mA/cm^2 in 1 M KOH using the flow cell. The XAS measurement was started 10 min after the initiation of the reaction and was carried out for 35 min.

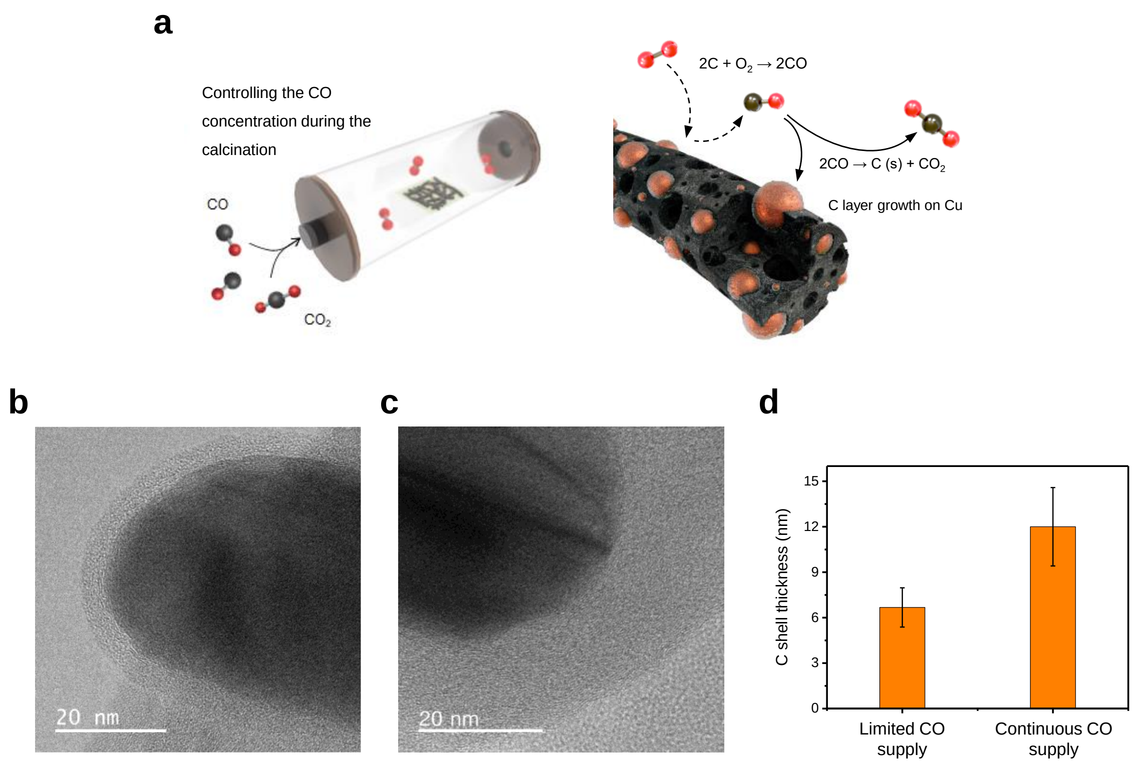

Fig. S12 | Controlling C shell thickness on Cu nanoparticles by modification of the calcination conditions. a, Schematic illustration of the method for controlling C shell thickness. By controlling the concentration of CO during calcination, the thickness of the C shell was successfully controlled. **b-c,** Representative TEM image of the thickness-controlled C shell and inner Cu nanoparticle. The Cu catalysts were coated with a C shell under conditions where (**b**) the CO supply was limited by closing the chamber with regulated pO_2 and (**c**) a CO/CO_2 mixture gas was continuously supplied. **d,** C shell thickness measured from TEM images. It was statistically processed from 5 samples.

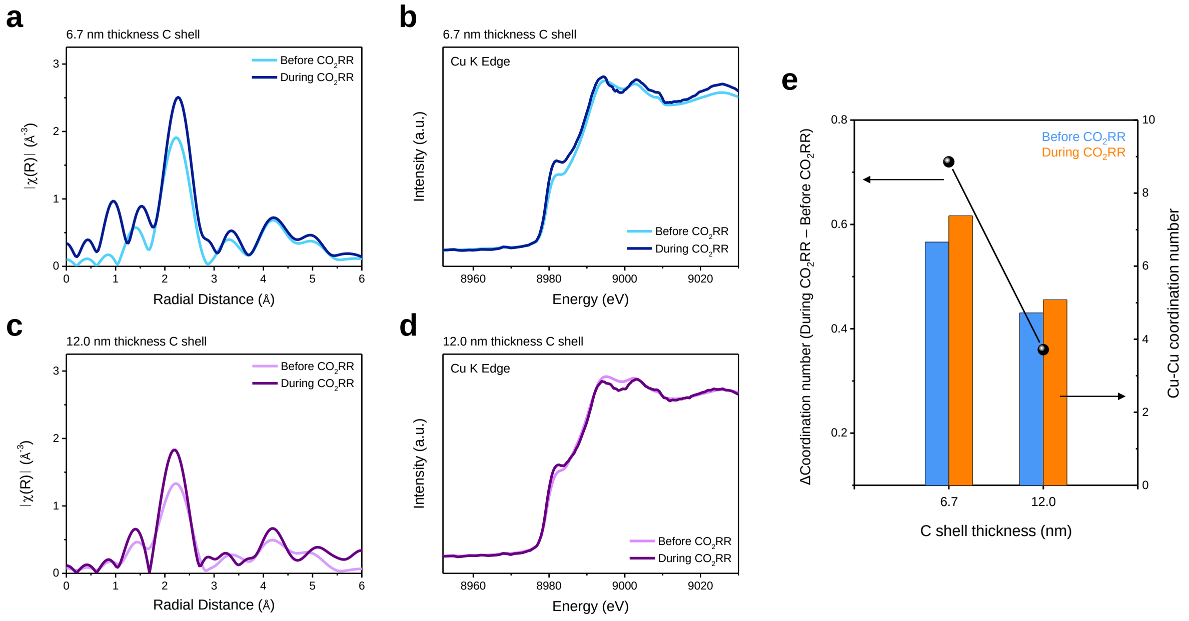

Fig. S13 | Effect of C shell thickness on the ability to suppress surface reconstruction a-d, Cu *K*-edge (**a, c**) EXAFS and (**b, d**) XANES spectra of Cu nanoparticles (**a, b**) with a 6.7 nm thick C shell and (**c, d**) a 12.0 nm thick C shell. Cu states were investigated before and during the CO_2RR. The CO_2RR was conducted at j = 200 mA/cm^2 in a 1 M KOH electrolyte using a flow cell. The XAS measurement was started 10 min after the initiation of the reaction and was carried out for 35 min. **e,** Cu-Cu coordination numbers derived from each EXAFS spectrum. The difference between during and before the CO_2RR was also indicated.

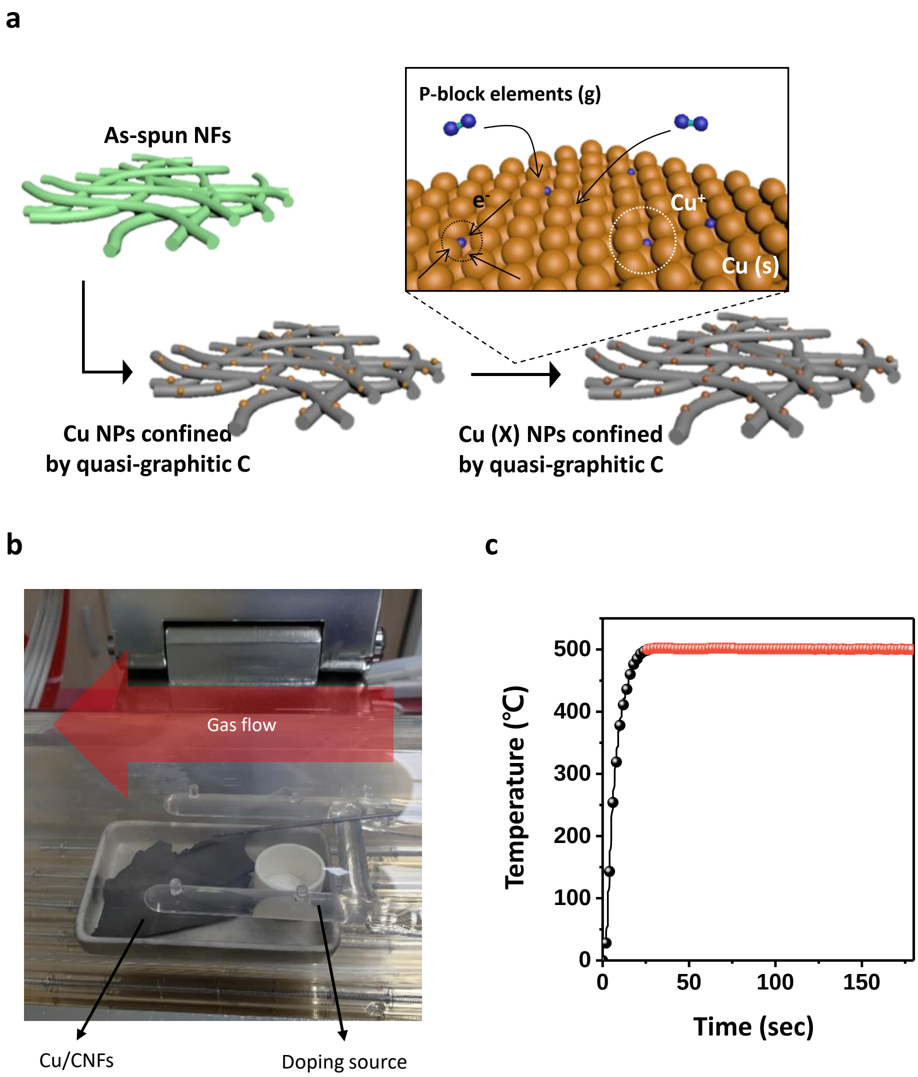

Fig. S14 | Experimental information of p-block element doping process. a, Schematic illustration of the fabrication process. As-spun nanofiber was converted to Cu nanoparticles confined in the C shell through pO_2-controlled calcination, and then the p-block elements were incorporated to modify the surface structure. **b,** image of the equipment for rapid incorporation of p-block elements. **c,** the temperature profile. The reaction was implemented during 180 seconds.

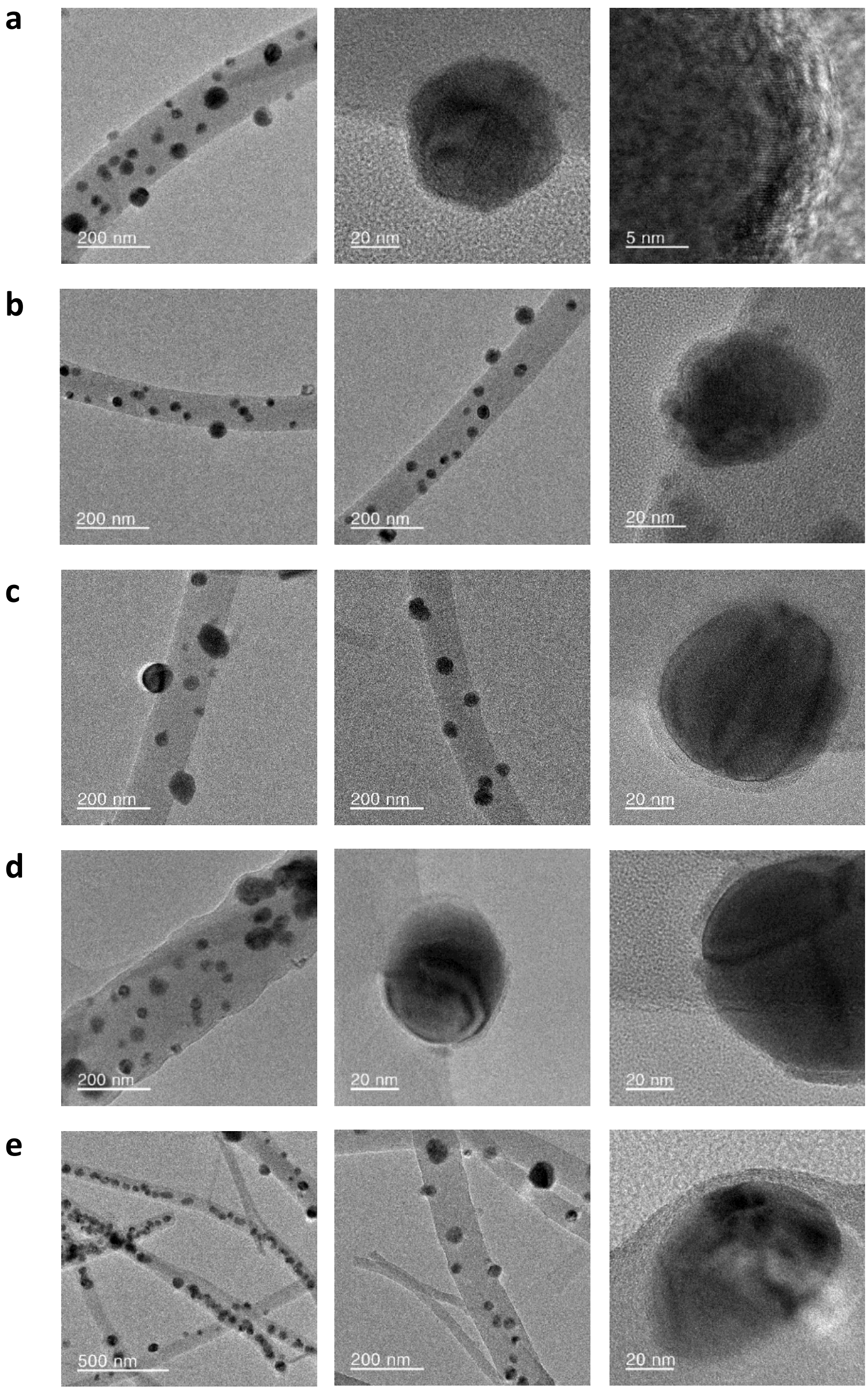

Fig. S15 | Microstructure of p-block elements doped Cu nanoparticles. HR-TEM image of Cu nanoparticles after doping each p-block elements. (**a**) Cu (P), (**b**) Cu (F), (**c**) Cu (B), (**d**) Cu (Cl), and (**e**) Cu (N). It was confirmed that the morphology was maintained by the Cu confinement effect.

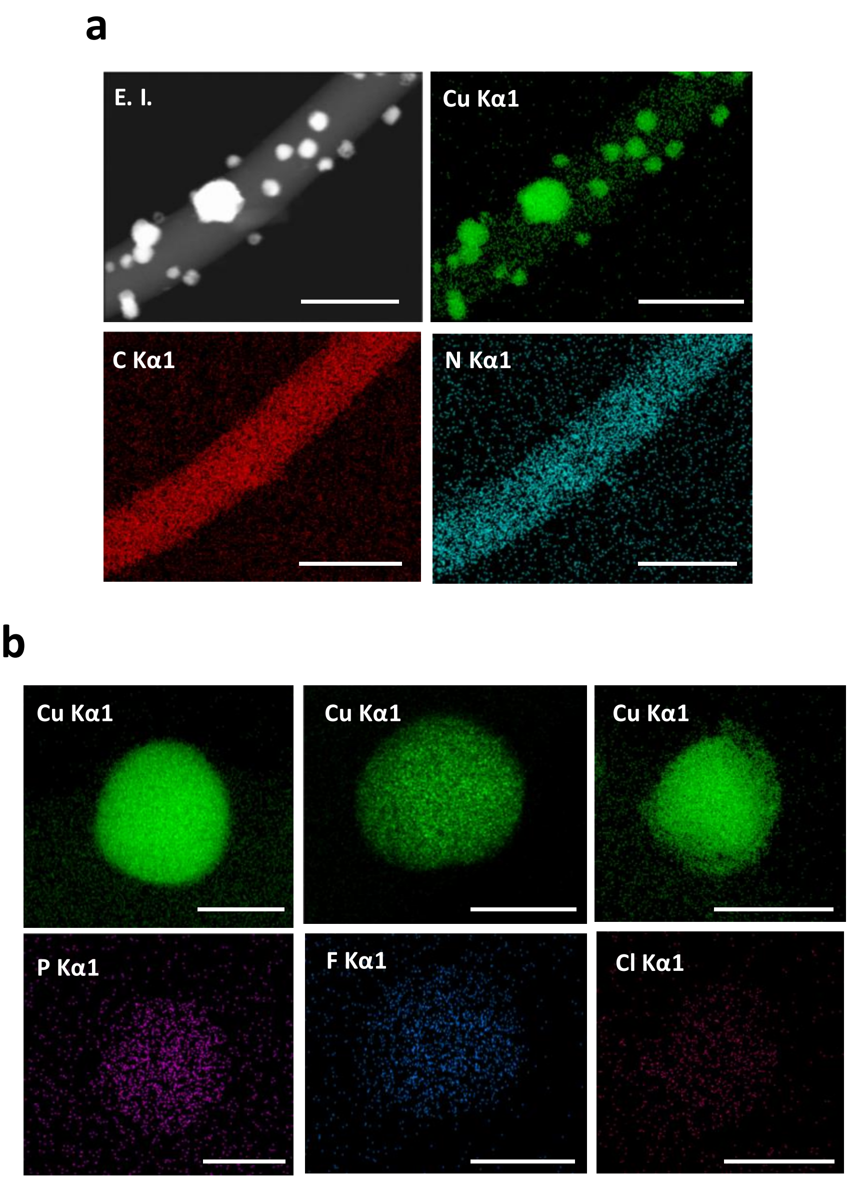

Fig. S16 | TEM EDS mapping image of p-block elements doped Cu nanoparticles. a, TEM EDS elemental mapping of Cu nanoparticles confined by C support with N defect. **b,** EDS mapping images showed elemental doping on Cu nanoparticles.

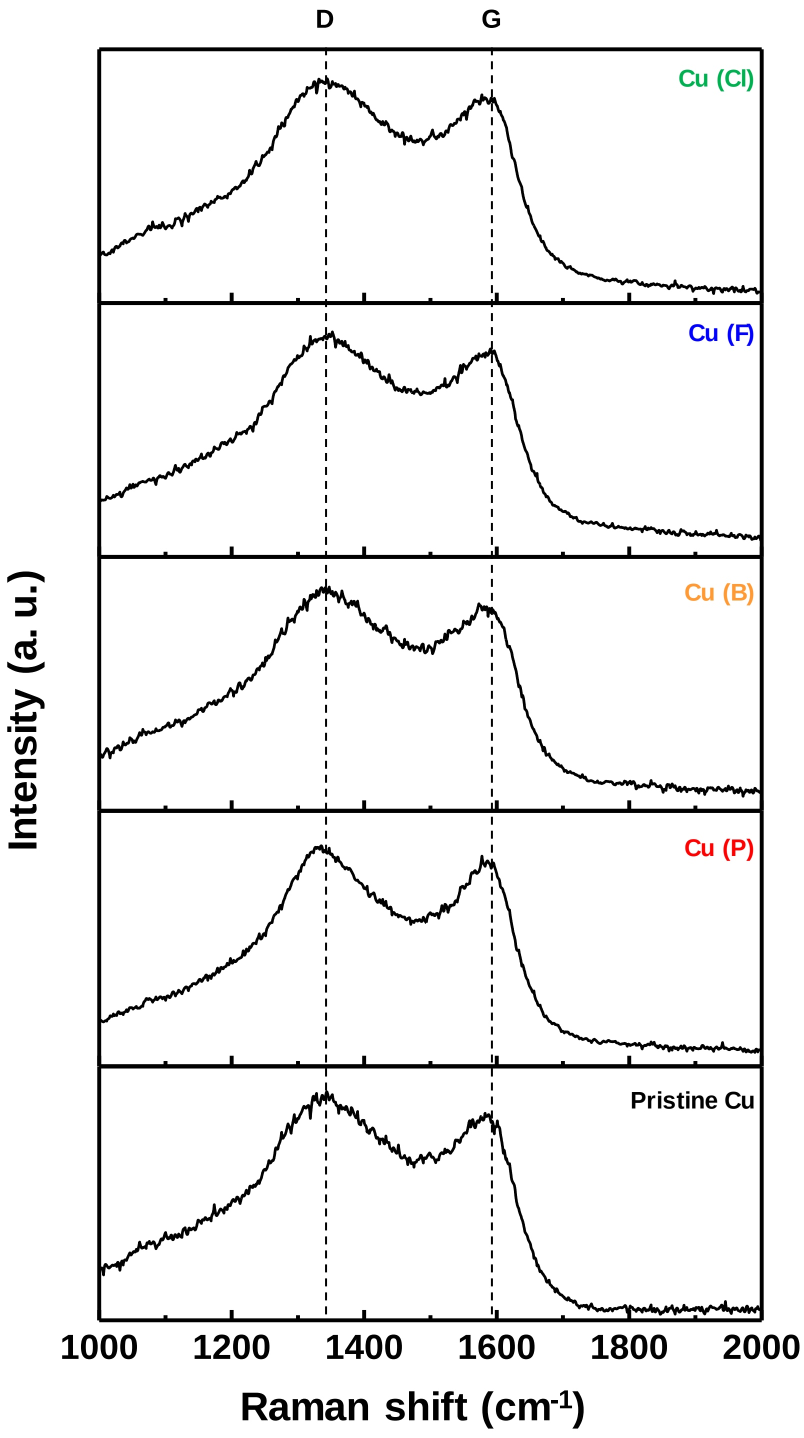

Fig. S17 | Bonding characteristics of carbon support. Bonding nature of each carbon support was investigated by Raman spectroscopy. Support C framework was amorphous and did not altered after the doping reaction.

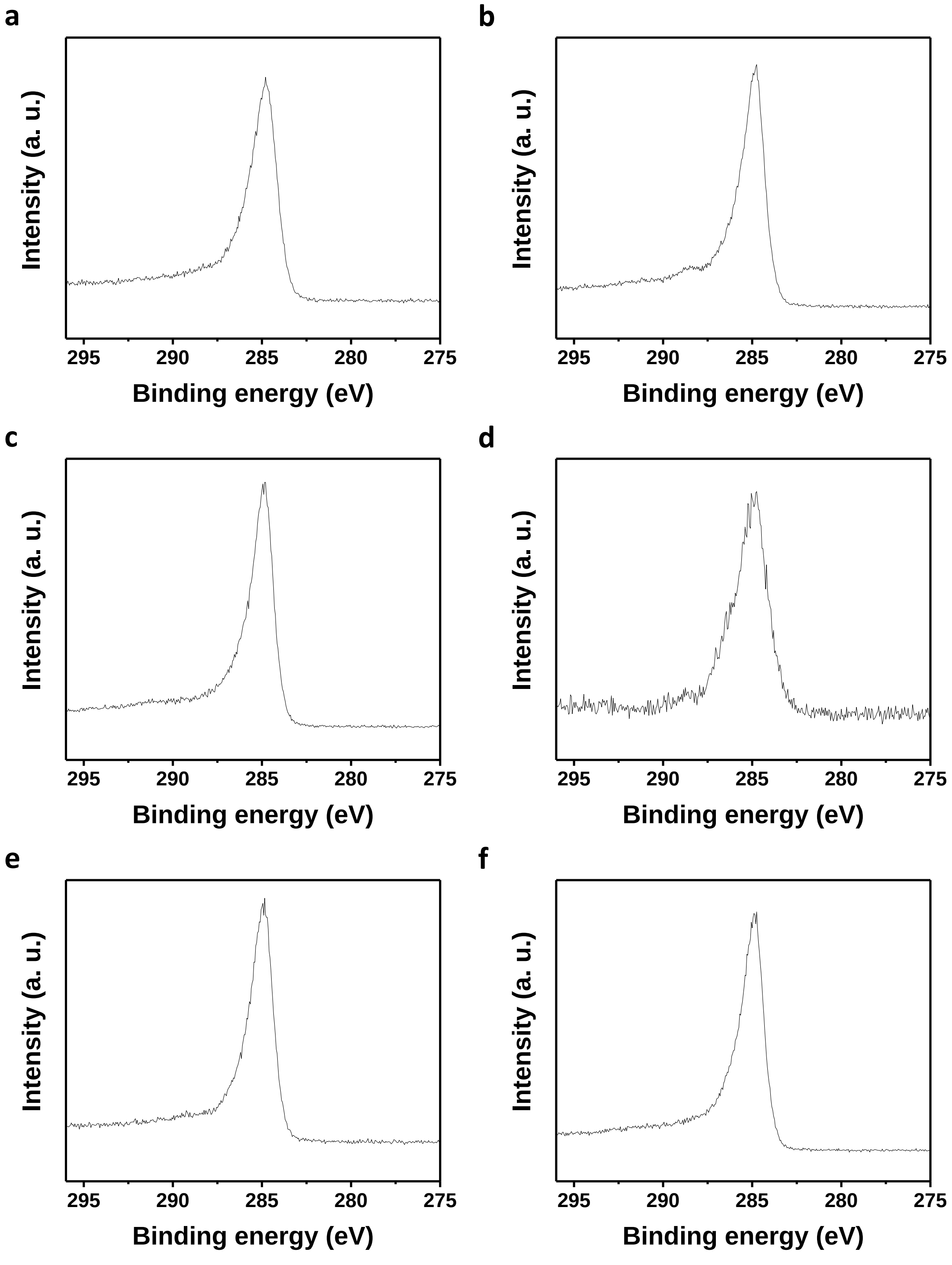

Fig. S18 | C 1s XPS spectra of confined Cu nanoparticles. a, Pristine Cu and *p*-block elements doped Cu nanoparticles, (**b**) Cu (P), (**c**) Cu (F), (**d**) Cu (B), (**e**) Cu (Cl), and (**f**) Cu (N).

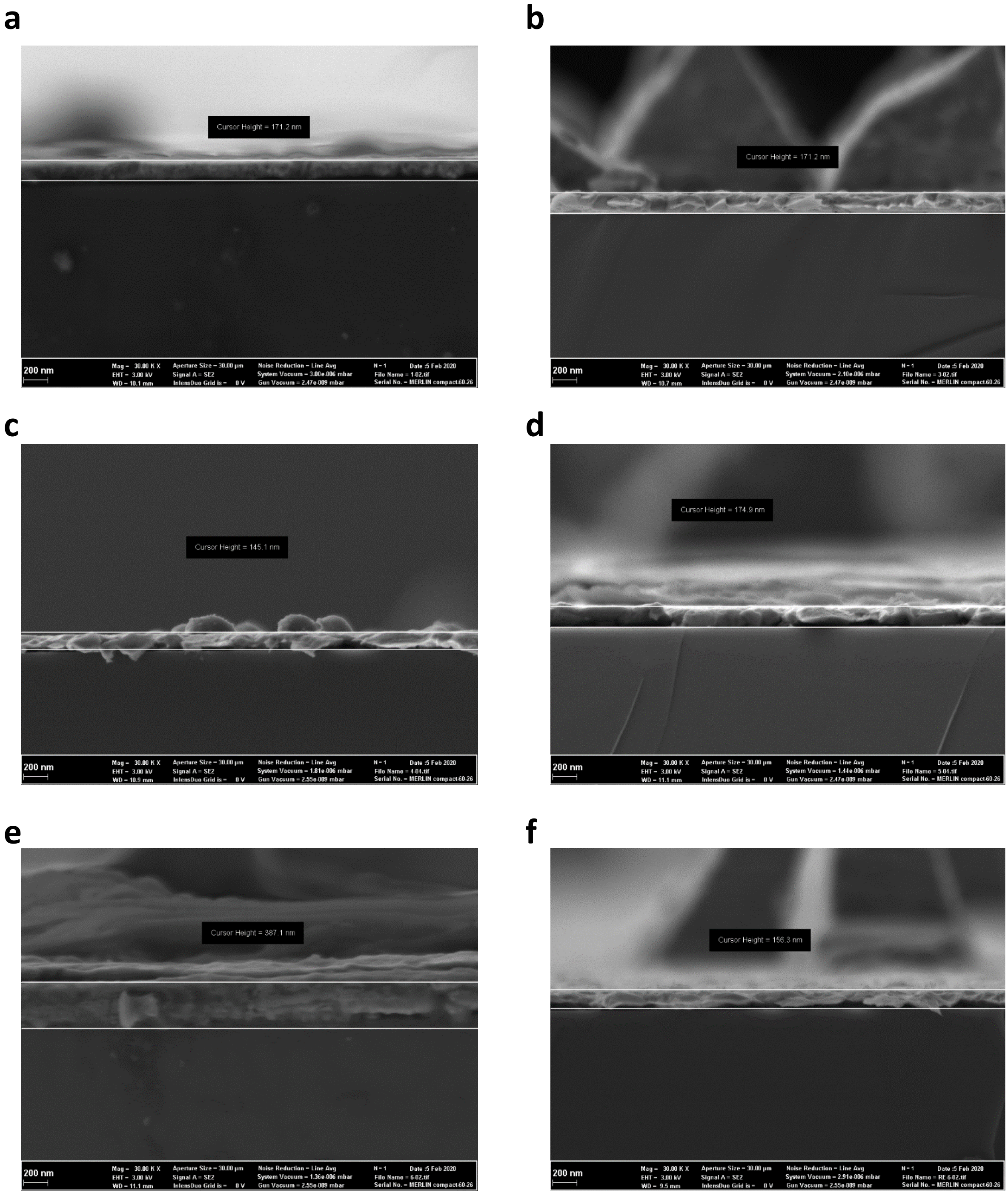

Fig. S19 | Incorporation of p-block element into Cu thin film. Vertical SEM image of (**a**) pristine Cu film, (**b**) Cu (P) film, (**c**) Cu (F) film, (**d**) Cu (B) film, (**e**) Cu (Cl) film, and (**f**) Cu (N) film. The Cu film was evaporated on SiO_2 wafer with 8 Å/s of deposition rate. The doping reaction likewise was conducted at 500°C for 180 seconds.

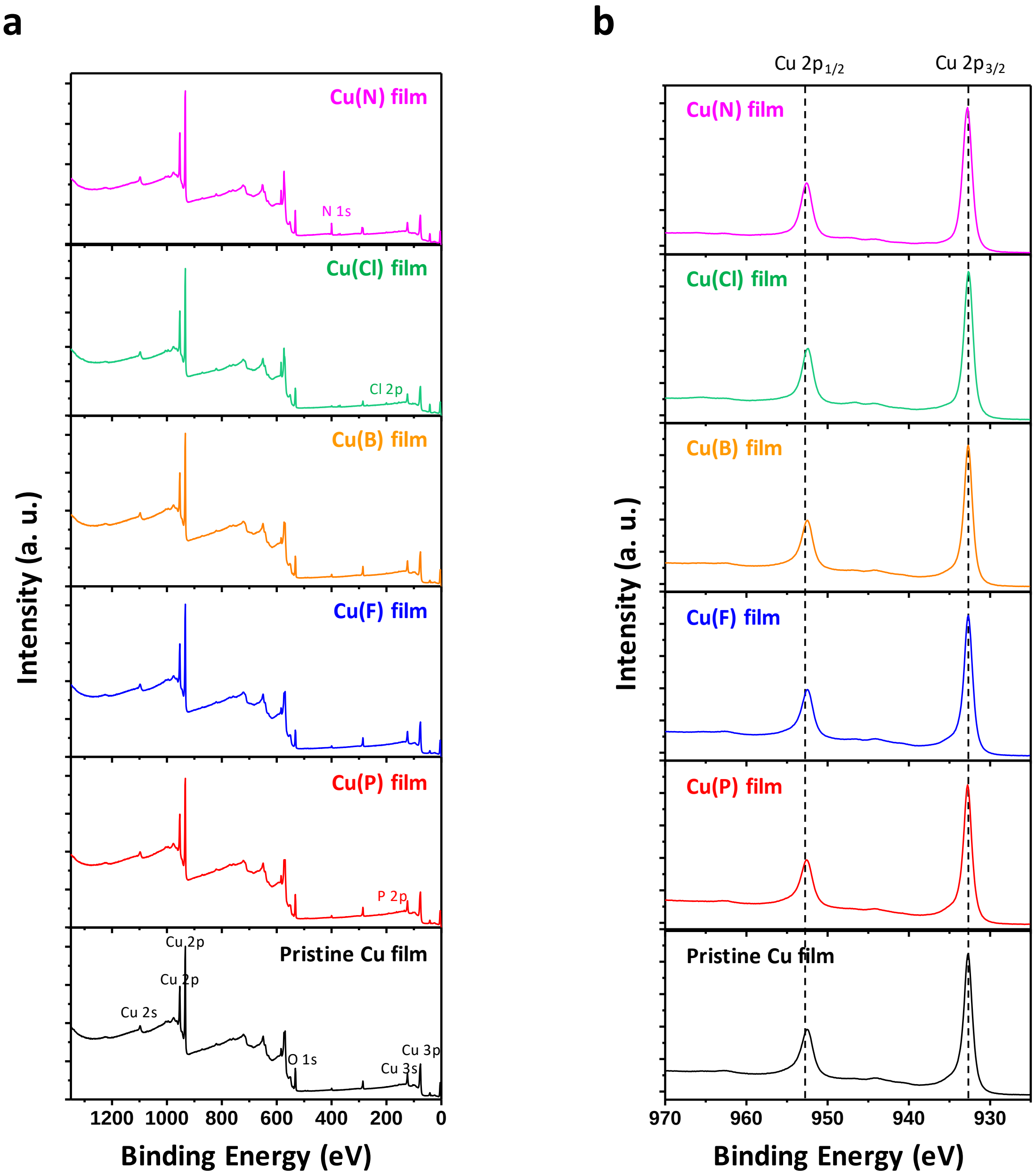

Fig. S20 | Chemical state investigation on Cu (X) film. a, Overall XPS spectra and (**b**) Cu *2p* peak of pristine Cu and Cu (X) film.

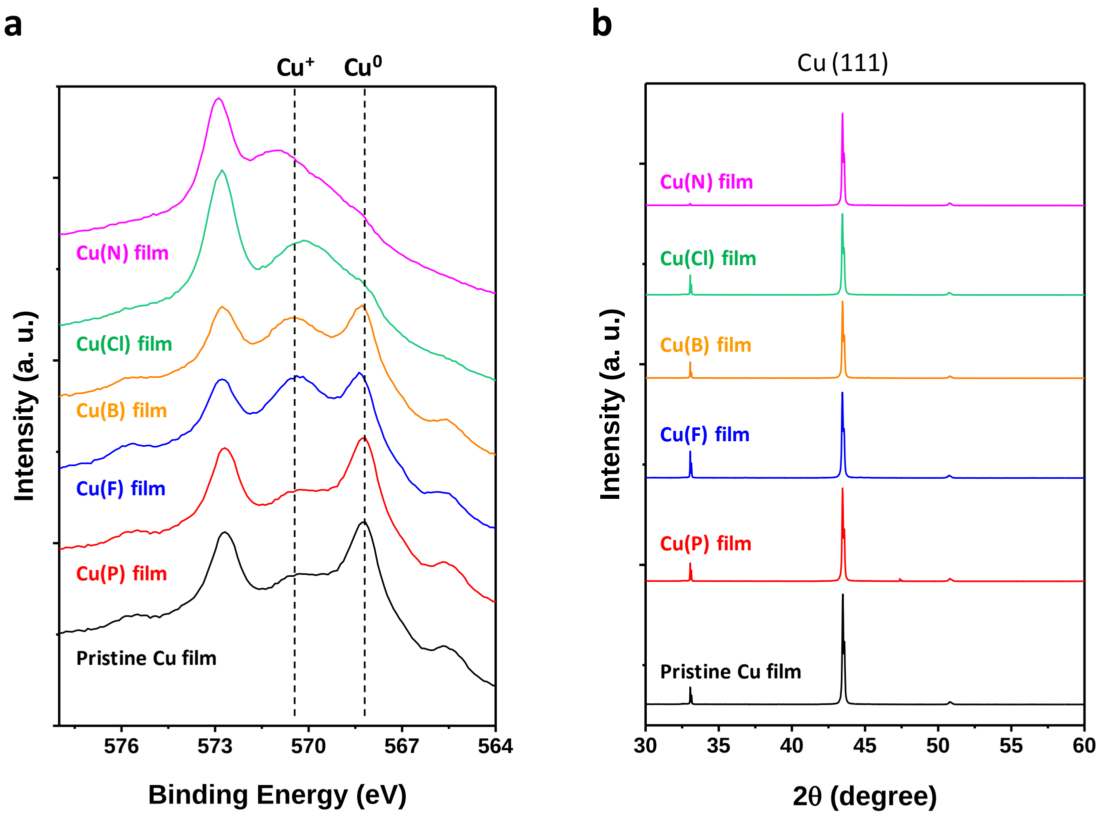

Fig. S21 | Chemical state and Phase investigation on Cu (X) film. a, XPS Cu *LMM* and (**b**) XRD spectra of pristine and Cu (X) film.

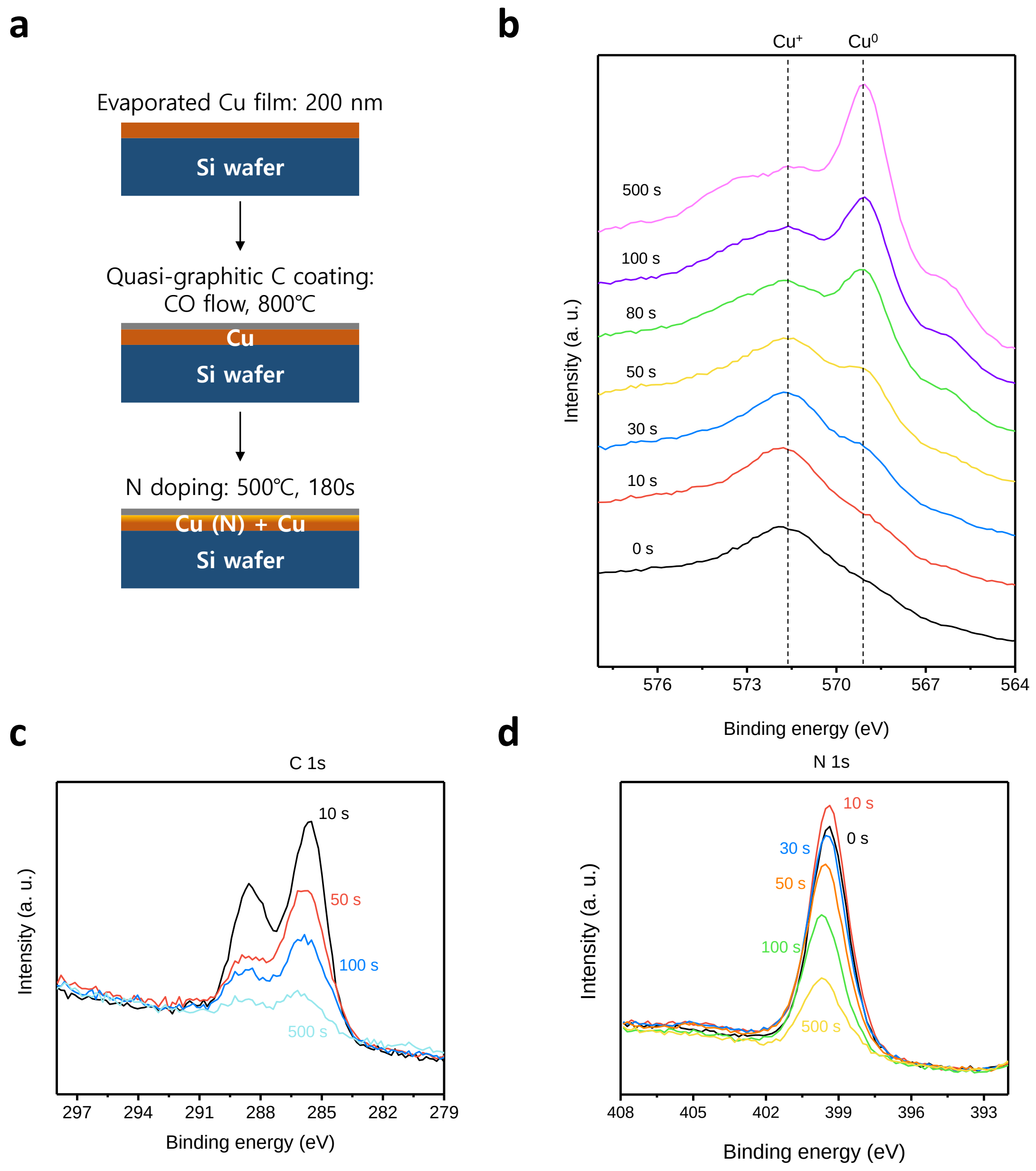

Fig. S22 | XPS depth profile on Cu (N) film. a, Fabrication schematic of confined Cu (N) film. To develop C shell on Cu film, annealing under CO flow was conducted at 800℃. (**b**) Cu *LMM*, (**c**) C *1s*, and (**d**) N *1s* spectra as etching the film.

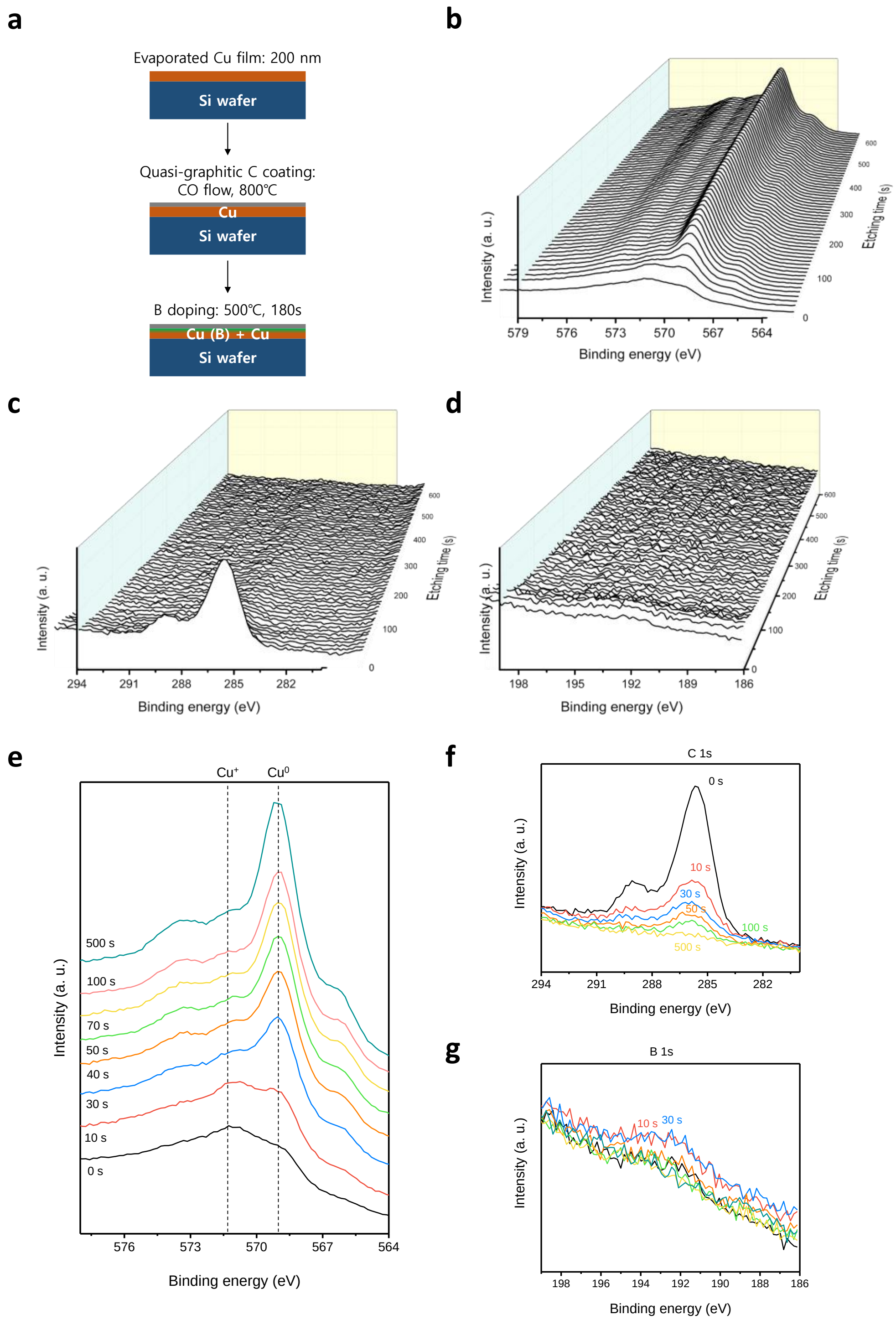

Fig. S23 | XPS depth profile on Cu (B) film. a, Fabrication schematic of confined Cu (B) film. To develop C shell on Cu film, annealing under CO flow was conducted. (**b, e**) Cu *LMM*, (**c, f**) C *1s*, and (**d, g**) B *1s* spectra as etching the film.

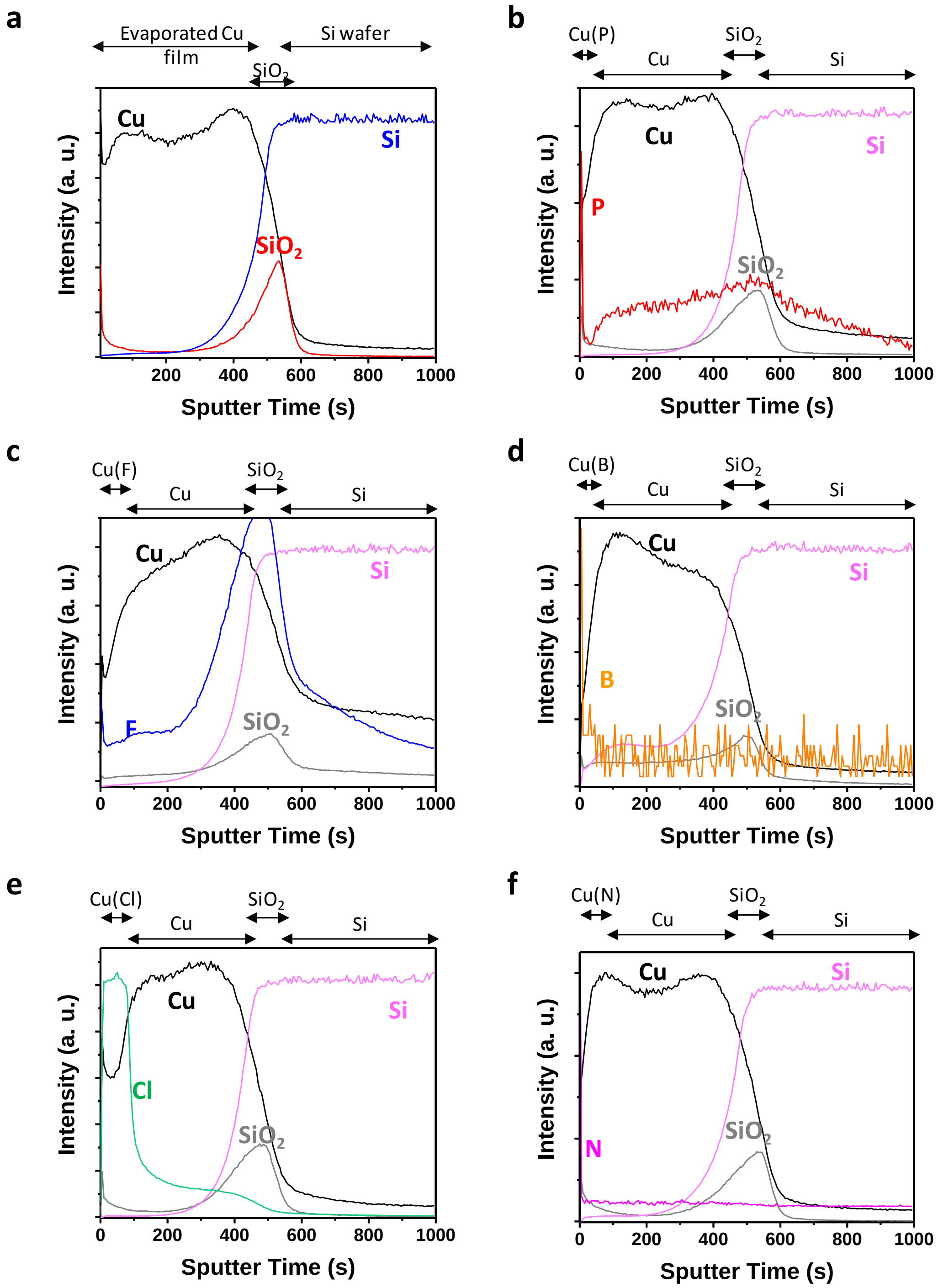

Fig. S24 | Depth profile of Cu (X) film. Depth profile was examined through time-of-flight secondary ion mass spectroscopy (TOF-SIMS). The etching source and area was Cs^+ ion and 100×100 μm^2. Depth profile of (**a**) pristine Cu film, (**b**) Cu (P) film, (**c**) Cu (F) film, (**d**) Cu (B) film, (**e**) Cu (Cl) film, and (**f**) Cu (N) film.

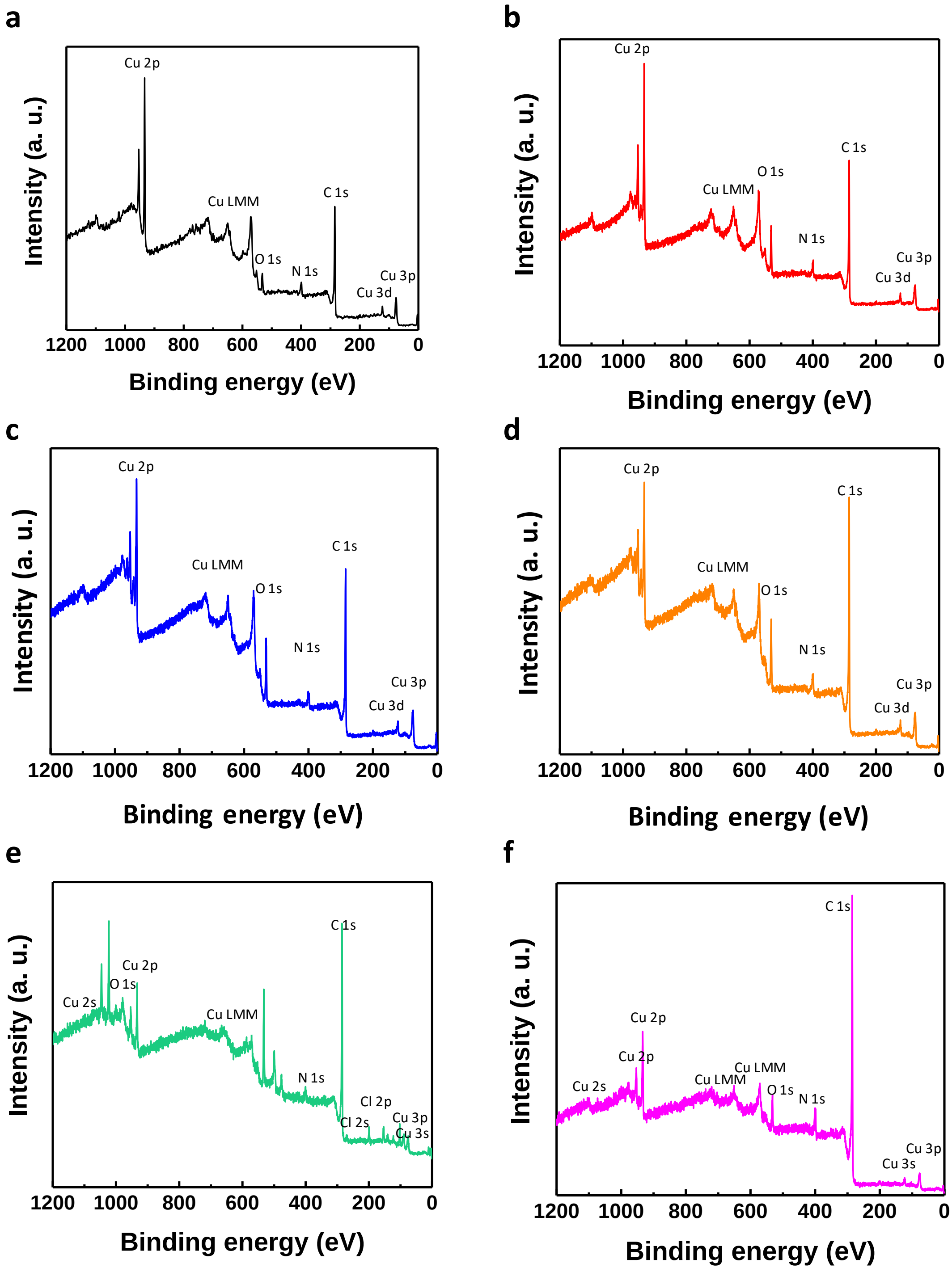

Fig. S25 | Chemical state investigation of quasi-C shell coated Cu nanoparticles after doping. XPS spectra of pristine Cu and Cu (X). (**a**) pristine Cu, (**b**) Cu (P), (**c**) Cu (F), (**d**) Cu (B), (**e**) Cu (Cl), and (**f**) Cu (N), respectively.

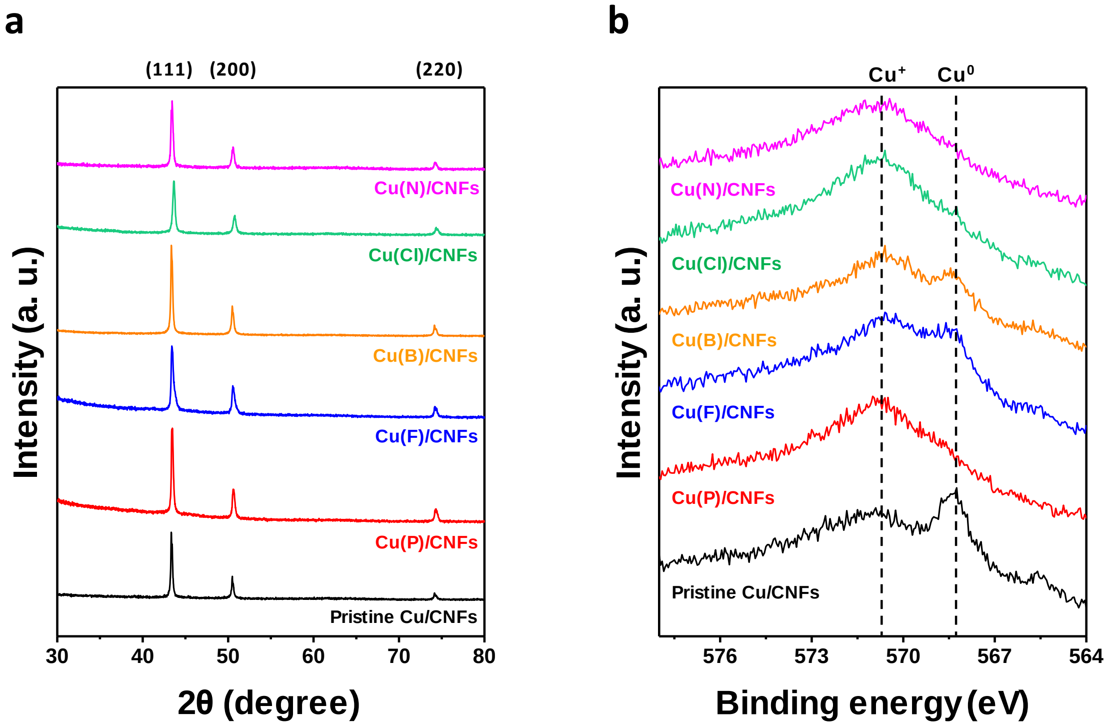

Fig. S26 | Phase and oxidation states analysis with XPS Cu *LMM* spectra in p-block elements doped Cu nanoparticles. (**a**) XRD and (**b**) XPS Cu *LMM* spectra of confined Cu nanoparticles doped with each p-block element.

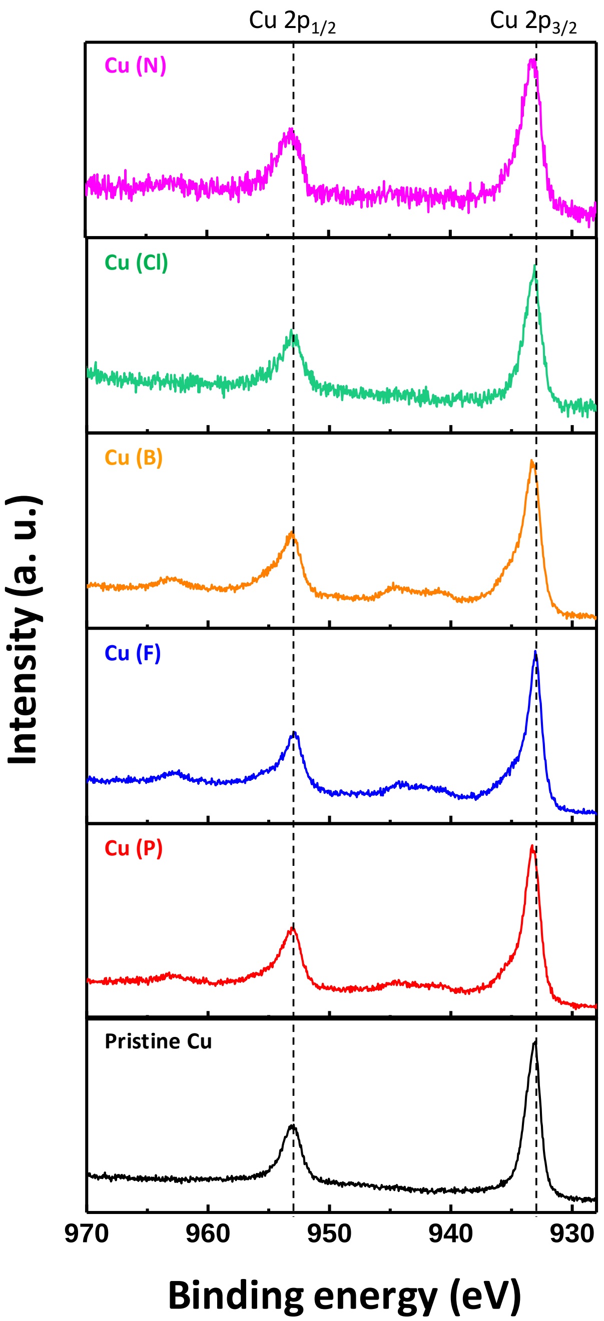

Fig. S27 | Oxidation states analysis with XPS Cu *2p* spectra in *p*-block elements-doped Cu nanoparticles. Comparison of XPS Cu *2p* spectra according to the condition of doping elements.

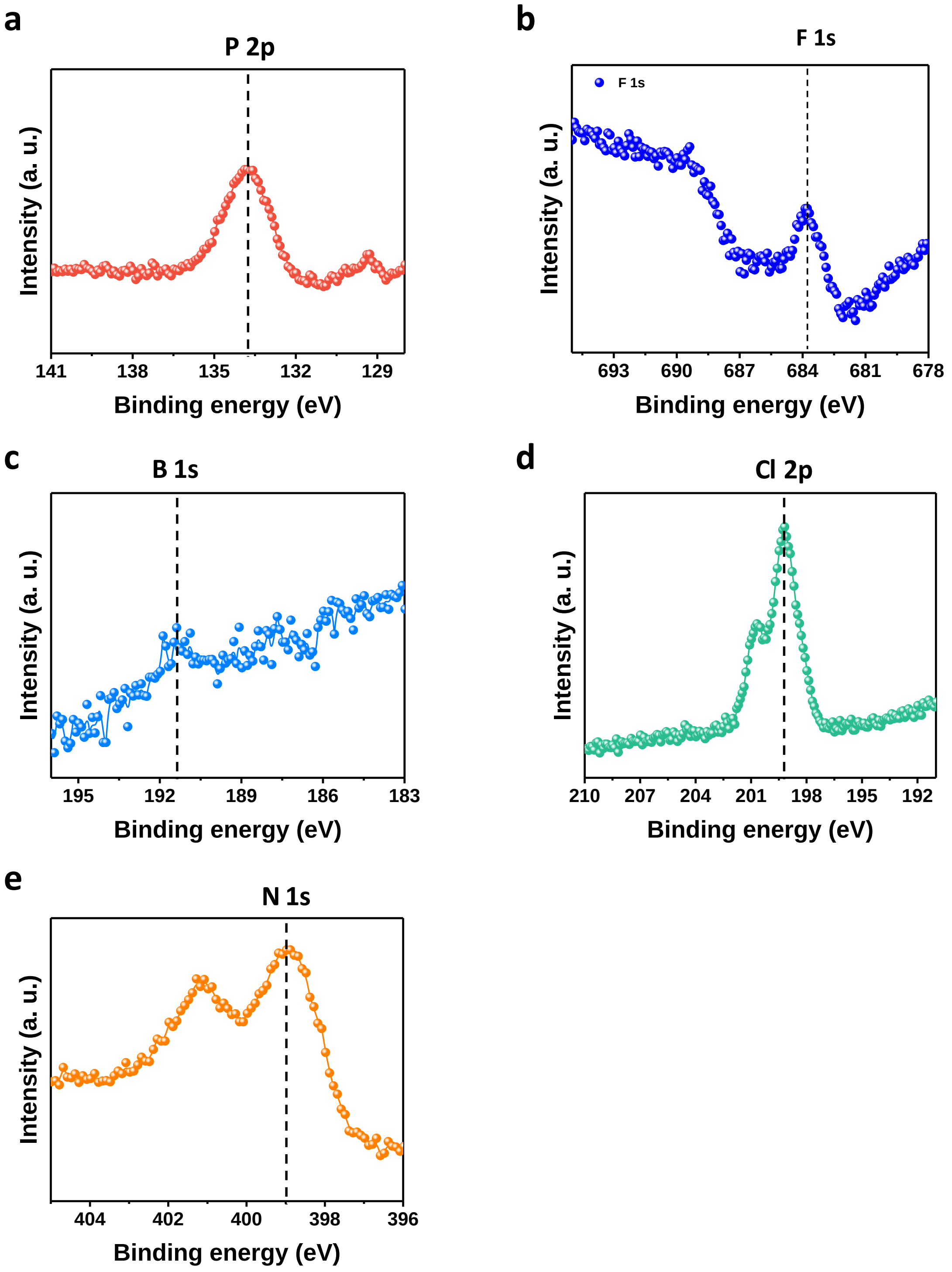

Fig. S28 | Verification of dopant in Cu (X). XPS spectra of each dopants. (**a**) P *2p*, (**b**) F *1s*, (**c**) B *1s*, (**d**) Cl *2p*, and (**e**) N *1s*.

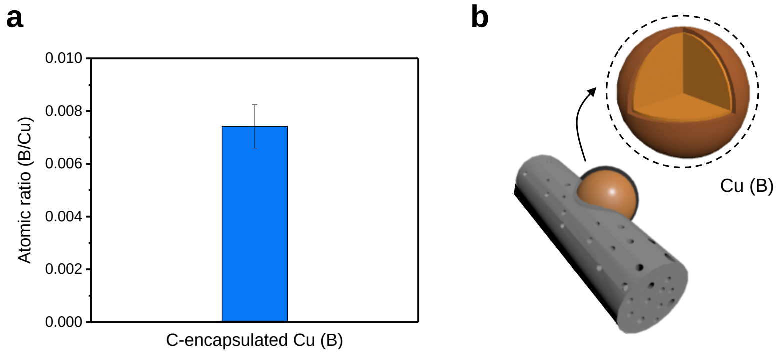

Fig. S29 | Doping concentration and schematic structure of Cu (B). a, Relative atomic ratio of boron to Cu measured by inductively coupled plasma atomic emission spectroscopy (ICP-AES). Error bar indicates the standard deviation of three independent measurements. **b,** Schematic structure of Cu (B) nanoparticles with a quasi-graphitic C shell.

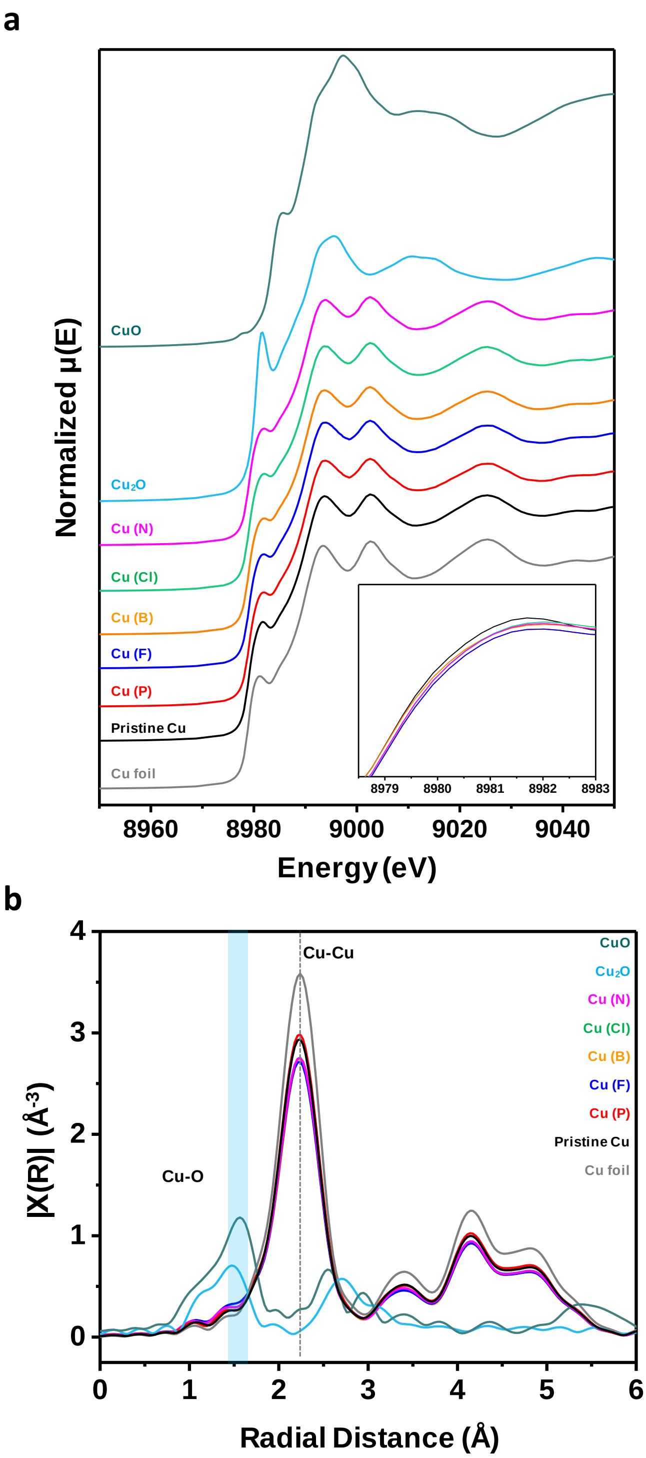

Fig. S30 | Chemical state investigation on Cu (X). (**a**) XANES and (**b**) EXAFS spectra of *p*-block elements doped Cu nanoparticles with C shell and references.

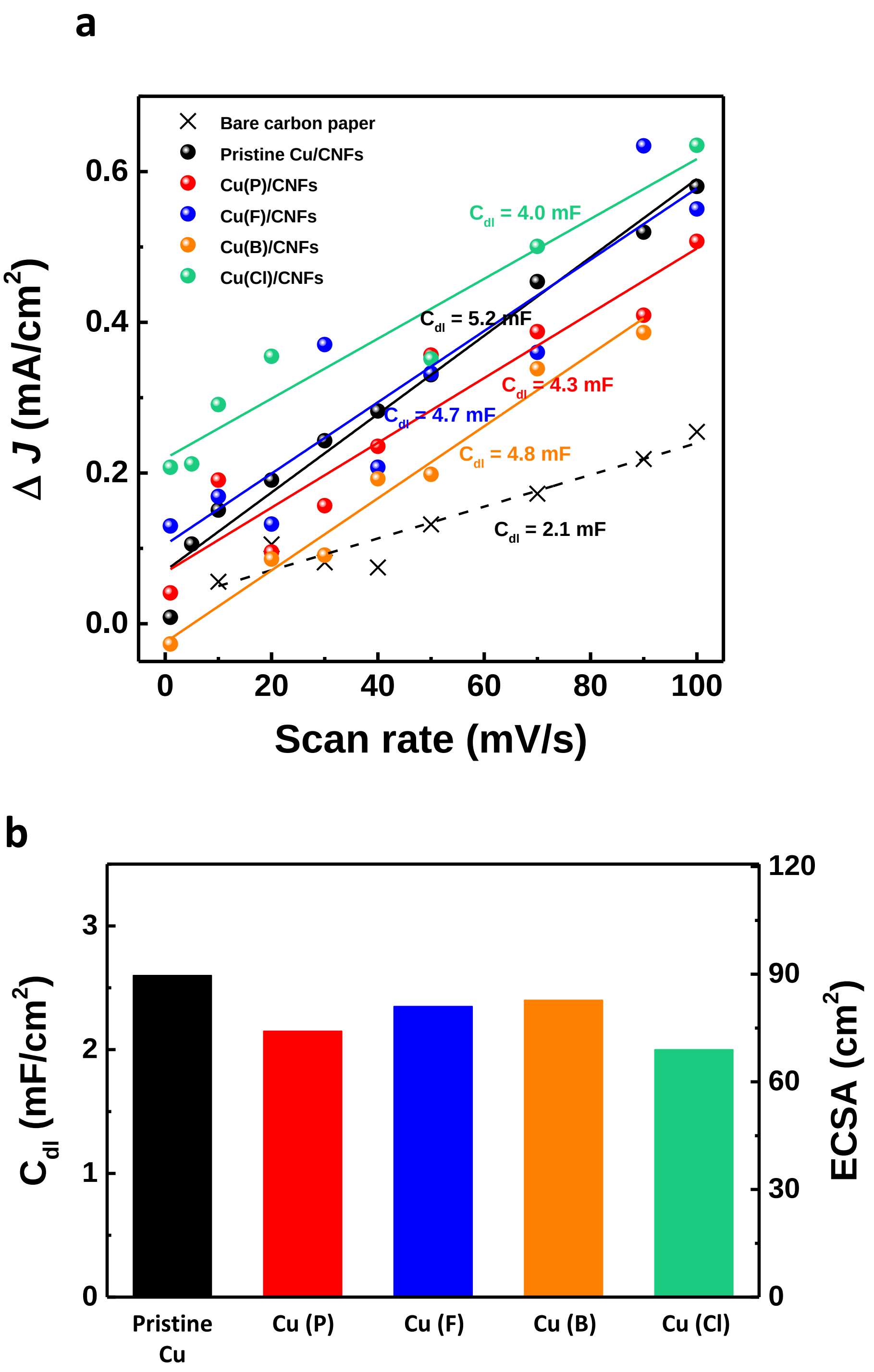

Fig. S31 | Electrochemically active surface area of Cu nanoparticles. (**a**) determination of ECSA by measuring double layer capacitance. (**b**) measured ECSA value of pristine and *p*-block elements doped Cu nanoparticles.

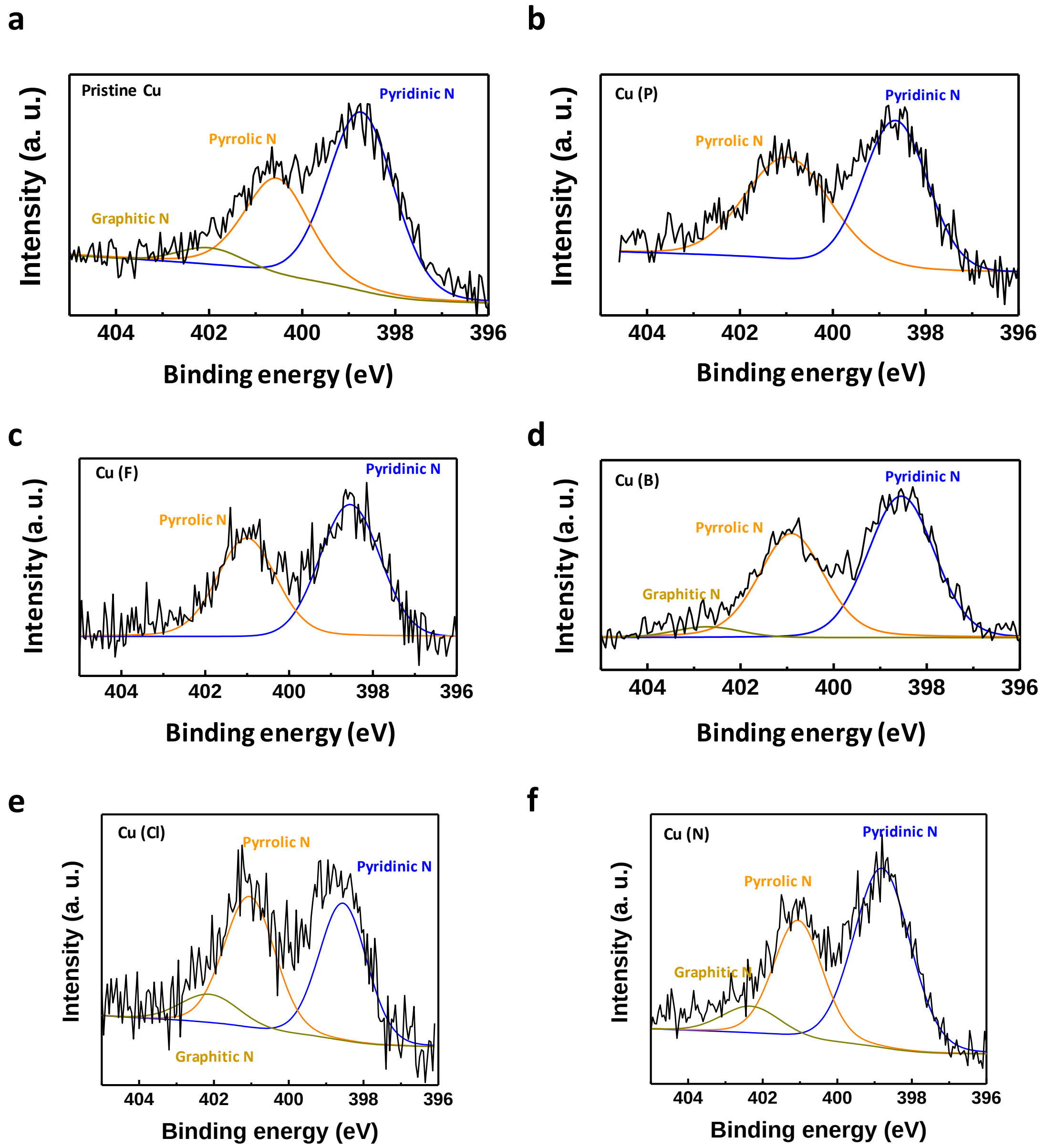

Fig. S32 | N defect configuration in C support framework. N *1s* XPS spectra of confined Cu nanoparticles doped with p-block element. (**a**) pristine Cu nanoparticles, (**b**) Cu (P), (**c**) Cu (F), (**d**) Cu (B), (**e**) Cu (Cl), and (**f**) Cu (N). Deconvolution of N *1s* spectra revealed the configuration of N defect. Pyridinic N site was dominant.

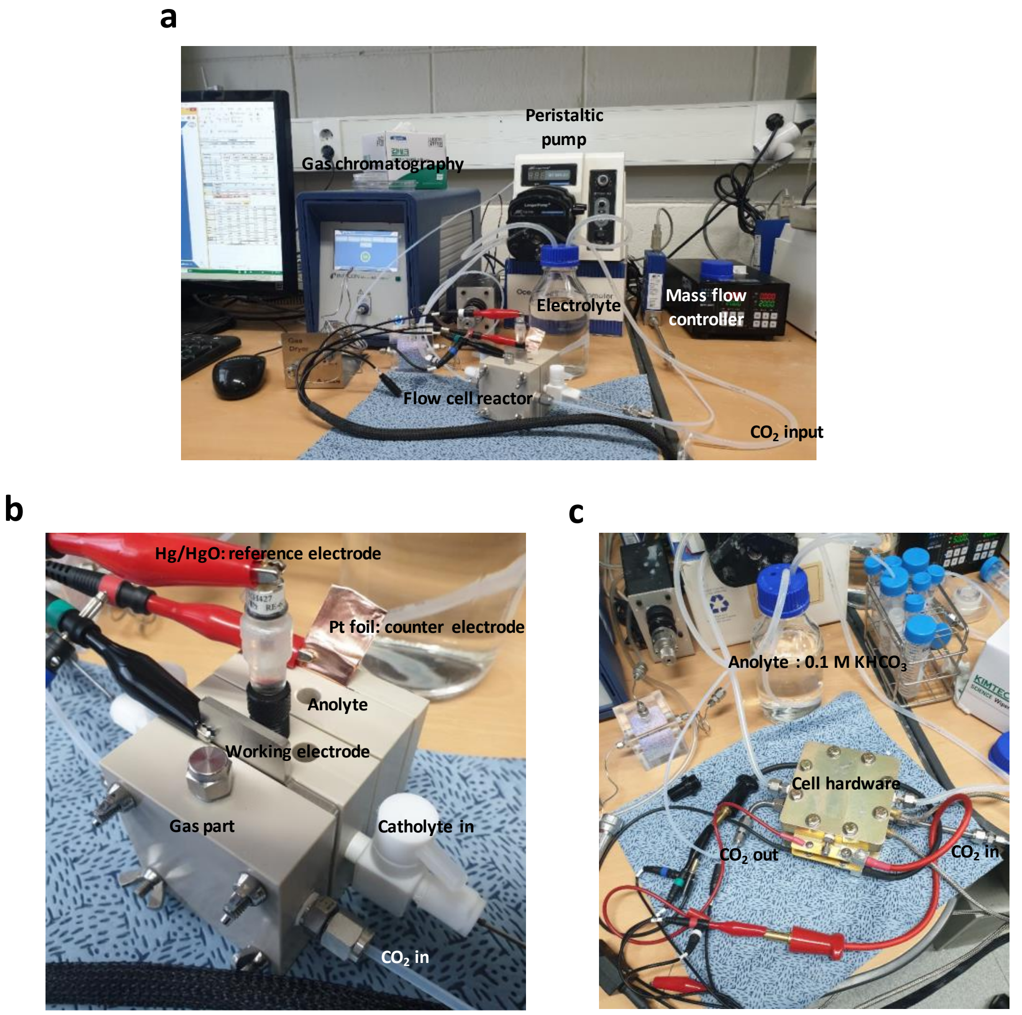

Fig. S33 | Image of CO_2RR system. (**a**) image of CO_2RR system, (**b**) flow cell reactor, and (**c**) membrane electrode assembly (MEA).

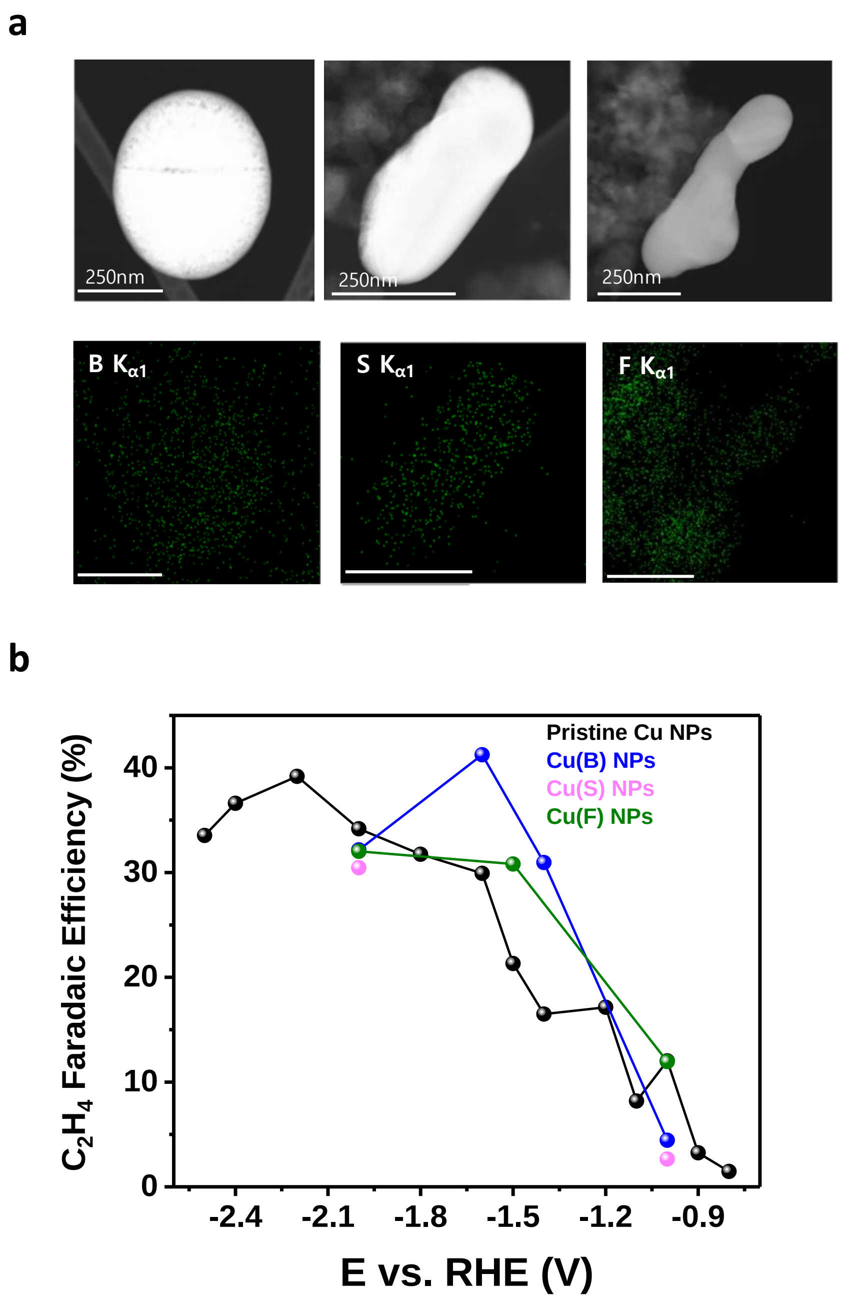

Fig. S34 | Microstructure and CO2RR performance of unconfined Cu nanoparticles doped with *p*-block element. a, EDS mapping image of B, S, and F doped Cu nanoparticles, respectively. **b,** their electrocatalytic CO_2-to-C_2H_4 performance . Agglomeration was occurred and, Cu nanoparticles that were not confined had little doping effect.

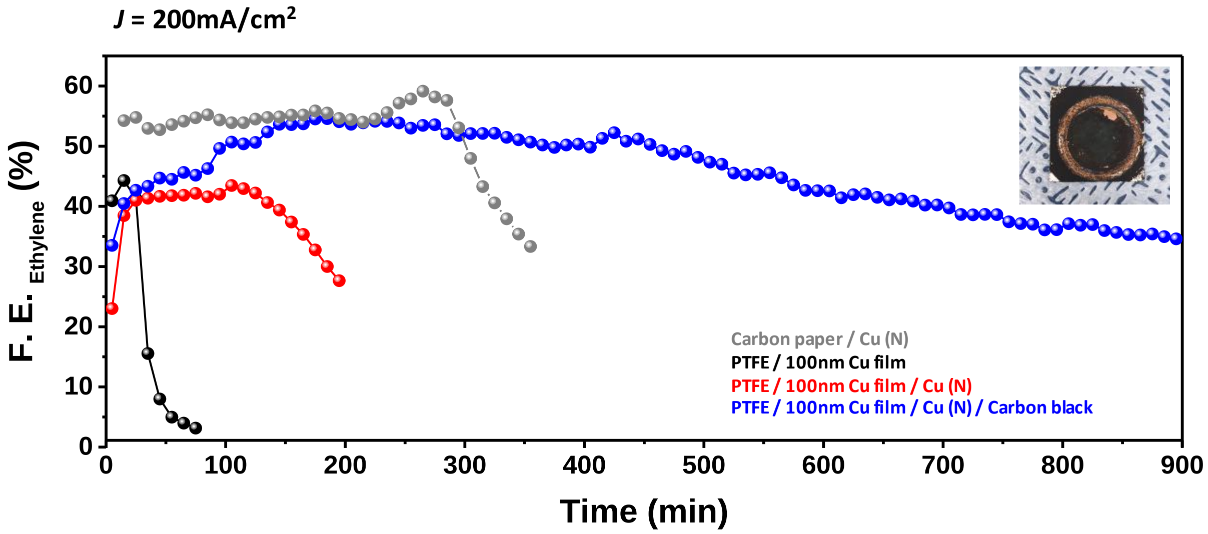

Fig. S35 | CO_2RR stability test to improve stability in flow cell. GDE was fabricated on PTFE fiber. Test was conducted under constant $j = 200$ mA/cm^2 using alkaline flow cell reactor. The electrolyte was 1 M KOH. Inset showed electrode image after the reaction. Poor adhesion caused delamination of catalysts.

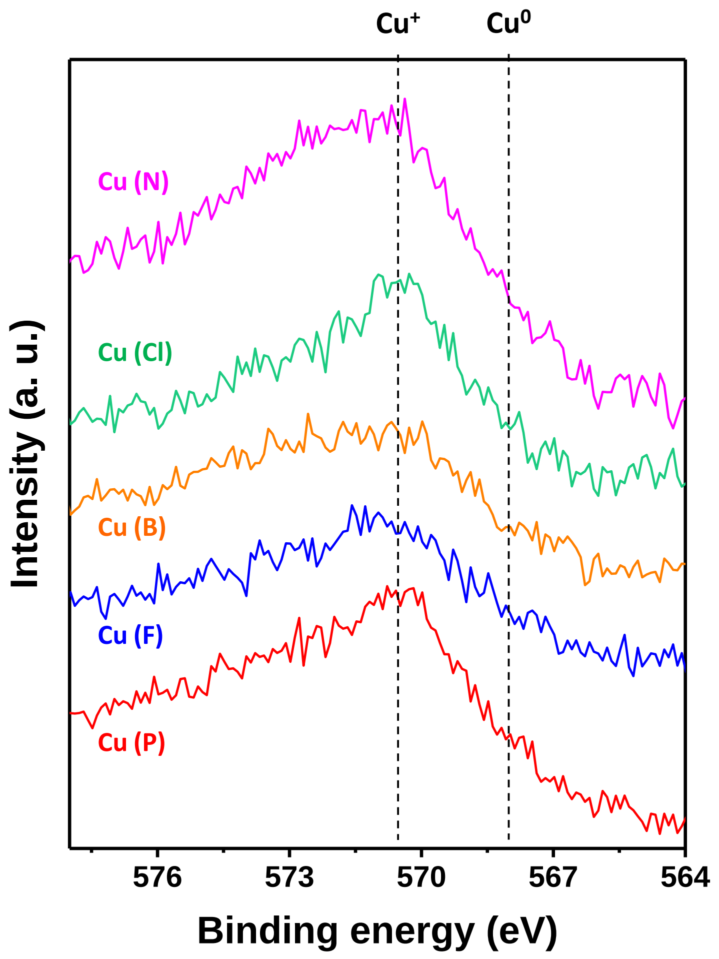

Fig. S36 | Investigation on chemical state transition of Cu (X) after CO_2RR. XPS Cu *LMM* spectra of confined Cu nanoparticles doped with p-block element after CO_2RR. The CO_2RR was conducted under constant j = 200 mA/cm^2 for 5 hours.

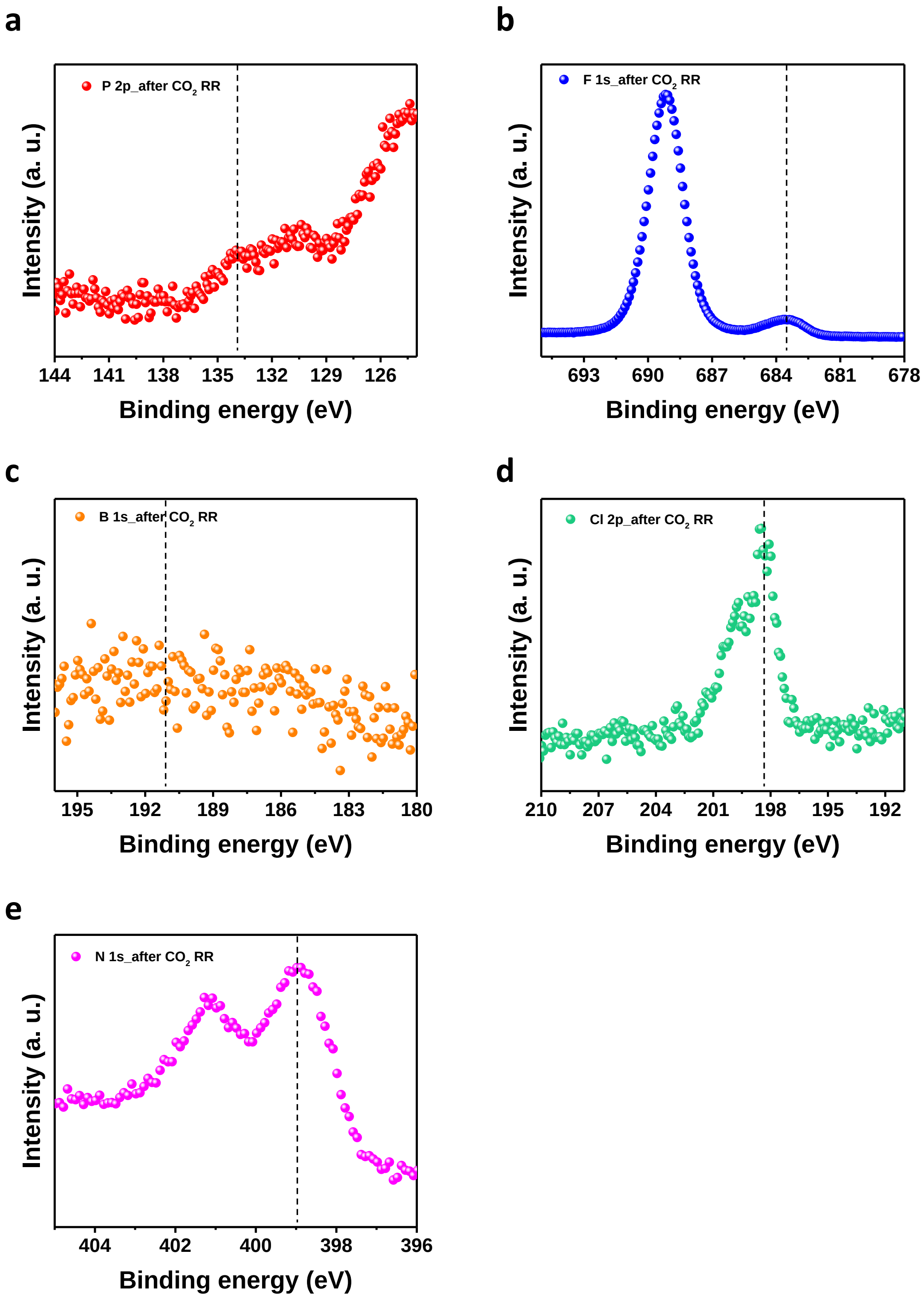

Fig. S37 | Verification of dopant in Cu (X) after CO_2RR. XPS spectra of each dopants. (**a**) P *2p*, (**b**) F *1s*, (**c**) B *1s*, (**d**) Cl *2p*, and (**e**) N *1s*. The CO_2RR was conducted under constant j = 200 mA/cm^2 for 5 hours.

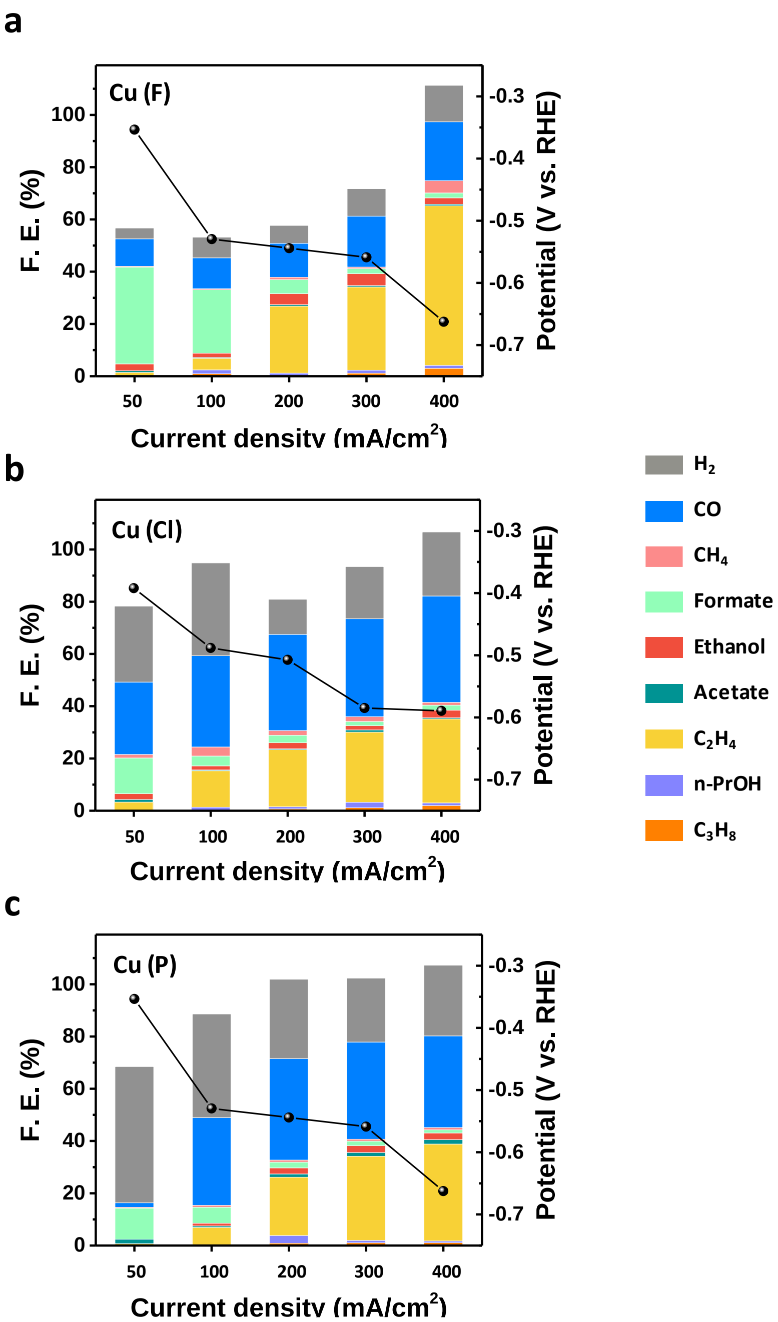

Fig. S38 | CO_2RR performance of p-block elements doped Cu. CO_2RR activity of **(a)** Cu (F), (**b**) Cu (Cl), and (**c**) Cu (P) was investigated through Galvanostatic method under *j* from 50 to 400 mA/cm^2.

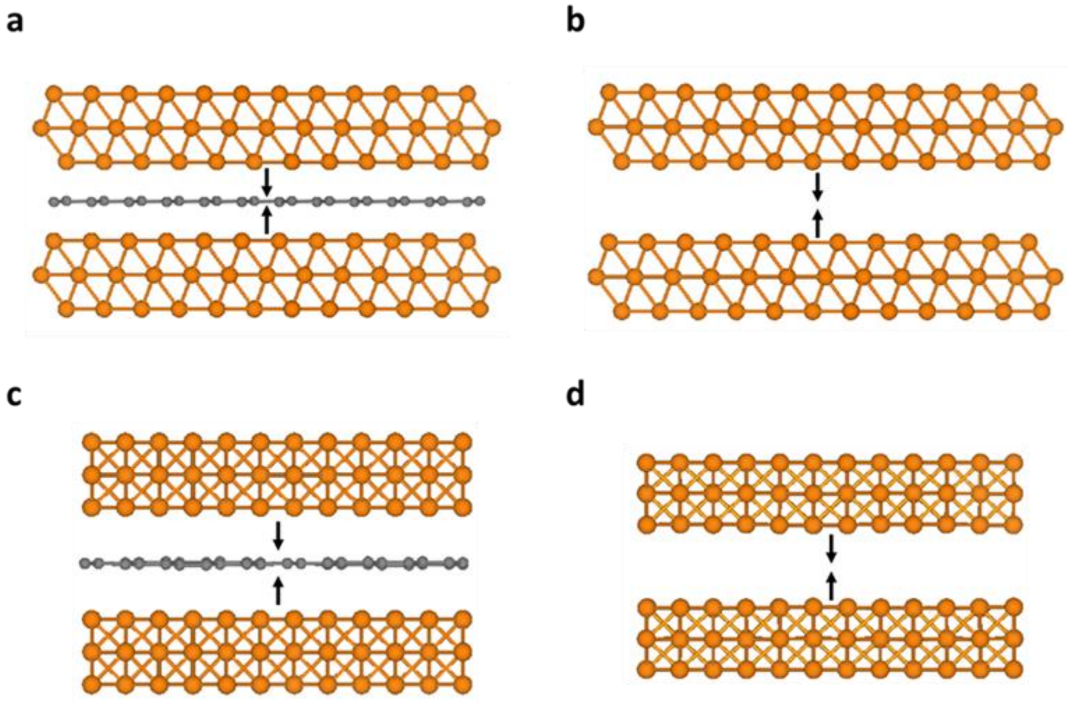

Fig. S39 | Atomic structures for binding between two Cu slabs of (111) surface. a-b, Binding between two Cu slabs of Cu (111) surface **(a)** with and **(b)** without C layer. **c-d,** Binding between two Cu slabs of Cu (100) surface **(c)** with and **(d)** without C layer**.** Orange and gray spheres are copper and carbon atoms, respectively.

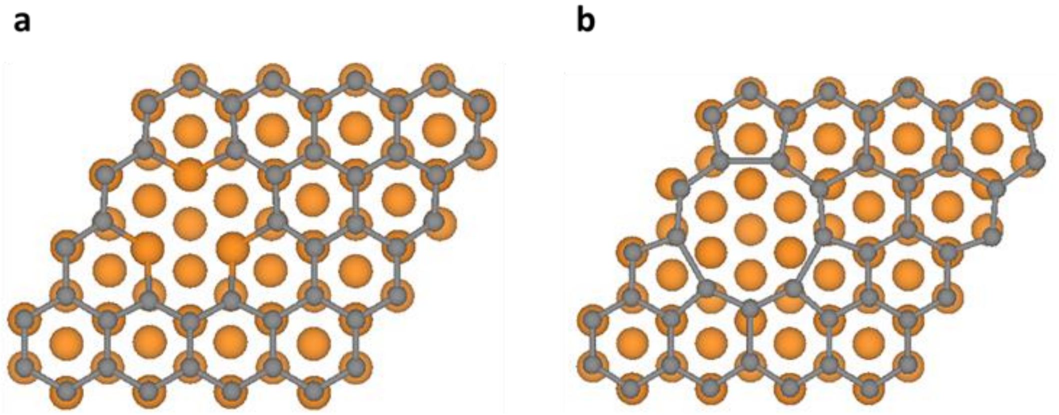

Fig. S40 | Atomic structure of Cu (111) surface encapsulated with defective C layer. a, C on C layer forms bond with Cu. **b,** C on C layer forms bond with C. Orange and gray spheres are copper and carbon atoms, respectively.

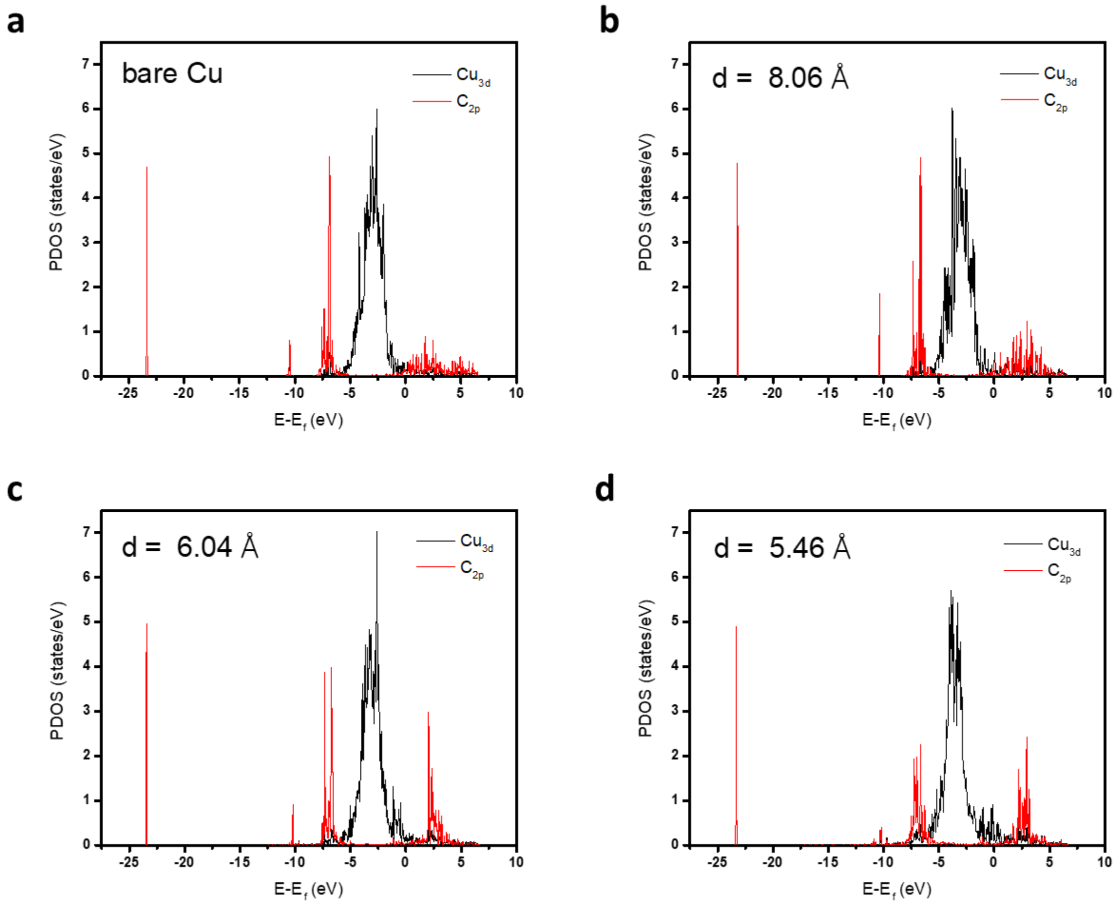

Fig. S41 | PDOS plot of Cu *3d* orbital and C *2p* orbital in CO-adsorbed Cu surface. **a-d,** PDOS plot in CO-adsorbed **(a)** bare Cu surface, Cu surface with C layer having pore size of **(b)** 8.06 Å, **(c)** 6.04 Å, **(d)** 5.46 Å.

a

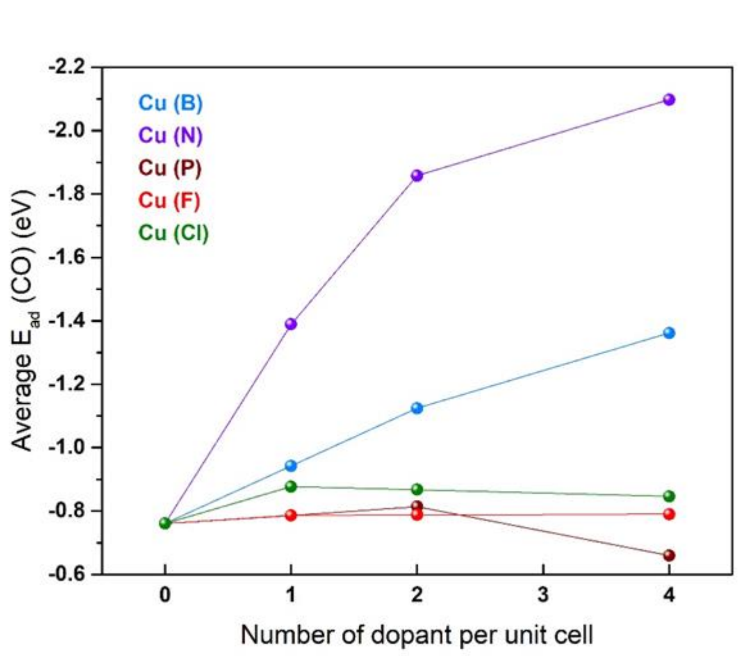

Fig. S42 | Average CO adsorption energy as a function of the number of dopant per unit cell on (**a**) 100 surface and (**b**) 111 surface of boron doped (Cu (B)), nitrogen doped (Cu (N)), phosphorus doped (Cu (P)), fluorine doped (Cu (F)), and chlorine doped (Cu (Cl)) Cu.

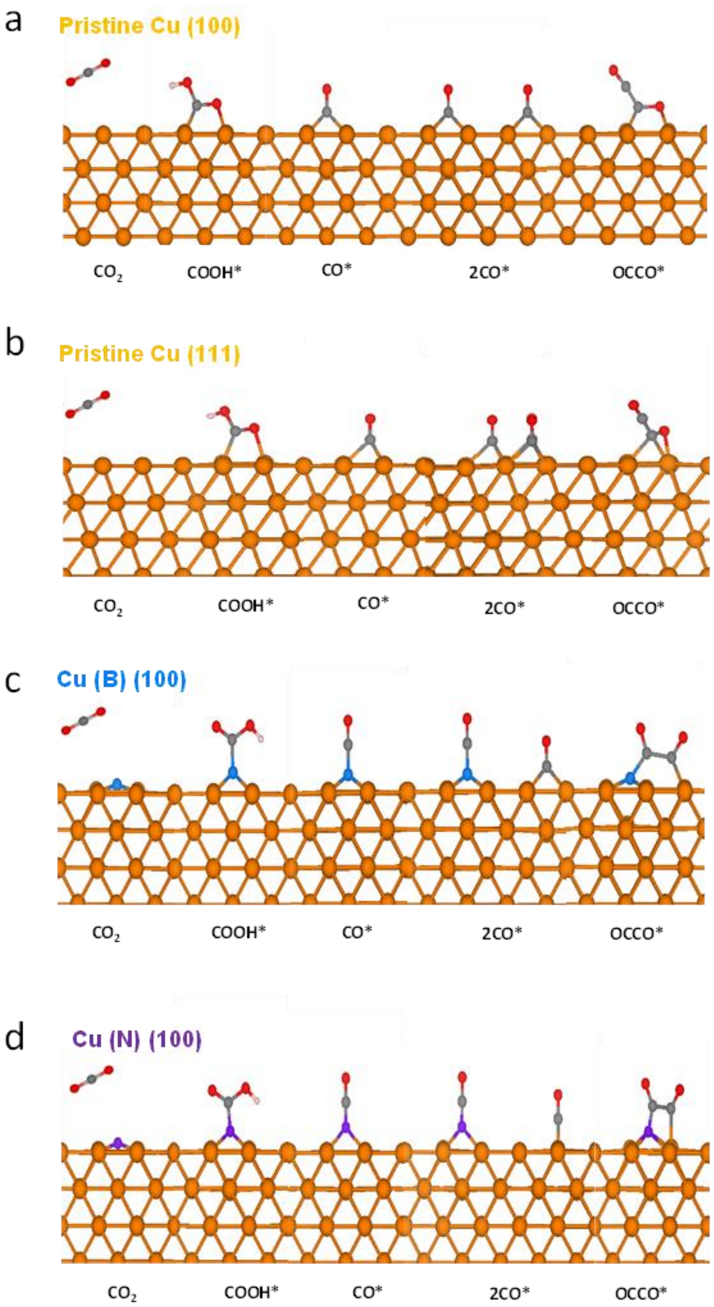

Fig. S43 | Schematic diagram of reaction pathway for CO_2 reduction on CO and CO dimerization on **(a)** pristine Cu (100), (**b**) pristine Cu (111), (**c**) Cu (B) (100), and (**d**) Cu (N) (100). Orange, blue, purple, gray, red, and white atoms are Cu, B, N, C, O, and H, respectively.

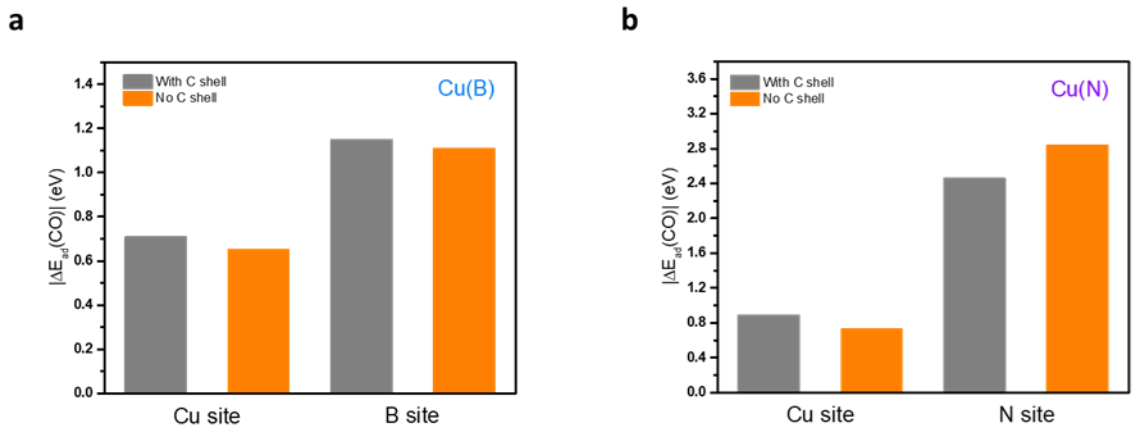

Fig. S44 | CO binding energy on Cu site and B / N site of Cu (B) and Cu (N) surface with porous C layer. a, Absolute value of CO binding energy for Cu (B) surface. **b,** Absolute value of CO binding energy for Cu (N) surface. The pore size of C layer was 8.06 Å

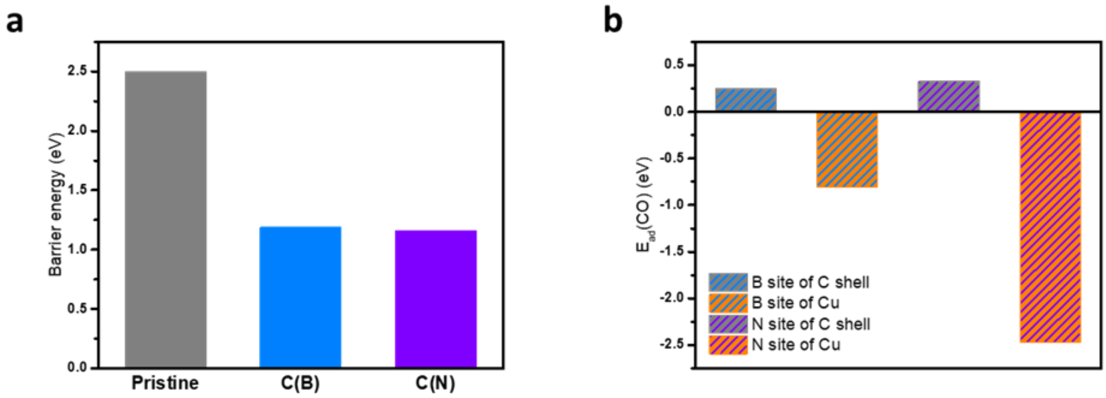

Fig. S45 | Investigation of B and N doping in C shell. a, Barrier energy of CO_2 reduction to CO on pristine graphene (pristine), B doped graphene (C (B)), and N doped graphene (C (N)). **b,** CO adsorption energy on B site and N site of C shell and Cu surface

Table S1 | Thermodynamic calculations on Boudouard reaction. Detailed value of product prediction in Fig. 1 (b). The unit of product is mole.

Temperature (K)	CO_2	CO	C
273	1	1.25E-12	1
323	1	4.44E-10	1
373	1	3.32E-08	1
423	1	9.00E-07	1
473	0.99999	1.22E-05	0.99999
523	0.9999	1.01E-04	0.99995
573	0.99943	5.73E-04	0.99971
623	0.99753	0.00247	0.99876
673	0.99148	0.00852	0.99572
723	0.97539	0.02461	0.98754
773	0.93885	0.06114	0.96846
823	0.8673	0.1327	0.92893
873	0.74708	0.25292	0.85523
923	0.57806	0.42194	0.73262
973	0.38746	0.61254	0.55851
1023	0.22261	0.77739	0.36416
1073	0.11388	0.88612	0.20448
1123	0.05549	0.94451	0.10515

1173	0.0272	0.9728	0.05296
1223	0.01379	0.98621	0.0272
1273	0.0073	0.9927	0.0145
1323	0.00404	0.99596	0.00805
1373	0.00233	0.99767	0.00466
1423	0.0014	0.9986	0.0028
1473	8.72E-04	0.99913	0.00174

Table S2 | Thermodynamic calculations on Boudouard reaction. Detailed value of product prediction in Fig. 1 (c). The unit of energy value is kJ.

Temperature (K)	1×10^{-5} atm	1×10^{-4} atm	1×10^{-3} atm	1×10^{-2} atm	1×10^{-1} atm	1 atm	1×10^{-8} atm	1×10^{-7} atm	1×10^{-6} atm
273	-98.3951	-103.622	-108.848	-114.075	-119.301	-124.528	-82.7154	-87.942	-93.1685
293	-92.9834	-98.5929	-104.202	-109.812	-115.421	-121.031	-76.1551	-81.7645	-87.374
313	-87.554	-93.5464	-99.5387	-105.531	-111.523	-117.516	-69.577	-75.5693	-81.5617
333	-82.1098	-88.4851	-94.8603	-101.236	-107.611	-113.986	-62.9841	-69.3594	-75.7346
353	-76.6536	-83.4117	-90.1698	-96.928	-103.686	-110.444	-56.3792	-63.1373	-69.8954
373	-71.1875	-78.3285	-85.4696	-92.6106	-99.7517	-106.893	-49.7644	-56.9054	-64.0465
393	-65.7137	-73.2376	-80.7615	-88.2855	-95.8094	-103.333	-43.1419	-50.6658	-58.1897
413	-60.2337	-68.1406	-76.0474	-83.9542	-91.861	-99.7679	-36.5132	-44.4201	-52.3269
433	-54.7492	-63.0389	-71.3286	-79.6184	-87.9081	-96.1978	-29.88	-38.1697	-46.4595
453	-49.2613	-57.9339	-66.6065	-75.2792	-83.9518	-92.6244	-23.2434	-31.916	-40.5887
473	-43.7711	-52.8266	-61.8822	-70.9377	-79.9932	-89.0487	-16.6046	-25.6601	-34.7156
493	-38.2796	-47.718	-57.1565	-66.5949	-76.0333	-85.4717	-9.9644	-19.4028	-28.8412
513	-32.7876	-42.6089	-52.4302	-62.2515	-72.0729	-81.8942	-3.3236	-13.145	-22.9663
533	-27.2957	-37.4999	-47.7041	-57.9084	-68.1126	-78.3168	3.3169	-6.8873	-17.0915
553	-21.8046	-32.3917	-42.9788	-53.5659	-64.153	-74.7401	9.9568	-0.6303	-11.2175
573	-16.3147	-27.2847	-38.2547	-49.2247	-60.1947	-71.1647	16.5954	5.6254	-5.3447
593	-10.8264	-22.1793	-33.5322	-44.8852	-56.2381	-67.591	23.2323	11.8794	0.5265
613	-5.3403	-17.0761	-28.8119	-40.5477	-52.2835	-64.0193	29.8672	18.1314	6.3955

633	0.1436	-11.9751	-24.0938	-36.2125	-48.3312	-60.4499	36.4997	24.381	12.2623
653	5.6247	-6.8769	-19.3785	-31.8801	-44.3817	-56.8833	43.1295	30.6279	18.1263
673	11.103	-1.7814	-14.6659	-27.5504	-40.4349	-53.3194	49.7565	36.872	23.9875
693	16.5783	3.3109	-9.9565	-23.2239	-36.4913	-49.7587	56.3805	43.1131	29.8457
713	22.0503	8.4	-5.2503	-18.9006	-32.5508	-46.2011	63.0012	49.3509	35.7006
733	27.519	13.4858	-0.5474	-14.5806	-28.6138	-42.647	69.6185	55.5853	41.5522
753	32.9841	18.568	4.152	-10.2641	-24.6802	-39.0963	76.2324	61.8163	47.4002
773	38.4457	23.6467	8.8477	-5.9512	-20.7502	-35.5492	82.8427	68.0437	53.2447
793	43.9036	28.7218	13.5399	-1.642	-16.8239	-32.0058	89.4493	74.2674	59.0855
813	49.3578	33.7931	18.2283	2.6635	-12.9013	-28.466	96.0522	80.4874	64.9226
833	54.8083	38.8606	22.9129	6.9652	-8.9824	-24.9301	102.6513	86.7036	70.7559
853	60.2549	43.9243	27.5937	11.2632	-5.0674	-21.398	109.2466	92.916	76.5855
873	65.6977	48.9842	32.2707	15.5573	-1.1562	-17.8697	115.8381	99.1246	82.4111
893	71.1366	54.0402	36.9439	19.8475	2.7511	-14.3452	122.4257	105.3293	88.233
913	76.5717	59.0924	41.6131	24.1339	6.6546	-10.8247	129.0094	111.5302	94.0509
933	82.0028	64.1407	46.2785	28.4164	10.5542	-7.308	135.5893	117.7272	99.865
953	87.4302	69.1851	50.9401	32.695	14.4499	-3.7951	142.1653	123.9203	105.6752
973	92.8536	74.2257	55.5977	36.9698	18.3418	-0.2861	148.7375	130.1095	111.4816
993	98.2733	79.2624	60.2515	41.2407	22.2298	3.219	155.3058	136.295	117.2841
1013	103.689	84.2953	64.9015	45.5078	26.114	6.7203	161.8703	142.4765	123.0828
1033	109.101	89.3244	69.5477	49.7711	29.9944	10.2178	168.431	148.6543	128.8777
1053	114.5092	94.3497	74.1901	54.0306	33.871	13.7115	174.9878	154.8283	134.6687

1073	119.9136	99.3712	78.8287	58.2863	37.7438	17.2014	181.5409	160.9985	140.456
1093	125.3142	104.3889	83.4636	62.5382	41.6129	20.6876	188.0903	167.1649	146.2396
1113	130.7112	109.4029	88.0947	66.7865	45.4782	24.17	194.6359	173.3276	152.0194
1133	136.1044	114.4132	92.7221	71.031	49.3398	27.6487	201.1778	179.4866	157.7955
1153	141.4939	119.4199	97.3458	75.2718	53.1978	31.1238	207.716	185.642	163.5679
1173	146.8798	124.4229	101.9659	79.509	57.0521	34.5951	214.2506	191.7936	169.3367
1193	152.262	129.4222	106.5824	83.7426	60.9027	38.0629	220.7815	197.9417	175.1019
1213	157.6407	134.418	111.1953	87.9725	64.7498	41.5271	227.3089	204.0861	180.8634
1233	163.0158	139.4102	115.8046	92.1989	68.5933	44.9877	233.8327	210.227	186.6214
1253	168.3873	144.3988	120.4103	96.4218	72.4333	48.4448	240.3529	216.3644	192.3759
1273	173.7554	149.384	125.0126	100.6411	76.2697	51.8983	246.8696	222.4982	198.1268

Table S3 | Thermodynamic calculations on product prediction. Detailed value of product prediction in Fig. S1 (a). The unit of all values is mole.

input O_2	CO	CO_2	C	Cu	Cu_2O	CuO
0	--	--	0.00733	5.46E-04	--	--
3.13E-05	6.25E-05	6.35E-09	0.00726	5.46E-04	--	--
9.38E-05	1.87E-04	5.71E-08	0.00714	5.46E-04	--	--
1.56E-04	3.12E-04	3.12E-04	0.00701	5.46E-04	--	--
3.13E-04	6.24E-04	6.33E-07	0.0067	5.46E-04	--	--
6.25E-04	0.00124	2.52E-06	0.00608	5.46E-04	--	--
9.38E-04	0.00186	5.65E-06	0.00546	5.46E-04	--	--
0.00125	0.00248	1.00E-05	0.00484	5.46E-04	--	--
0.00137	0.00271	1.19E-05	0.00461	5.46E-04	--	--
0.00137	0.00271	1.20E-05	0.0046	5.46E-04	--	--
0.00138	0.00273	1.21E-05	0.00458	5.46E-04	--	--
0.00139	0.00275	1.23E-05	0.00456	5.46E-04	--	--
0.00147	0.00292	1.38E-05	0.0044	5.46E-04	--	--
0.00158	0.00312	1.59E-05	0.00419	5.46E-04	--	--
0.00242	0.00477	3.70E-05	0.00252	5.46E-04	--	--
0.00348	0.00681	7.54E-05	4.43E-04	5.46E-04	--	--
0.00559	0.00347	0.00386	0	5.46E-04	--	--
0.00771	1.23E-10	0.00733	0	--	1.66E-05	5.13E-04

0.00559	0.00346	0.00386	0	5.46E-04	--	--
0.00563	0.0034	0.00393	0	5.46E-04	--	--
0.00594	0.00278	0.00455	0	5.46E-04	--	--
0.00625	0.00215	0.00518	0	5.46E-04	--	--
0.00656	0.00153	0.0058	0	5.46E-04	--	--
0.00688	9.00E-04	0.00643	0	5.46E-04	--	--
0.00719	2.75E-04	0.00705	0	5.46E-04	--	--
0.00734	1.10E-07	0.00733	0	4.72E-04	3.74E-05	--
0.0075	3.83E-05	0.00733	0	--	2.73E-04	--
0.00359	0.00703	8.03E-05	2.18E-04	5.46E-04		--
0.00375	0.00715	1.75E-04	0	5.46E-04		--
0.00406	0.00653	8.00E-04	0	5.46E-04		--
0.00438	0.0059	0.00142	0	5.46E-04		--
0.00469	0.00528	0.00205	0	5.46E-04		--
0.005	0.00465	0.00268	0	5.46E-04		--
0.00531	0.00403	0.0033	0	5.46E-04		--
0.00982	2.80E-11	0.00733	0			5.46E-04

Table S4 | Thermodynamic calculations on Boudouard reaction. Detailed value of Boudouard reaction in Fig. S1 (b).

Temperature (K)	ΔG (J)	Equilibrium constant
273	-124528	6.72E+23
293	-121031	3.78E+21
313	-117516	4.09E+19
333	-113986	7.60E+17
353	-110444	2.21E+16
373	-106893	9.33E+14
393	-103333	5.43E+13
413	-99767.9	4.16E+12
433	-96197.8	4.03E+11
453	-92624.4	4.79E+10
473	-89048.7	6.83E+09
493	-85471.7	1.14E+09
513	-81894.2	2.18E+08
533	-78316.8	4.74E+07
553	-74740.1	1.15E+07
573	-71164.7	3.07E+06
593	-67591	899487.3
613	-64019.3	285349.2

633	-60449.9	97376.97
653	-56883.3	35510.88
673	-53319.4	13756.71
693	-49758.7	5632.233
713	-46201.1	2425.67
733	-42647	1094.452
753	-39096.3	515.416
773	-35549.2	252.5141
793	-32005.8	128.3179
813	-28466	67.4505
833	-24930.1	36.58807
853	-21398	20.43542
873	-17869.7	11.72857
893	-14345.2	6.90447
913	-10824.7	4.16223
933	-7308	2.56542
953	-3795.1	1.61443
973	-286.1	1.036
993	3219	0.67712
1013	6720.3	0.45026
1033	10217.8	0.3043
1053	13711.5	0.20884

1073	17201.4	0.14541
1093	20687.6	0.10264
1113	24170	0.07339
1133	27648.7	0.05312
1153	31123.8	0.0389
1173	34595.1	0.0288
1193	38062.9	0.02155
1213	41527.1	0.01628
1233	44987.7	0.01242
1253	48444.8	0.00956
1273	51898.3	0.00742

Table S5 | Sample information for the thickness control of the C shell. Calcination conditions for quasi-graphitic C shell formation were described. The flow rate of CO and CO_2 during calcination was regulated using a mass flow controller. The entire calcination was conducted at 800℃ for 5 h. The heating rate was 3℃/min. The C shell thickness was statistically processed from 5 samples.

Sample	Calcination atmosphere	**C shell thickness (nm)**
Conventional Cu nanoparticles	Conventional Cu nanoparticles, No calcination	**0**
Limited CO supply	pO_2-controlled (50 mTorr), Thermally treated in closed chamber	**6.7**
Continuous CO supply	$pCO/pCO_2 = 3$, Thermally treated under continuous gas flow	**12.0**

**Table S6 | ** Radial distance, Cu-Cu coordination numbers fitting results of Cu with quasi-graphitic 6.7 nm and 12 nm thickness C shell before CO_2RR and during CO_2RR *operando* XAS analysis.

	**Shell**	**R (Å)**	**Coordination Number**	**ΔE_0 (eV)**	**σ^2 (Å^2)**
**Before CO_2RR of C-encapsulated Cu nanoparticles (6.7 nm C shell thickness)**	Cu-Cu	2.54	6.66	4.82	0.00769
**During CO_2RR of Cu nanoparticles without C shell (6.7 nm C shell thickness)**	Cu-Cu	2.55	7.38	6.60	0.00612
**Before CO_2RR of C-encapsulated Cu nanoparticles (12 nm C shell thickness)**	Cu-Cu	2.54	4.72	4.32	0.00791
**During CO_2RR of Cu nanoparticles without C shell (12 nm C shell thickness)**	Cu-Cu	2.52	5.08	1.65	0.00563

**Table S7 | ** Radial distance, Cu-Cu coordination numbers, and ΔE_0 of pristine Cu and Cu (X) (X: P, F, B, Cl, and N) calculated from EXAFS analysis.

	**Shell**	**R (Å)**	**Coordination Number**	**ΔE_0 (eV)**	**σ^2 (Å^2)**
**Pristine Cu**	Cu-Cu	2.54	8.97	4.33	0.00710
**Cu (P)**	Cu-Cu	2.54	8.56	4.57	0.00709
**Cu (F)**	Cu-Cu	2.54	8.23	4.35	0.00721
**Cu (B)**	Cu-Cu	2.54	8.54	4.44	0.00711
**Cu (Cl)**	Cu-Cu	2.54	8.67	4.48	0.00724
**Cu (N)**	Cu-Cu	2.54	8.73	4.46	0.00716

Table S8 | Zero-point energy (ZPE), enthalpic ($\int C_p dT$), and entropic (-TS) contribution to free energy of adsorbates on different surfaces. All values are given in eV.

	COOH*			CO*		
	ZPE	$\int C_p dT$	-TS	ZPE	$\int C_p dT$	-TS
Cu (100)	0.589	0.111	-0.215	0.164	0.079	-0.148
Cu (111)	0.612	0.108	-0.216	0.174	0.079	-0.149
Cu (B) (100)	0.620	0.113	-0.263	0.230	0.069	-0.153
Cu (B) (111)	0.608	0.113	-0.270	0.184	0.085	-0.203
Cu (N) (100)	0.655	0.106	-0.263	0.246	0.081	-0.186
Cu (N) (111)	0.657	0.109	-0.286	0.250	0.080	-0.183

Table S9 Chemical potentials (μ) of gas-phase molecules obtained by summing up electronic energy (E_{elec}), zero-point energy (ZPE), enthalpic ($\int C_p dT$), and entropic (-TS) contribution. [a] Data from this work. [b] Data from NIST chemistry webbook[3]. All energy values are given in eV.

	E_{elec} [a]	ZPE [a]	$\int C_p dT$ [b]	-TS [b]	μ	Fugacity (Pa)
H_2	-6.760	0.276	0.091	-0.407	-6.799	101,325
CO_2	-22.996	0.334	0.104	-0.666	-23.224	101,325
H_2O	-14.222	0.583	0.104	-0.588	-14.211	3,546

**Table S10 | ** Performance comparison table of CO_2-to-C_2H_4 electrocatalysts

Catalysts	F.E. (C_2H_4) (%)	-J (mA/cm^2)	Stability (hours)	Electrolyte	Reference
Our work	71.1	400	-	1 M KOH	
	62.5	10	180	0.1 M $KHCO_3$	
Cu (100) facet	65	520	65	7 M KOH	1
Cu (B)	52±2	70	40	0.1 M KCl	4
Hydroxide-mediated Cu	70	500	150	7 M KOH	5
Porous Cu	38.6	653	2	1 M KOH	6
F-modified Cu	65	1600	40	1 M KOH	7
Dealloyed Cu-Al	80	400	50	1 M KOH	8
Sputtered Cu	50	100	100	0.1 M $KHCO_3$	9
Molecular-tuned Cu	72	230	190	1 M $KHCO_3$	10
Branched Cu oxide	70	20	12	0.1 M $KHCO_3$	11

References

1 Wang, Y. et al. Catalyst synthesis under CO_2 electroreduction favours faceting and promotes renewable fuels electrosynthesis. *Nat. Catal.,* **3**, 98-106, doi:10.1038/s41929-019-0397-1 (2019).

2 Lee, S.-Y. *et al.* Bonding structure and etching characteristics of amorphous carbon for a hardmask deposited by DC sputtering. *Carbon,* **154**, 277-284, doi:10.1016/j.carbon.2019.08.013 (2019).

3 Afeefy, H., Liebman, J. & Stein, S. Neutral thermochemical data. In Linstrom, P. & Mallard, W. (eds.) NIST Chemistry WebBook, NIST Standard Reference Database Number 69 (National Institute of Standards and Technology, Gaithersburg MD, USA, 2010)

4 Zhou, Y. et al. Dopant-induced electron localization drives CO_2 reduction to C_2 hydrocarbons. *Nat. Chem.,* **10**, 974-980, doi:10.1038/s41557-018-0092-x (2018).

5 Dinh, C.-T. et al. CO_2 electroreduction to ethylene via hydroxide-mediated copper catalysis at an abrupt interface. *Science,* **360**, 783-787, doi:10.1126/science.aas9100 (2018).

6 Lv, J. J. et al. A Highly Porous Copper Electrocatalyst for Carbon Dioxide Reduction. *Adv. Mater.,* **30**, e1803111, doi:10.1002/adma.201803111 (2018).

7 Ma, W. et al. Electrocatalytic reduction of CO_2 to ethylene and ethanol through hydrogen-assisted C–C coupling over fluorine-modified copper. *Nat. Catal.,* **3**, 478-487, doi:10.1038/s41929-020-0450-0 (2020).

8 Zhong, M. et al. Accelerated discovery of CO_2 electrocatalysts using active machine learning. *Nature*, **581**, 178-183, doi:10.1038/s41586-020-2242-8 (2020).

9 Gabardo, C. M. et al. Continuous Carbon Dioxide Electroreduction to Concentrated Multi-carbon Products Using a Membrane Electrode Assembly. *Joule*, **3**, 2777-2791, doi:10.1016/j.joule.2019.07.021 (2019).

10 Li, F. et al. Molecular tuning of CO_2-to-ethylene conversion. Nature, **577**, 509-513, doi:10.1038/s41586-019-1782-2 (2020).

11 Kim, J. et al. Branched Copper Oxide Nanoparticles Induce Highly Selective Ethylene Production by Electrochemical Carbon Dioxide Reduction. *J. Am. Chem. Soc.,* **141**, 6986-6994, doi:10.1021/jacs.9b00911 (2019).
